# Mitochondria regulate proliferation in adult cardiac myocytes

**DOI:** 10.1172/JCI165482

**Published:** 2024-05-09

**Authors:** Gregory B. Waypa, Kimberly A. Smith, Paul T. Mungai, Vincent J. Dudley, Kathryn A. Helmin, Benjamin D. Singer, Clara Bien Peek, Joseph Bass, Lauren Nelson, Sanjiv J. Shah, Gaston Ofman, J. Andrew Wasserstrom, William A. Muller, Alexander V. Misharin, G.R. Scott Budinger, Hiam Abdala-Valencia, Navdeep S. Chandel, Danijela Dokic, Elizabeth Bartom, Shuang Zhang, Yuki Tatekoshi, Amir Mahmoodzadeh, Hossein Ardehali, Edward B. Thorp, Paul T. Schumacker

**Affiliations:** 1 Department of Pediatrics,; 2 Stanley Manne Children’s Research Institute of the Ann and Robert H. Lurie Children’s Hospital of Chicago,; 3 Department of Medicine,; 4 Department of Biochemistry and Molecular Genetics, and; 5 Department of Pathology, Northwestern University Feinberg School of Medicine, Chicago, Illinois, USA.

**Keywords:** Cardiology, Metabolism, Bioenergetics, Cardiovascular disease, Mitochondria

## Abstract

Newborn mammalian cardiomyocytes quickly transition from a fetal to an adult phenotype that utilizes mitochondrial oxidative phosphorylation but loses mitotic capacity. We tested whether forced reversal of adult cardiomyocytes back to a fetal glycolytic phenotype would restore proliferative capacity. We deleted *Uqcrfs1* (mitochondrial Rieske iron-sulfur protein, RISP) in hearts of adult mice. As RISP protein decreased, heart mitochondrial function declined, and glucose utilization increased. Simultaneously, the hearts underwent hyperplastic remodeling during which cardiomyocyte number doubled without cellular hypertrophy. Cellular energy supply was preserved, AMPK activation was absent, and mTOR activation was evident. In ischemic hearts with RISP deletion, new cardiomyocytes migrated into the infarcted region, suggesting the potential for therapeutic cardiac regeneration. RNA sequencing revealed upregulation of genes associated with cardiac development and proliferation. Metabolomic analysis revealed a decrease in α-ketoglutarate (required for TET-mediated demethylation) and an increase in *S*-adenosylmethionine (required for methyltransferase activity). Analysis revealed an increase in methylated CpGs near gene transcriptional start sites. Genes that were both differentially expressed and differentially methylated were linked to upregulated cardiac developmental pathways. We conclude that decreased mitochondrial function and increased glucose utilization can restore mitotic capacity in adult cardiomyocytes, resulting in the generation of new heart cells, potentially through the modification of substrates that regulate epigenetic modification of genes required for proliferation.

## Abstract 2

Deletion of the nuclear-encoded mitochondrial gene *Uqcrfs1* in adult mouse hearts reverted heart cells to a perinatal phenotype and activated cell proliferation.

## Introduction

Mitochondria generate ATP through oxidative phosphorylation (OXPHOS), but they also participate in diverse biological functions that include redox signaling (1), metabolite signaling (2), calcium signaling (3), and the generation of stress signals that escape from the cell and act on distant tissues (4, 5). Mitochondria also play important roles in synthesizing biomolecules involved in epigenetic modification of histones and DNA (6). Finally, mitochondria are critical for producing substrates needed for lipid, protein, and nucleotide biosynthesis, which are required for the de novo generation of biomass in rapidly proliferating cells. In postmitotic cells of metabolically active tissues such as the heart, ATP production is considered to be the primary function of mitochondria. However, the extent to which other functions of mitochondria continue to contribute to cardiomyocyte function and phenotype in the mature heart is not completely understood.

In fetal hearts where the O_2_ tension is low, mitochondrial OXPHOS is minimally developed, and cardiomyocytes utilize glycolysis as their principal source of energy production (7, 8). Soon after birth, heart cells begin to mature by downregulating glycolysis and upregulating mitochondrial biogenesis, facilitating the increase in mitochondrial respiration that confers the ability in adults to sustain ATP production when metabolic demand increases, as occurs during exercise. Fetal and newborn heart cells are capable of undergoing mitotic cell division, but this capacity is lost soon after birth, as the cells undergo the metabolic shift from glycolysis to OXPHOS (7, 9). Some evidence suggests that maintenance of glycolysis may favor the retention of a proliferative state (10, 11). If this shift from glycolysis to mitochondrial respiration were responsible for the transition from a proliferation-competent to an adult postmitotic state in the heart (8), then it is conceivable that forcing an adult heart to shift away from mitochondrial oxidative function and toward glycolysis could mimic conditions in the perinatal heart and restore its proliferative capacity. However, it is widely accepted that heart mitochondrial OXPHOS is essential for cardiac function and survival in adults, so forcing a sudden return to glycolysis would be expected to induce lethal cardiac failure. We sought to test this question by genetically disrupting mitochondrial function in adult mouse hearts by deleting the *Uqcrfs1* gene, which encodes the Rieske iron-sulfur protein (RISP), a component of complex III that is required for electron transfer and proton translocation. Previous studies have shown that this gene is dispensable for cell survival in other tissues and have demonstrated the importance of mitochondria for functions other than ATP production (12, 13). Given the high mitochondrial density in adult heart cells, we reasoned that genetic deletion of *Uqcrfs1* would cause a slow transition toward glycolysis as mitochondrial turnover leads to the disappearance of the preexisting RISP protein, potentially offering ample opportunity for the cells to adjust to that transition.

Here we report that adult mouse cardiomyocytes in which the mitochondrial electron transport chain (ETC) has been diminished by RISP deletion maintain energy stores, fail to develop bioenergetic stress, and continue to support mouse survival until eventual depletion of RISP leads to a lethal loss of OXPHOS. Remarkably, the postmitotic cardiomyocytes returned to the cell cycle, causing the hearts to undergo growth in a hyperplastic remodeling response where cellular hypertrophy, fibrosis, and inflammation were absent. These findings indicate that mitochondria, beyond ATP production, control adult cardiomyocyte cell fate and function.

## Results

### Conditional knockout of the Uqcrfs1 gene causes a decline in mitochondrial function and a switch to glycolysis.

**Figure 1:**
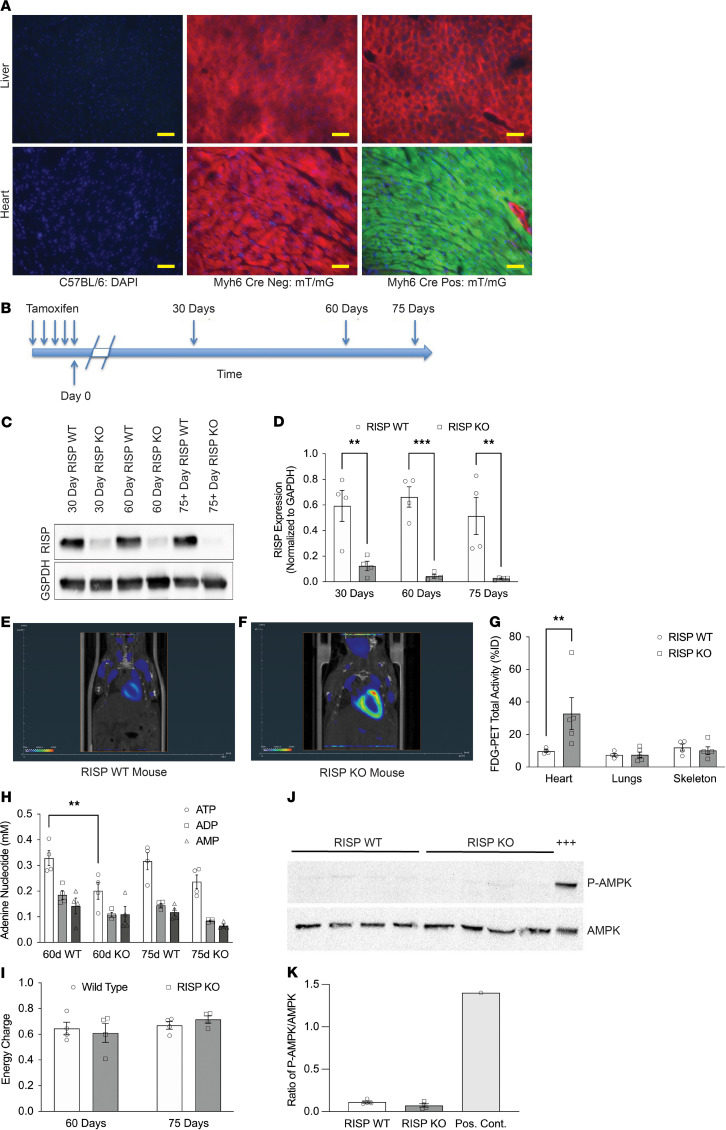
Cardiac RISP KO and energy supply in mice. (**A**) Adult mT/mG reporter mice carrying the Myh6-Cre transgene were given tamoxifen. After 14 days, liver and heart were removed and analyzed for evidence of Cre-mediated conversion from red to green fluorescent protein. Liver expressed only red fluorescence, whereas cardiac myocytes expressed green, indicating Cre activity. Scale bars: 50 μm. (**B**) RISP-KO and -WT mice were given tamoxifen and then evaluated at 30, 60, and 75 days. (**C** and **D**) Immunoblotting heart lysates for RISP protein revealed a progressive loss, with virtually complete depletion by 60 days; *n* = 4 mice per condition, mean ± SEM, 2-way ANOVA with a Holm-Šídák multiple-comparison test (2W-ANOVA-Šidák’s). (**E** and **F**) Representative FDG-PET assessment in RISP-WT and -KO mouse hearts. (**G**) Quantitative analysis of FDG utilization. The percentage injected dose (%ID) of FDG for each tissue was calculated by division of the total PET signal found in the region of interest by the injected dose for each mouse; *n* = 4–5 mice per condition, mean ± SEM, unpaired 2-tailed *t* test. (**H**) Adenine nucleotide levels in snap-frozen hearts from RISP-WT and -KO mice at 60 and 75 days after tamoxifen; *n* = 4 mice per condition, mean ± SEM, 2W-ANOVA-Šidák’s. (**I**) Adenine nucleotide energy charge in snap-frozen hearts from RISP-WT and -KO mice at 60 and 75 days after tamoxifen; *n* = 4 mice per condition, mean ± SEM, 2W-ANOVA-Šidák’s. (**J** and **K**) Assessment of AMPK activation in snap-frozen hearts from RISP-WT and -KO mice at 60 and 75 days after tamoxifen. Positive control was rapidly excised and cooled before freezing, rather than snap-frozen in situ; *n* = 4 mice per condition, mean ± SEM, unpaired 2-tailed *t* test. **P < 0.01, ***P < 0.001.

The *Uqcrfs1* gene encodes RISP, a component of complex III required for electron transfer and proton translocation. To disrupt complex III function, mice with homozygous floxed alleles of *Uqcrfs1* (RISP^fl/fl^) (14) were bred with heterozygous mice carrying a cardiac-specific α-myosin heavy chain promoter–driven, tamoxifen-inducible Cre recombinase transgene (Myh6-Cre) (15), yielding RISP^fl/fl^ ± Myh6-Cre (RISP-KO and RISP-WT, respectively) offspring in the C57BL/6 genetic background. To assess the efficacy of cardiac Myh6-Cre activity, tamoxifen was administered to Myh6-Cre adult mT/mG fluorescent reporter mice (16). At 14 days after tamoxifen, tissues were analyzed for evidence of Cre-mediated conversion from constitutive expression of red fluorescent protein to green. As expected, in liver, where Myh6-Cre expression is absent, only the red fluorescent protein was detected (Figure 1A). By contrast, following Cre activation, heart cells uniformly demonstrated green fluorescence; the only cells still exhibiting red fluorescence were capillaries and coronary blood vessels, indicating high efficiency of in vivo recombination in adult cardiomyocytes. Next, RISP-WT and -KO mice received tamoxifen between 5 and 7 weeks of age; levels of residual RISP protein in the heart were assessed at 30, 60, and 75 days thereafter (Figure 1B). RISP protein in heart lysates was significantly decreased at 30 days; by 60 days the protein was more than 90% depleted, and by 75 days it was minimally detectable in RISP KO compared with RISP WT (Figure 1, C and D). Loss of RISP disrupts complex III of the ETC and abolishes OXPHOS by preventing electrons generated at complex I or II from reaching cytochrome *c* oxidase (complex IV). To assess the expected decline in mitochondrial function, hearts were rapidly harvested at 60 days, and respiration was measured in isolated mitochondria (Supplemental Figure 1A; supplemental material available online with this article; https://doi.org/10.1172/JCI165482DS1). These studies revealed a significant but incomplete decrease in rotenone-inhibited, succinate-driven respiration by complex III in the RISP-KO mice. However, absolute respiration at complex IV was not different when electrons were delivered directly to cytochrome *c* (thus bypassing complex III), indicating that the decline in function was limited to complex III. In mitochondria from 75-day hearts, respiration at complex III relative to complex IV was nearly abolished (Supplemental Figure 1B). In cells lacking a functional ETC, mitochondrial inner membrane potential (ΔΨm) is sustained by mitochondrial ATP synthase, which operates in reverse by taking up ATP derived from cytosolic glycolysis or substrate-level phosphorylation. Hydrolysis of that ATP in the matrix by complex V allows the ATP synthase to operate in reverse, sustaining ΔΨm by extruding protons across the inner membrane (17). To assess the effects of RISP depletion on mitochondrial potential, neonatal cardiomyocytes were isolated from RISP^fl/fl^ mice carrying constitutively active Cre under the control of the muscle creatine kinase promoter (MCK) and maintained in tissue culture for more than 14 days. Both RISP-WT and -KO cells exhibited polarized mitochondria. However, myxothiazol (2 μM), which inhibits complex III, caused a greater decrease in ΔΨm in WT cells, indicating that forward flux in the ETC was contributing to the maintenance of mitochondrial polarization. By contrast, RISP-deficient cells were better able to maintain potential during myxothiazol treatment, indicating that the ETC was not contributing to the maintenance of ΔΨm in cells depleted of RISP (Supplemental Figure 1C). RISP-WT cardiomyocytes survived in 15 mM galactose (without glucose), whereas RISP-KO cardiomyocytes detached from coverslips in galactose medium, confirming a dependence on glycolytic metabolism in those cells (data not shown). These results demonstrate that progressive loss of RISP in adult mouse cardiomyocytes leads to a decline in mitochondrial function, forcing a switch toward glycolysis. At 60 days after tamoxifen the ETC still retains partial function, but by 75 days a virtually complete loss of RISP forces a condition in which the cells must rely on glycolysis for survival.

The partial decline in complex III function in 60-day RISP-KO hearts was associated with increased cardiac glucose utilization, as assessed by ^18^F-fluordeoxyglucose utilization measured by positron emission tomography (FDG-PET) (Figure 1, E–G, and Supplemental Figure 1D). This was associated with a decrease in ATP concentration in rapidly frozen hearts (Figure 1H). However, there was no indication of bioenergetic deficiency, as the adenylate energy charge (([ATP] + 0.5[ADP])/([ATP] + [ADP] + [AMP])) was not diminished (Figure 1I and Supplemental Figure 1E). More convincingly, AMPK, a master sensor of cellular energy stores and regulator of energy usage (18), was not activated in 60-day control or RISP-KO hearts, as indicated by its lack of phosphorylation. By contrast, even brief ischemia during rapid heart harvest resulted in its phosphorylation and thus activation (Figure 1, J and K). Thus, 60-day RISP-KO hearts show a partial decline in ETC function and an accompanying increase in glucose utilization without evidence of bioenergetic crisis.

### RISP depletion in cardiomyocytes induces cardiomegaly without affecting cardiomyocyte size or ultrastructure.

**Figure 2:**
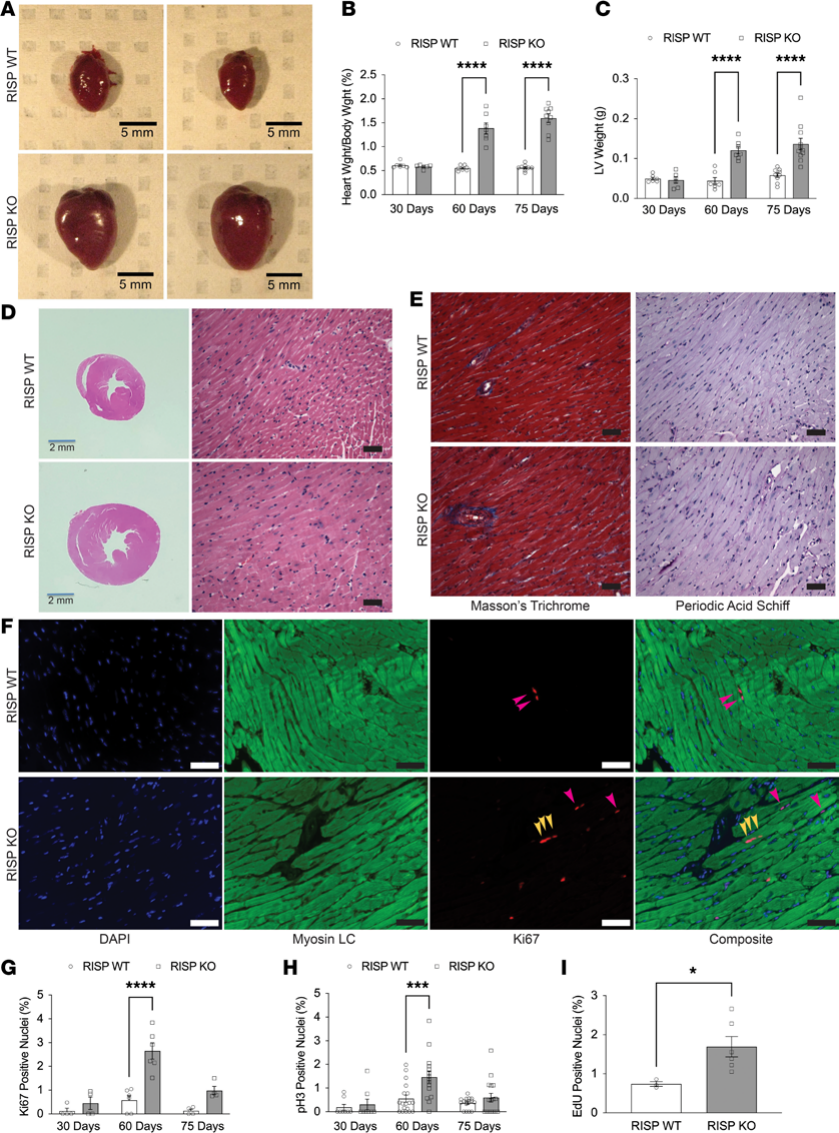
Cardiac RISP KO and subsequent remodeling in adult mice. (**A**) Heart size increased significantly at 75 days after tamoxifen in RISP-KO mice compared with WT controls. Scale bars: 5 mm. (**B**) HW/BW increased at 60 and 75 days after tamoxifen in RISP-KO mice compared with WT controls; *n* = 6–9 mice per condition, mean ± SEM, 2W-ANOVA-Šidák’s. (**C**) LV weight increased at 60 and 75 days after tamoxifen compared with WT; *n* = 6–9 mice per condition, mean ± SEM, 2W-ANOVA-Šidák’s. (**D**) Heart size was significantly increased in RISP-KO mice compared with WT, while cell morphology was indistinguishable between groups. Scale bars: 2 mm or 50 μm. (**E**) Cardiac fibrosis (Masson’s trichrome stain) was absent in RISP-KO hearts at 75 days after tamoxifen, and indistinguishable from WT. Cell diameter was assessed in PAS-stained heart sections. Scale bars: 50 μm. (**F**) Representative heart sections stained for DAPI, myosin light chain (LC), and Ki-67 in RISP-WT and -KO hearts 60 days after tamoxifen. Yellow and magenta arrowheads denote Ki-67–positive nuclei. In hearts from RISP-WT mice, most Ki-67–positive nuclei were colocalized to regions between cardiomyocytes and therefore not counted (magenta arrowheads). In RISP-KO hearts, only Ki-67–positive nuclei that colocalized with cardiomyocytes were counted (yellow arrowheads). Scale bars: 50 μm. (**G**) Ki-67–positive nuclei were more abundant in RISP-KO hearts at 60 days after tamoxifen compared with WT; *n* = 8–18 mice per condition, mean ± SEM, 2W-ANOVA-Šidák’s. (**H**) Phospho-H3–positive nuclei were more abundant in RISP-KO hearts at 60 days after tamoxifen compared with WT; *n* = 8–18 mice per condition, mean ± SEM, 2W-ANOVA-Šidák’s. (**I**) EdU-positive nuclei in cardiac sections from RISP-WT and -KO mice at 60 days after tamoxifen. EdU was administered by subcutaneous micro-osmotic pump, inserted at day 30; *n* = 3–6 mice per condition, mean ± SEM, unpaired 2-tailed *t* test. *P < 0.05, ***P < 0.001, ****P < 0.0001.

Hearts from RISP-KO mice exhibited a remarkable increase in size compared with those from RISP-WT littermates (Figure 2A). Cardiac remodeling was not evident at 30 days when RISP protein was still abundant, but by 60 days when RISP was significantly decreased, the heart weight/body weight ratios (HW/BW) had more than doubled (Figure 2B), while body weights did not differ (Supplemental Figure 2A). Left ventricular (LV) weight also increased progressively between the 30- and 60-day time points but less so between 60 and 75 days (Figure 2C). Mice began to die after approximately 75 days, possibly as a consequence of severe loss of OXPHOS as evidenced by earlier experiments (Supplemental Figure 1B). While cursory examination suggested that the LV changes were reminiscent of the remodeling response to pressure overload (19), parallel changes were seen in LV and right ventricle plus septum weights, indicating that remodeling encompassed the entire heart (Supplemental Figure 2B). Moreover, mean systemic arterial blood pressure was modestly decreased in awake RISP-KO compared with RISP-WT mice (Supplemental Figure 2C). Importantly, histological comparison between RISP KO and RISP WT even at 75 days revealed that despite the extensive cardiomegaly, no differences in cell morphology were evident (Figure 2D). Inflammatory cell infiltration was absent, and no fibrotic remodeling (Masson’s trichrome staining) was detected (Figure 2E). To quantify cell size, heart sections were subjected to periodic acid–Schiff (PAS) staining to identify cell margins, and cell widths across the LV wall were assessed by an unbiased image analysis process using ImageJ (NIH). Although LV wall thickness was modestly increased (Supplemental Figure 3A), cardiomyocyte width was not different from control (Supplemental Figure 3B), indicating an absence of cardiomyocyte hypertrophy. Moreover, cell counts across the LV wall were not increased (Supplemental Figure 3C). To further assess remodeling, wheat germ agglutinin staining was carried out (Supplemental Figure 3, D and E), and individual cell cross-sectional areas were measured in heart sections. Again, no difference in cell cross-sectional area between RISP-KO and -WT heart cells was detected, indicating an absence of hypertrophic remodeling (Supplemental Figure 3F). Counts of capillaries in the LV wall did increase (Supplemental Figure 3G). Contractile fiber ultrastructure (Supplemental Figure 4A), mitochondrial ultrastructure (Supplemental Figure 4B), and mitochondrial abundance (Supplemental Figure 4C), assessed by electron microscopy, were indistinguishable between WT and KO hearts. Collectively these data indicate that partial disruption of the mitochondrial ETC causes remodeling involving dramatic cardiomegaly, with increases in heart size but no change in cardiomyocyte size or ultrastructure.

### RISP depletion causes hyperplastic remodeling of the heart and an activation of cardiomyocyte proliferation.

These results suggested that the decline in ETC function and the increase in glycolysis are associated with a return of cardiomyocyte proliferation. To assess this, heart sections were costained for Ki-67, a nuclear marker of proliferation, and myosin light chain, a cardiomyocyte marker (Figure 2F). No difference from WT was detected at 30 days, but by 60 days there was clear evidence of proliferation in the RISP-KO cardiomyocytes (Figure 2G). Notably, evidence of cardiomyocyte proliferation was relatively abundant at 60 days but absent by 75 days. A similar pattern indicating increased mitosis in costained cardiomyocytes was seen with phospho–histone H3 nuclear staining (Figure 2H and Supplemental Figure 4D). Finally, to validate those results, RISP-KO and -WT mice were given 5-ethyny-2′-deoxyuridine (EdU) by subcutaneous micro-osmotic pump between 30 and 60 days; these studies revealed evidence of cardiomyocyte DNA synthesis, indicating that proliferation had been activated (Figure 2I). These results indicated that evidence of cardiomyocyte proliferation develops by 60 days but later halts in association with the development of severe ETC deficiency.

**Figure 3:**
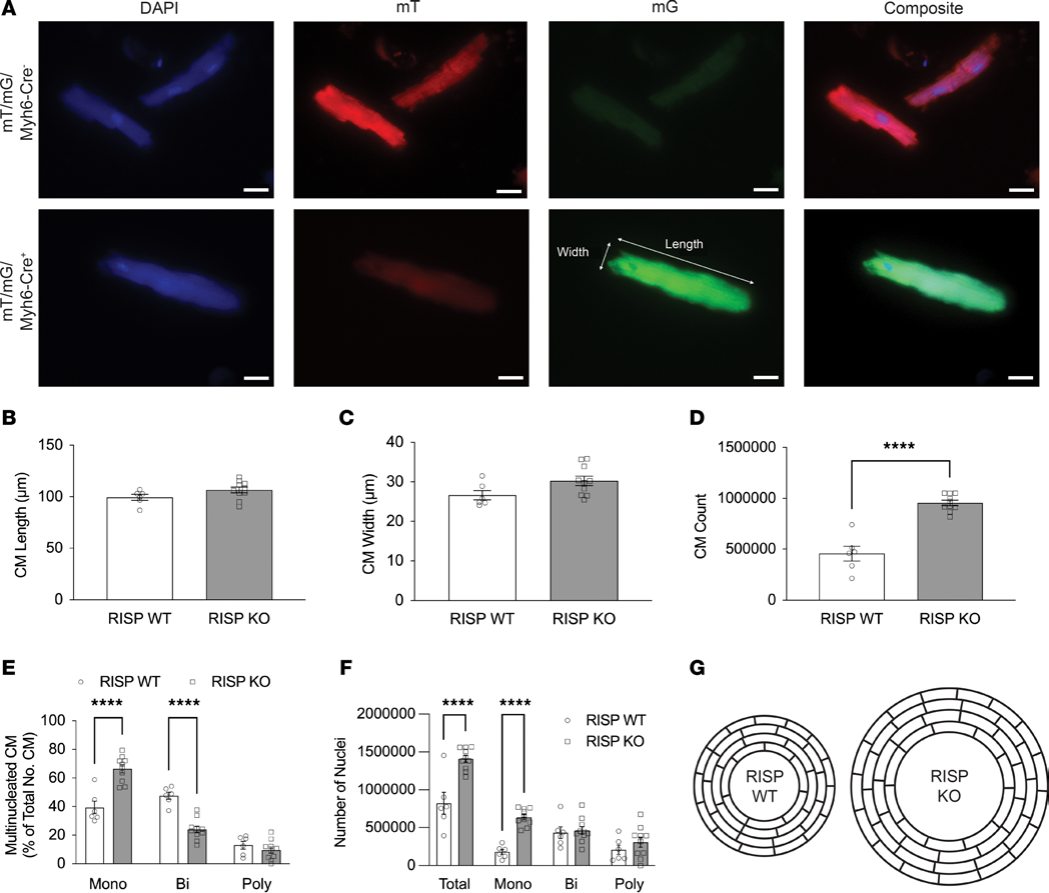
Cardiac RISP deletion causes cardiac hyperplasia. (**A**) Isolated, individual cardiomyocytes from mT/mG-RISP-WT or -KO mice 60 days after tamoxifen were stained with DAPI. Cardiomyocytes from mT/mG-RISP-WT mice maintained red fluorescence (mT), while cardiomyocytes from mT/mG-RISP-KO mice expressed green fluorescence (mG). The length and width of Cre-activated cardiomyocytes expressing mG were measured (ImageJ). Scale bars: 25 μm. (**B** and **C**) Cardiomyocyte length (**B**) and width (**C**). RISP KO had no effect on the size of cardiomyocytes compared with WT. (**D**) RISP KO caused cardiac hyperplasia compared with WT, as assessed by number of cardiomyocytes in hearts. Cardiomyocytes were counted and extrapolated to determine the number in the whole heart; *n* = 6–10 mice per condition, mean ± SEM, unpaired 2-tailed *t* test. (**E**) RISP KO increased the percentage of mononucleated and decreased the percentage of binucleated cardiomyocytes compared with WT. DAPI-stained cardiomyocytes were imaged and designated as mononucleated, binucleated, or polynucleated. Nuclei count is reported as percentage of assessed cardiomyocytes. (**F**) RISP KO increased the total number of nuclei compared with WT. Graph represents total number of nuclei per mouse heart and distribution of those nuclei across mono-, bi-, and polynucleated cardiomyocytes. For **B**, **C**, **E**, and **F**, approximately 40–45 cardiomyocytes per mouse were measured and averaged; *n* = 6–10 mice per condition, mean ± SEM, unpaired 2-tailed *t* test. ****P < 0.0001. (**G**) Diagram illustrating why cardiac wall thickness did not increase markedly in RISP-KO hearts undergoing hyperplastic remodeling. Cardiomyocytes appear to divide and grow in an end-to-end direction rather than side to side (with no difference in cell lengths), resulting in greater circumference of the heart wall without a large increase in LV wall thickness, a significant increase in the widths of cells, or an increase in number of cells across the LV free wall.

While the above findings support the conclusion that cardiomyocyte proliferation was activated, a more rigorous test requires quantification of cell numbers. To genetically identify cardiomyocytes, mT/mG fluorescent reporter mice (16) were bred with RISP-KO and RISP-WT mice. Adult mice received tamoxifen to label the cardiomyocytes with green fluorescence and, in the fl/fl mice, to delete RISP. Hearts were harvested after 60 days, fixed, and digested to compare cell numbers and cardiomyocyte dimensions (Figure 3A). Cardiomyocytes were distinguished from other cell types by their rod-shaped appearance and green fluorescence. No differences in cell length (Figure 3B) or width (Figure 3C) were detected, validating the absence of hypertrophy as evidenced by earlier experiments (Figure 2E and Supplemental Figure 3, B and D–F). However, the number of cardiomyocytes from the RISP-KO hearts increased from nearly 500,000 to approximately 1 million, validating the presence of hyperplastic remodeling (Figure 3D). DAPI costaining was used to quantify the number of nuclei per cell; this revealed an increase in the number of mononuclear and a decrease in the number of diploid cardiomyocytes in the RISP-KO mice compared with controls (Figure 3E). Thus, some of the new cells likely arose from cell division of binuclear cardiomyocytes in the heart. Consistent with the increased cardiomyocyte numbers, the total number of cardiomyocyte nuclei, as well as the number of mononuclear cardiomyocyte nuclei, increased in the RISP-KO hearts (Figure 3F). Thus, the proliferation of cardiomyocytes involved the division of existing binuclear into mononuclear cells, as well as de novo synthesis of new mononuclear cardiomyocytes, also evidenced by the increase in EdU-positive nuclei (Figure 2I). How could the number of cells in the heart have doubled when the number of cells across the LV wall did not change? We propose that cell division occurred along the long axis of the cardiomyocytes (end-to-end division) and that subsequent growth in the length of the daughter cells produced an increase in the circumferential dimension of the ventricular wall without increasing its lateral dimension (Figure 3G). This produced a dramatic increase in the size of the heart characterized by an enlarged ventricular chamber, a slight increase in LV wall thickness, and no evidence of cellular hypertrophy.

**Figure 4:**
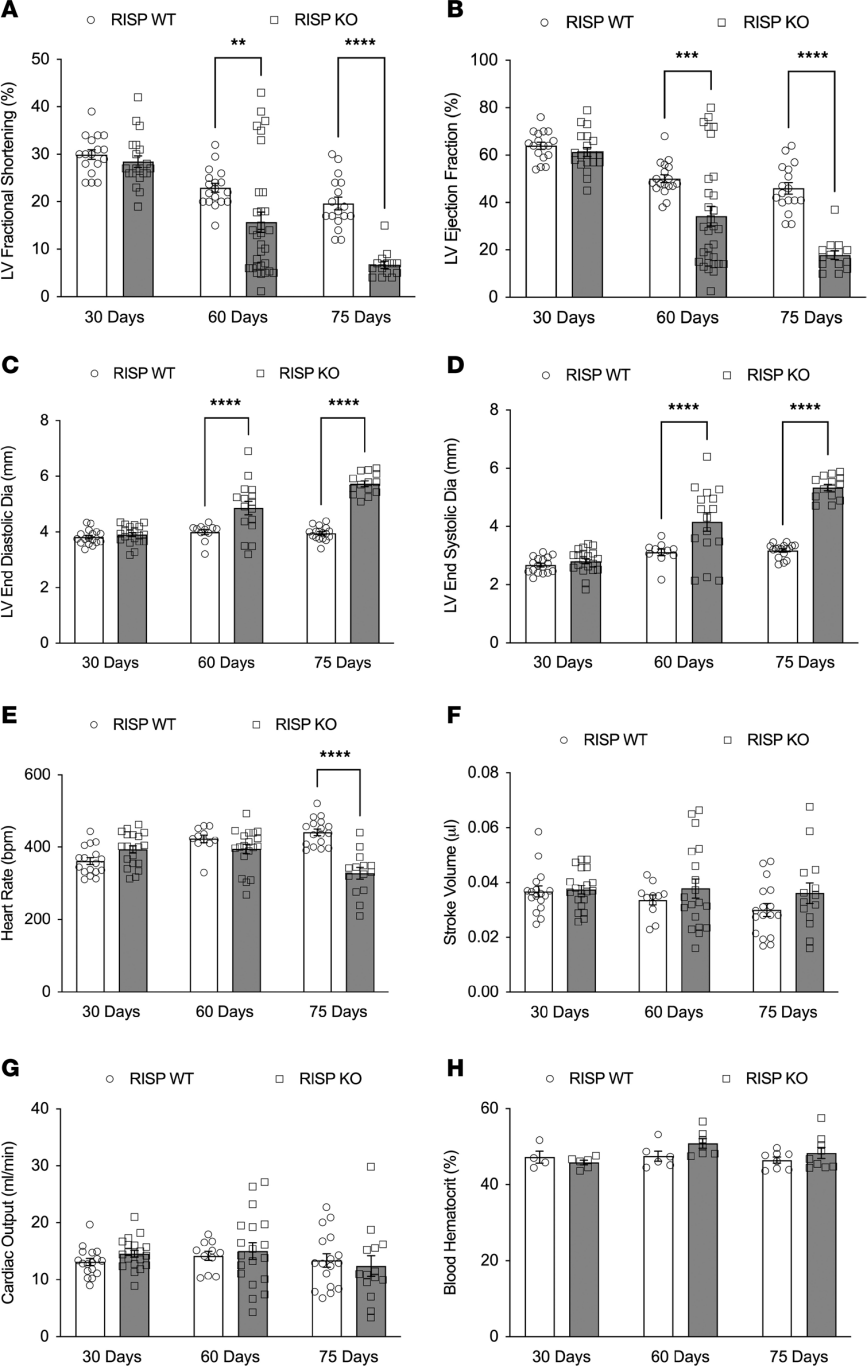
Cardiac function studies. (**A**) LV fractional shortening, assessed by echocardiography, decreased at 60 and 75 days after tamoxifen in RISP-KO hearts compared with WT; *n* = 14–31 mice per condition, mean ± SEM, 2W-ANOVA-Šidák’s. (**B**) LV ejection fraction decreased at 60 and 75 days after tamoxifen in RISP-KO compared with WT; *n* = 14–31 mice per condition, mean ± SEM, 2W-ANOVA-Šidák’s. (**C**) LV end-diastolic diameter increased at 60 and 75 days after tamoxifen in RISP-KO compared with WT; *n* = 11–20 mice per condition, mean ± SEM, 2W-ANOVA-Šidák’s. (**D**) LV end-systolic diameter increased at 60 and 75 days after tamoxifen in RISP-KO compared with WT; *n* = 11–20 mice per condition, mean ± SEM, 2W-ANOVA-Šidák’s. (**E**) Heart rate in RISP-WT and RISP-KO mice undergoing echocardiography at 30, 60, and 75 days after tamoxifen; *n* = 11–20 mice per condition, mean ± SEM, 2W-ANOVA-Šidák’s. (**F**) Stroke volume in RISP-WT and -KO mice at 30, 60, and 75 days after tamoxifen; *n* = 11–20 mice per condition, mean ± SEM, 2W-ANOVA-Šidák’s. (**G**) Cardiac output (product of heart rate and end-diastolic minus end-systolic diameter) was not different between RISP-WT and -KO hearts; *n* = 11–20 mice per condition, mean ± SEM, 2W-ANOVA-Šidák’s. (**H**) Hematocrit in RISP-WT and -KO mice at 30, 60, and 75 days after tamoxifen; *n* = 4–9 mice per condition, mean ± SEM, 2W-ANOVA-Šidák’s. **P < 0.01, ***P < 0.001, ****P < 0.0001.

Echocardiography assessments in anesthetized mice revealed a progressive decrease in LV fractional shortening (Figure 4A) and LV ejection fraction (Figure 4B). Both LV end-diastolic (Figure 4C) and end-systolic diameters (Figure 4D) were increased. However, heart rate and stroke volume were maintained (Figure 4, E and F), so the calculated cardiac output was preserved (Figure 4G). Hematocrit was unaffected (Figure 4H). Thus, RISP-KO hearts continue to function with a partial loss of ETC function, although the decline in diastolic and systolic function became severe by 75 days.

### RISP KO–mediated cardiomyocyte proliferation induces heart regeneration in regions of ischemic injury.

**Figure 5:**
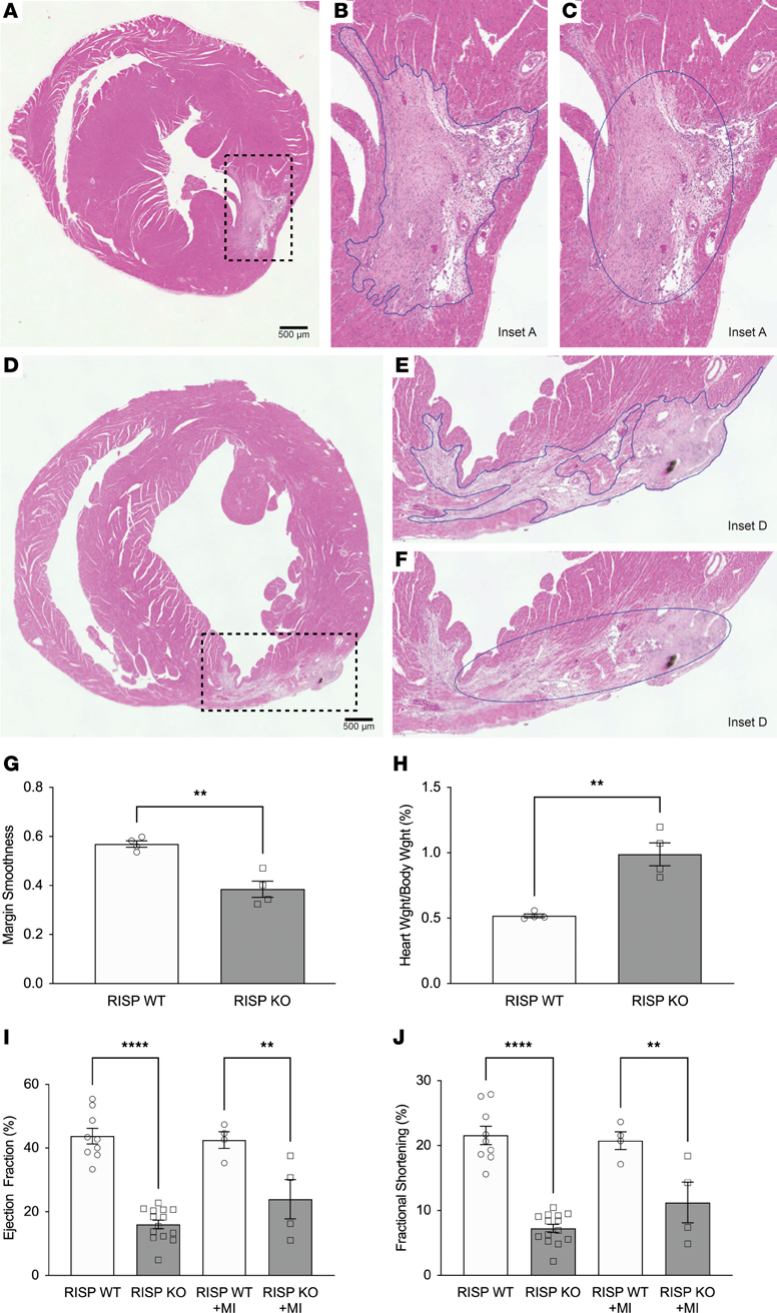
New cardiomyocytes in RISP KO infiltrate regions of ischemic damage after myocardial infarction. The left anterior descending coronary artery was permanently ligated 45 days after tamoxifen, creating a myocardial infarction (MI) in the LV. At 60 days, hearts were harvested, fixed, and serially sliced from apex to base. (**A** and **D**) Representative H&E-stained heart slices illustrating MI-induced scar regions in LV of RISP-WT and -KO mice, respectively. Scale bars: 500 μm. (**B** and **E**) Insets of **A** and **D**, respectively. ImageJ was used to trace the boundary between necrotic and non-necrotic tissue to appreciate the geometry of the necrotic tissue. Finger-like projections of dividing cardiomyocytes into the damaged tissue were observed in RISP-KO hearts (**E**) compared with RISP-WT hearts (**B**). (**C** and **F**) Representative ellipses generated by ImageJ designating the best-fit ellipse based on the boundaries traced in **B** and **E**, respectively. (**G**) Quantitative analysis of the smoothness of boundaries designating scar tissue, calculated by division of the measured parameters of the ellipses generated in **C** and **F** by the measured parameters traced in **B** and **E**, respectively. Boundaries traced in RISP-KO hearts were markedly rougher, with more finger-like projections into the injured regions. Injured regions were traced in 6–8 heart slices per mouse with *n* = 4 mice per condition, mean ± SEM, unpaired 2-tailed *t* test. (**H**) HW/BW of mice in MI studies demonstrated that MI did not affect hyperplastic remodeling induced by RISP KO; *n* = 4 mice per condition, mean ± SEM, unpaired 2-tailed *t* test. (**I** and **J**) Differences in LV ejection fraction (**I**) and fractional shortening (**J**) between RISP-WT and -KO mice were unaffected by the MI; *n* = 4–14 mice per condition, mean ± SEM, unpaired 2-tailed *t* test. **P < 0.01, ****P < 0.0001.

Can new cardiomyocytes repair myocardium damaged by ischemia? To test this, initial experiments were carried out to determine whether ischemia produces an equivalent extent of injury in RISP-KO and -WT hearts. Hearts at 45 days were subjected to ischemia and reperfusion of the coronary artery to induce ischemic injury. After 48 hours, hearts were harvested, and the area of ischemia (infarct size) relative to the area at risk was quantified (Supplemental Figure 5, A–C). These studies revealed equivalent injury in both groups, indicating that RISP KO does not affect the extent of ischemic injury at that time point (Supplemental Figure 5D). Next, control and RISP-KO hearts were subjected to ligation of a distal segment of the coronary artery at 45 days and were harvested for analysis at 60 days. Cardiomyocyte division had previously been shown to be active between 45 and 60 days, so we sought to determine whether new cells generated during that period would migrate into the region of previous ischemic injury. Clear evidence of ischemic injury was observed in these hearts (Figure 5, A and D). The periphery of the injured area was outlined using ImageJ (Figure 5, B and E), and an ellipse with an equivalent area was generated (Figure 5, C and F). The ratio of the circumference of the ellipse to the circumference of the ischemic area was then calculated to provide an index of the irregularity in the periphery of the scar region for the 2 groups. Visual inspection revealed that projections of live cardiomyocytes were penetrating the injured area in the RISP-KO hearts, giving rise to the jagged and irregular periphery of the injured area summarized in Figure 5G. By contrast, the control hearts demonstrated a smoother and more confluent boundary to the injured area. Ischemic injury had no effect on the increase in HW/BW differences (Figure 5H). Cardiac function in the RISP-KO hearts was significantly impaired compared with that in WT hearts; that impairment was large compared with the effect of myocardial ischemia in WT controls (Figure 5, I and J). However, the severity of infarction was not sufficient by itself to decrease cardiac function. A less severe infarction was used in order to minimize lethality during the 48-hour postoperative period. As the new cells generated in the RISP-KO hearts were functionally impaired, they were unable to restore cardiac performance in the ischemic hearts. These findings reveal that newly formed cardiomyocytes have the potential to migrate into an area of the heart that has sustained ischemic injury. While this did not appear to reduce the fibrotic remodeling in the injured area, the proliferative response involves new cardiomyocytes repopulating heart regions damaged by ischemia.

### Hyperplastic remodeling of the heart involves cardiomyocyte proliferation.

To determine the lineage of new cells in the heart, mT/mG-RISP-KO and -WT mice were generated (16). Adult mouse hearts were analyzed 60 days after tamoxifen (Supplemental Figure 6A). If nonmyocyte progenitor cells had migrated from bone marrow to the heart and differentiated into cardiomyocytes, these new cells would have displayed red fluorescence because the Myh6-Cre is only expressed in differentiated heart cells. Analysis revealed no red fluorescent cardiomyocytes in RISP-KO hearts and only red fluorescence in the mT/mG-RISP-WT hearts. Flow cytometry analysis of bone marrow from RISP-KO reporter mice revealed only red fluorescent cells, indicating that Myh6-Cre–positive cells are absent in bone marrow (Supplemental Figure 6, B–D). Hence, the new heart cells must have originated from existing cardiomyocytes rather than from migrating progenitors.

### RISP KO–mediated cardiomyocyte proliferation involves mTOR activation but is not caused by decreased ROS signaling or Meis1 levels.

The mitochondrial ETC generates reactive oxygen species (ROS), which could cause oxidative DNA damage and trigger cell cycle arrest (20). RISP KO decreases ROS generation at complex III (21). A decrease in ROS-induced DNA damage might therefore allow cardiomyocytes to return to the cell cycle. To determine the effects of RISP KO on ROS generation, subcellular thiol oxidant status was assessed in neonatal cardiomyocytes from RISP^fl/fl^ creatine kinase–Cre mice using the genetically encoded redox sensor, roGFP, expressed in the cytosol or mitochondrial matrix (22). RISP-KO cells exhibited a significant decrease in mitochondrial thiol oxidation, consistent with the loss of ROS from complex III (Supplemental Figure 7A). By contrast, cytosolic oxidant status was unchanged. To assess the effect of RISP deletion on nuclear DNA oxidation, 8-oxo-deoxyguanine (8-oxo-dG) immunostaining in cardiomyocyte nuclei was assessed in adult RISP-WT and -KO mice at 60 days after tamoxifen (Supplemental Figure 7B). Nuclear 8-oxo-dG staining was decreased in RISP-KO heart cells compared with controls (Supplemental Figure 7C). Does this decrease in DNA damage enable cardiomyocytes to reenter the cell cycle? To test this, oxidant stress was restored in RISP-KO mice by placing them on a diet containing menadione (2-methyl-1,4-naphthoquinone, 120 mg/kg/d), a redox-cycling vitamin K precursor shown previously to induce systemic oxidant stress (23). In other mice, menadione was supplemented with ascorbic acid (120 mg/kg/d) to enhance the redox cycling rate (24); this combination was previously shown to confer therapeutic benefit in a patient with a partial loss-of-function mutation in complex III (25, 26). The menadione diet began at 30 days after tamoxifen administration and continued until day 60, the period during which cardiac remodeling occurs after RISP KO. Hearts were then harvested and assessed for 8-oxo-dG staining in cardiomyocyte nuclei and for cardiac remodeling. The decrease in nuclear oxidant stress in RISP-KO hearts was reversed in the mice treated with menadione (Supplemental Figure 7C). However, significant increases in HW/BW were still observed in the RISP-deficient hearts (Supplemental Figure 7D). Nuclear staining for Ki-67 (Supplemental Figure 7, E and F) and phospho–histone H3 was still indistinguishable between RISP-deficient control-fed and menadione-fed mice at 60 days (Supplemental Figure 7G). Total adenine nucleotide levels decreased in the RISP-deficient hearts, but these were not restored by menadione-ascorbate treatment (Supplemental Figure 7, H and I). Finally, cardiac performance as assessed by echocardiography was not affected by menadione treatment (Supplemental Figure 7, J–L). Collectively, these data indicate that decreased ROS signaling from mitochondria is not responsible for the proliferative response in adult RISP-KO hearts.

Remodeling in RISP-KO hearts is likely driven by a change in transcription of genes regulating growth and differentiation. Previously, the transcription factor Meis1 was shown to increase in neonatal hearts as the cardiomyocytes transitioned to a state of proliferative arrest (27). Moreover, deletion of Meis1 in neonatal mouse cardiomyocytes extended the postnatal proliferative window. However, we did not detect a decrease in the mRNA message for Meis1 or protein expression in the RISP-KO hearts, suggesting that Meis1 is not responsible for the remodeling we observed (Supplemental Figure 8, A and B).

**Figure 6:**
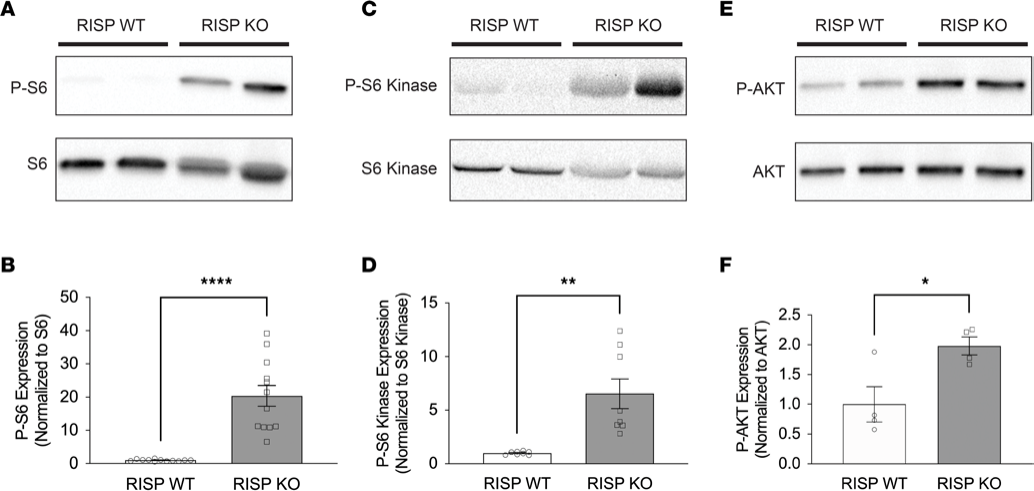
mTOR phosphorylation of protein targets is consistent with the observation of proliferating cardiomyocytes. (**A**) Representative immunoblots of phosphorylated ribosomal S6 and ribosomal S6 from snap-frozen heart homogenates from RISP-WT and -KO mice at 60 days after tamoxifen. (**B**) Band density analysis of phosphorylated ribosomal S6 and ribosomal S6 from RISP-WT and -KO mice at 60 days after tamoxifen; *n* = 12 mice per condition, mean ± SEM, unpaired 2-tailed *t* test. (**C**) Representative immunoblots of phosphorylated S6 kinase and S6 kinase from RISP-WT and -KO mice at 60 days after tamoxifen. (**D**) Band density analysis of phosphorylated S6 kinase and S6 kinase from RISP-WT and -KO mice at 60 days after tamoxifen; *n* = 8 mice per condition, mean ± SEM, unpaired 2-tailed *t* test. (**E**) Representative immunoblots of phosphorylated Akt (p-Akt) and total Akt from RISP-WT and -KO mice at 60 days after tamoxifen. (**F**) Band density analysis of p-Akt and total Akt from RISP-WT and -KO mice at 60 days after tamoxifen; *n* = 4 mice per condition, mean ± SEM, unpaired 2-tailed *t* test. *P < 0.05, **P < 0.01, ****P < 0.0001.

The kinase mTOR is a nutrient sensor that functions as a master regulator of cell growth and proliferation (28). Proliferating cardiomyocytes would be expected to exhibit mTOR activation, as its phosphorylation of protein targets regulates mRNA translation in accordance with amino acid and glucose availability (29). Ribosomal S6 protein is one such target, and RISP-KO hearts at 60 days exhibited robust phosphorylation and activation (Figure 6, A and B). Phosphorylation of S6 is mediated by S6 kinase, which also exhibited significant activation in RISP-KO hearts (Figure 6, C and D). Growth factor signaling leads to the phosphorylation and activation of the phosphatidylinositol-3-kinase Akt, a positive regulator of mTOR. In RISP-KO hearts we observed increased Akt phosphorylation compared with WT (Figure 6, E and F), suggesting that the activation of growth factor signaling contributed to the upregulation of mTOR signaling in RISP-KO hearts. Activation of AMPK in response to bioenergetic deficiency would lead to rapid inhibition of mTORC1 (30), so the increased mTOR activity is consistent with our observed activation of cardiomyocyte proliferation and absence of bioenergetic deficiency, as described in earlier experiments (Figure 1, J and K).

### RISP depletion mediates a transcriptional response consistent with an upregulation of heart developmental processes.

**Figure 7:**
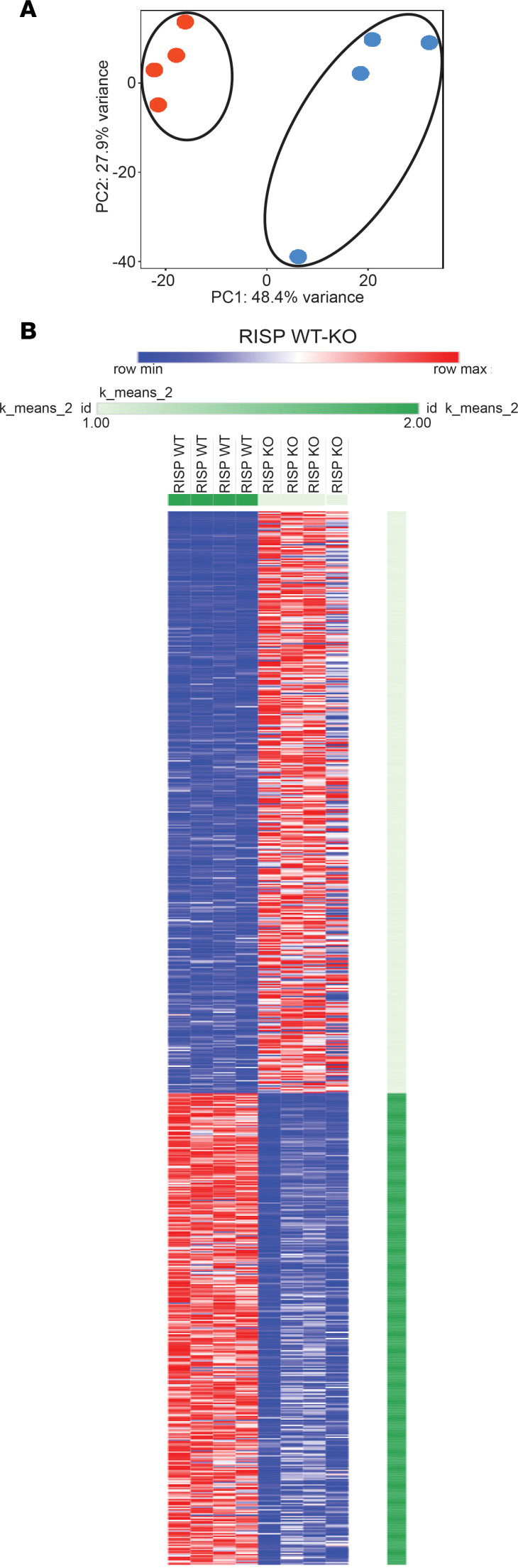
Transcriptomic responses in RISP-WT and -KO hearts at 60 days after tamoxifen. (**A**) Principal component analysis of RNA-Seq data from RISP-WT and -KO hearts. (**B**) *K*-means clustering of gene expression values for 1,355 differentially expressed genes in RISP WT (*n* = 4 mice, left columns) and KO (*n* = 4 mice, right columns) (adjusted *P* < 0.05, edgeR analysis) (red: increased expression relative to blue).

To explore the transcriptional response, RNA sequencing (RNA-Seq) was performed on RNA collected from rapidly frozen tissue harvested from RISP-KO and -WT adult mice. RISP-KO hearts at 60 days revealed an up- or downregulation of 1,355 genes (FDR-adjusted *P* < 0.05) compared with controls (Figure 7, A and B). As expected, expression of *Uqcrfs1* was decreased (adjusted *P* < 1.44 × 10^–39^, log_2_ fold change –4.44). Changes were also detected in genes linked to the mitochondrial unfolded protein response and the amino acid starvation response (Table 1). For example, significant upregulation of activating transcription factors *Atf3*, *Atf4*, and *Atf5*, fibroblast growth factor 21 (*Fgf21*), growth differentiation factor 15 (*Gdf15*), and methylene tetrahydrofolate dehydrogenase, the rate-limiting enzyme in the folate cycle of mitochondria, was noted. In the RISP-KO hearts, Gene Ontology (GO) analysis of biological processes revealed significant upregulation of multiple pathways related to the observed remodeling, including cardiovascular system development, vascular development, cell proliferation, and other developmental processes, consistent with the concept that RISP deletion causes a reawakening of developmental processes (Table 2). Downregulated biological processes were linked to regulation of cardiac muscle contraction, heart contraction, and regulation of heart rate, consistent with the impaired contractile ability in the KO hearts (Table 3).

Consistent with the evidence of mTOR activation, a number of growth factor genes were significantly altered as well, with insulin-like growth factor 1 (*Igf1*), heparin-binding EGF-like growth factor (*Hbegr*), and connective tissue growth factor (*Ctgf*) showing significant increases (Table 4). No increase in *Vegfa*, *Vegfb*, or *Vegfc* was detected, although, interestingly, the remodeling was associated with an increase in capillary density (Supplemental Figure 3G). These results reveal that RISP KO leads to a transcriptional response consistent with an upregulation of developmental processes in the heart, which aligns with the observed hyperplastic phenotype.

**Figure 8:**
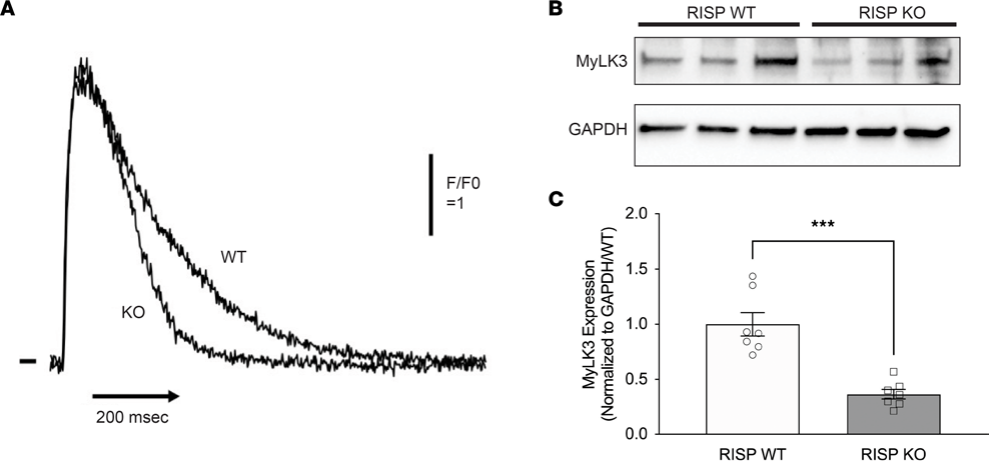
Decline in cardiac function because of decreased expression of proteins sustaining contractility. (**A**) Ca^2+^ measurements in paced cardiomyocytes revealed that the initial rise in Ca^2+^ was indistinguishable between RISP-WT and -KO mice. However, sequestration of Ca^2+^ was faster in the KO cardiomyocytes compared with WT. Further analysis of Ca^2+^ dynamics is presented in Supplemental Figure 9, A–H. (**B**) Immunoblotting for MyLK3 protein revealed a decrease in RISP-KO compared with WT hearts at 60 days after tamoxifen. (**C**) Band density analysis of immunoblots of MyLK3 and GAPDH in RISP-WT and -KO hearts at 60 days after tamoxifen; *n* = 7 mice per condition, mean ± SEM, unpaired 2-tailed *t* test. ***P < 0.001.

To clarify the mechanism responsible for the decline in cardiac function described earlier (Figure 4), we assessed Ca^2+^ activation in freshly isolated electrically paced adult cardiac myocytes from RISP-KO and -WT hearts loaded with Rhod-4AM at 60 days after tamoxifen. No differences were detected in the rising phase of activation, or in the peak levels of Ca^2+^ (Figure 8A and Supplemental Figure 9, A–H). However, the rate of decline in Ca^2+^ was significantly faster in the RISP-KO cells compared with controls. The faster sequestration of Ca^2+^ is characteristic of younger hearts, consistent with the proposed upregulation of developmental processes in the RISP-deficient hearts. Moreover, as Ca^2+^ uptake is an energy-dependent process, this response is consistent with the absence of bioenergetic stress.

The observation that calcium activation was normal in RISP-KO cells indicated that the cardiac functional defect must be downstream from Ca^2+^ signaling. The transcriptional analysis had identified downregulation of gene sets linked to regulation of cardiac muscle contraction. Notably, expression of myosin light chain kinase 3 (*Mylk3*), which is critical for sustaining contractile function, was significantly downregulated at the mRNA level. Western blot analysis verified that protein expression was also decreased in the RISP-KO hearts (Figure 8, B and C). These findings suggest that decreases in expression of proteins sustaining cardiac contractility likely contributed to the decline in cardiac function, although other factors downstream from calcium signaling could also have contributed.

Although Hippo-YAP/TAZ signaling has been implicated in organ regeneration (31), Western blotting of phospho-YAP relative to total YAP protein demonstrated no difference between WT and RISP-KO hearts (data not shown).

### RISP KO results in alterations of metabolic substrate levels that alter DNA methylation and gene expression.

**Figure 9:**
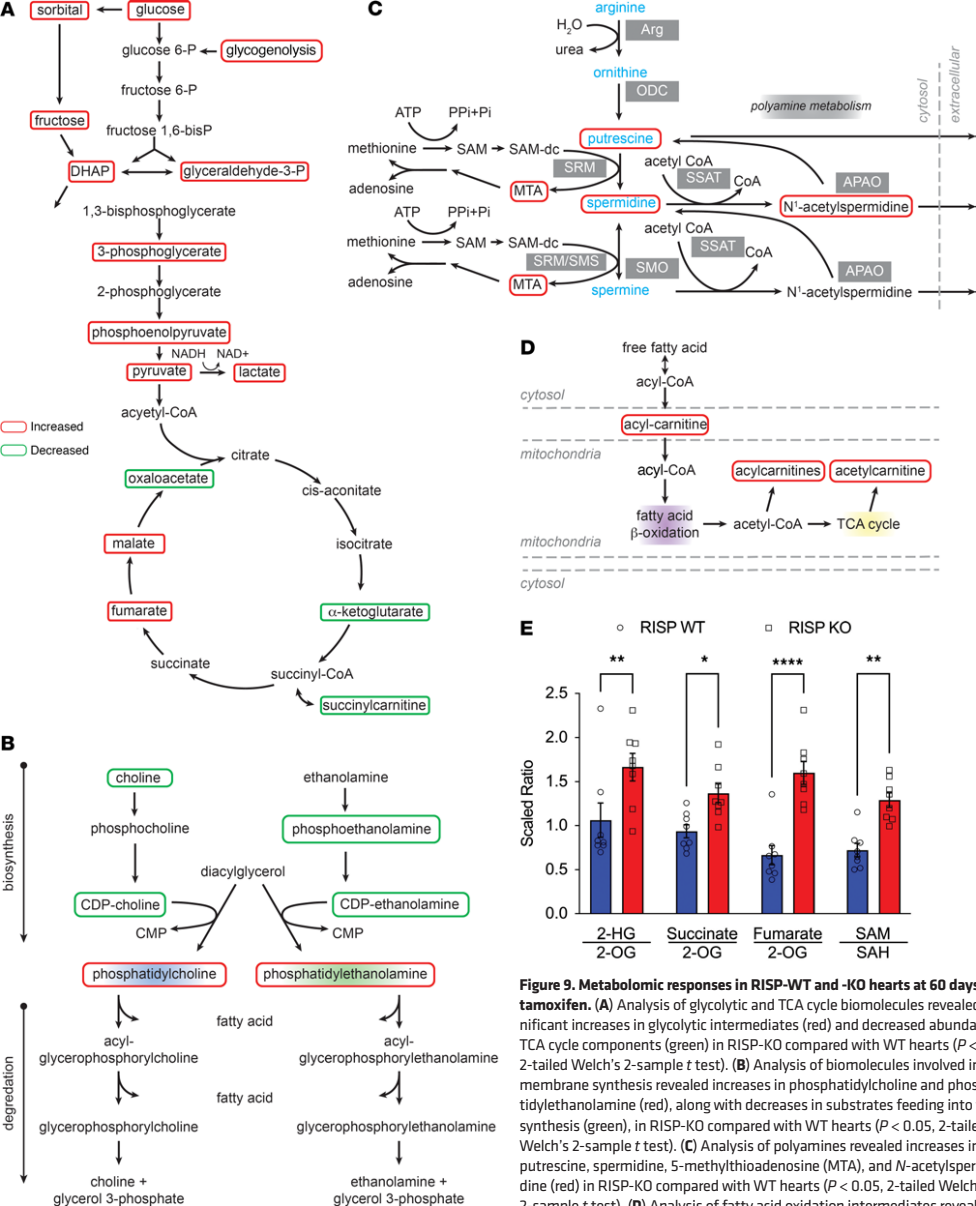
Metabolomic responses in RISP-WT and -KO hearts at 60 days after tamoxifen. (**A**) Analysis of glycolytic and TCA cycle biomolecules revealed significant increases in glycolytic intermediates (red) and decreased abundance of TCA cycle components (green) in RISP-KO compared with WT hearts (*P* < 0.05, 2-tailed Welch’s 2-sample *t* test). (**B**) Analysis of biomolecules involved in membrane synthesis revealed increases in phosphatidylcholine and phosphatidylethanolamine (red), along with decreases in substrates feeding into their synthesis (green), in RISP-KO compared with WT hearts (*P* < 0.05, 2-tailed Welch’s 2-sample *t* test). (**C**) Analysis of polyamines revealed increases in putrescine, spermidine, 5-methylthioadenosine (MTA), and *N*-acetylspermidine (red) in RISP-KO compared with WT hearts (*P* < 0.05, 2-tailed Welch’s 2-sample *t* test). (**D**) Analysis of fatty acid oxidation intermediates revealed increases in acyl-carnitines (red) in RISP-KO compared with WT hearts (*P* < 0.05, 2-tailed Welch’s 2-sample *t* test). (**E**) Scaled ratio of biochemical factors that promote DNA methylation in RISP-WT (blue) and -KO hearts (red); *n* = 8 mice per condition, mean ± SEM, unpaired 2-tailed *t* test. *P < 0.05, **P < 0.01, ****P < 0.0001.

Why would loss of complex III trigger a transcriptomic response leading to cellular hyperplasia? As mitochondria are critical for diverse metabolic functions in the cell, one possibility is that progressive ETC loss alters cellular metabolite levels that contribute to growth, cell proliferation, and the regulation of gene transcription. To explore this, hearts of anesthetized, mechanically ventilated RISP-KO and -WT control mice were snap-frozen in situ and analyzed for metabolite levels by liquid chromatography–mass spectrometry of 695 known biochemicals. A total of 298 biochemicals were significantly altered (190 increased, 108 decreased) in the RISP-KO hearts. Principal component analysis detected significant distinction between WT and RISP-KO hearts (Supplemental Figure 10A). Random forest classification revealed key differences in lipid, amino acid, and carbohydrate metabolism in comparison with WT hearts (Supplemental Figure 10B). Consistent with the loss of ETC function, significant increases in glycolytic intermediates were detected, including glucose, glucose 1,6-diphosphate, fructose 1,6-diphosphate, dihydroxyacetone phosphate, 3-phosphoglycerate, phosphoenolpyruvate, pyruvate, and lactate (Figure 9A). In the pentose phosphate pathway, 6-phosphogluconate was increased, though ribose 1-phosphate was decreased. Similarly, fructose, mannitol/sorbitol, and mannose were significantly elevated in RISP-KO hearts compared with controls. By contrast, components of the Krebs cycle were significantly diminished, including oxaloacetate, citrate, aconitase, α-ketoglutarate, and succinate. Fumarate and malate were significantly increased. Membrane remodeling pathways were also affected, with decreases in choline, CDP-choline, and CDP-ethanolamine, but increases in phosphatidylcholine and phosphatidylethanolamine (Figure 9B). Increases in polyamine synthesis have been linked to increases in proliferation, while decreases have been noted in senescent cells (32). In RISP-deficient hearts, polyamine metabolism was significantly affected, with increases in putrescine, spermidine, *N*^1^-acetylspermidine, and 5-methylthioadenosine (Figure 9C). Finally, increases were detected in fatty acid metabolism intermediates and acyl-carnitine, along with hexanoyl, palmitoyl, linoleoyl, oleoyl, myristoleoyl, and arachidoyl carnitines (Figure 9D), which may represent a backup of mitochondrial fatty acid β-oxidation caused by limitations in the ETC. These findings reveal that RISP KO causes a shift in metabolism that leads to alterations in metabolite levels, which could conceivably drive the transcriptional response.

How could altered metabolite levels affect transcription? Gene expression is affected by chromatin structure, and DNA methylation at cytosines of the nucleotide sequence CpG is a key mechanism in the epigenetic regulation of gene expression. One important mitochondrial task is to supply substrates used by enzymes involved in DNA methylation/demethylation. For example, α-ketoglutarate, a Krebs cycle intermediate, is a required substrate for DNA demethylation by the TET family of dioxygenases, while *S*-adenosylmethionine (SAM) is the methyl donor for DNA methyltransferases (33). Alterations in the availability of these substrates after RISP deletion could therefore alter epigenetic regulation of transcription. Inhibition of the ETC can also increase the production of l-2-hydroxyglutarate (2-HG), a competitive inhibitor of demethylases, via promiscuous reduction of α-ketoglutarate by malate dehydrogenase or lactate dehydrogenase (34, 35). Moreover, in the absence of complex III function, the conversion of 2-HG back to α-ketoglutarate by 2-HG dehydrogenase is inhibited (12, 13). Succinate and fumarate can also inhibit demethylase activities (6). To assess metabolite levels affecting methylation/demethylation, the ratios of 2-HG/α-ketoglutarate, succinate/α-ketoglutarate, and fumarate/α-ketoglutarate were compared in RISP-deficient and control hearts (Figure 9E). These ratios increased, as did the ratio of SAM to S-adenosylhomocysteine (SAH), favoring an increase in methylation. Thus, the observed changes in metabolite levels could conceivably alter DNA methylation in the RISP-KO mice, thereby inducing transcriptional changes that drive developmental processes and cell proliferation.

**Figure 10:**
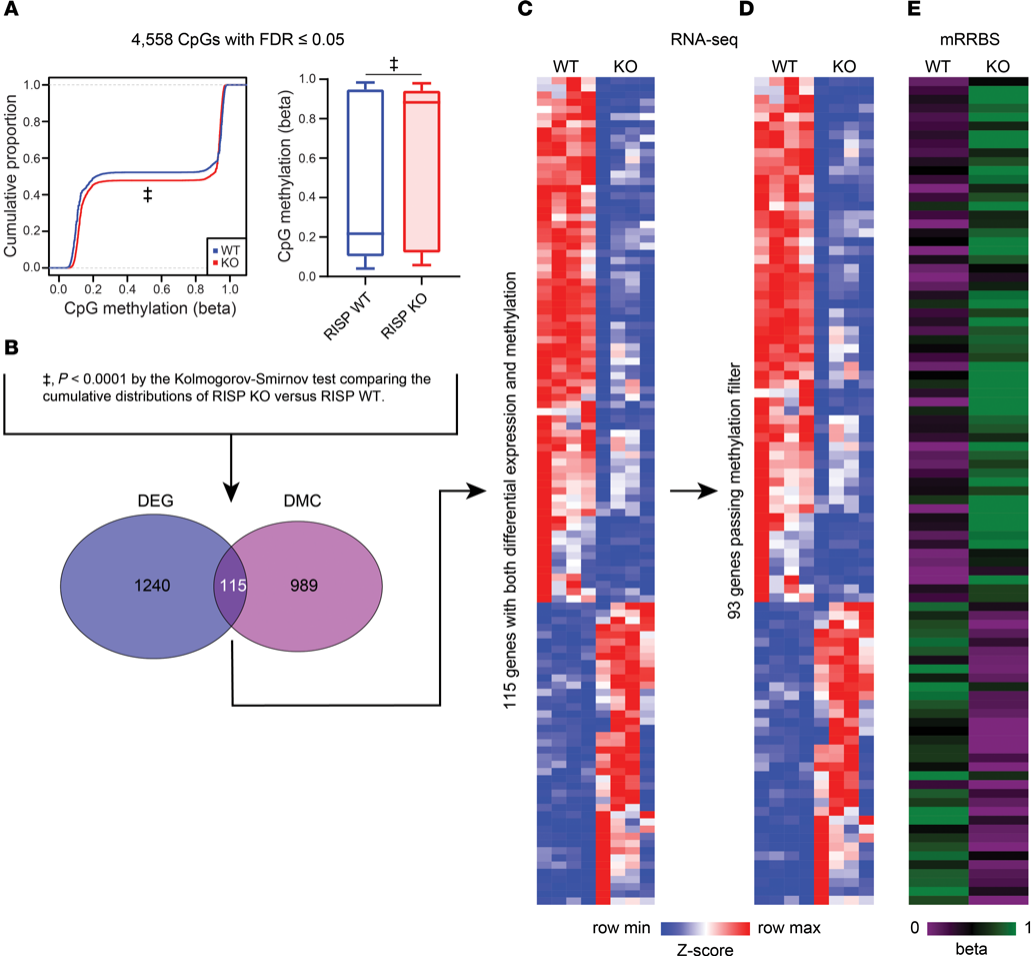
Epigenetic analysis of RISP-WT and -KO hearts. (**A**) Left: Cumulative distribution of DNA CpG methylation in RISP-WT (blue) and -KO (red) hearts. Right: Box-and-whisker plot showing significant increase in DNA CpG methylation (DMC) in RISP-KO compared with WT hearts (*P* < 0.0001, Kolmogorov-Smirnov test). Box plots show the interquartile range, median (line), and minimum and maximum (whiskers). (**B**) Venn diagram comparing differentially expressed genes (DEGs) and differentially methylated DNA CpG sites located in regulatory regions of genes reveals an overlap of 115 genes. (**C**) *K*-means clustering of RNA-Seq identification of 115 DEG and DMC genes showing directional change (upregulated, red, versus downregulated, blue) in RISP-KO (*n* = 4) versus WT hearts (*n* = 4). (**D**) *K*-means clustering after filtering was applied to restrict the data set to CpGs with 25% higher methylation in lower expression groups compared with higher expression groups. This identified 93 DEG and DMC genes in RISP-KO (*n* = 4) versus WT hearts. (**E**) DNA CpG methylation status of 93 DEGs and DMCs showing that downregulated genes are more highly methylated (green) in regulatory regions, while upregulated genes are less highly methylated (violet), in RISP KO compared with WT. For **C**–**E**, DEGs were determined by a generalized linear model and ANOVA-like testing with FDR *q* value less than 0.05, and DMCs were determined by a β-binomial regression model with an arcsine link function fitted using the generalized least-squares method and Wald test FDR *q* value less than 0.05.

To determine the effects of RISP KO on CpG methylation, DNA methylation profiling was assessed using a modified reduced-representation bisulfite sequencing (mRRBS) method to identify the CpG methylation landscape responses in snap-frozen hearts (36–40). This identified 4,558 differentially methylated CpG sites (FDR ≤ 0.05) with a significant global increase in DNA methylation across the genome (Figure 10A). Of these, 1,104 differentially methylated CpGs were most likely to affect translation, as they were located within 2 kb of the transcriptional start sites of genes (Figure 10B). Comparison of genes that were both differentially expressed (DEG) and differentially methylated (DNA CpG methylation: DMC) revealed 115 common elements. This overlapping gene set was linked to relevant upregulated pathways related to the observed RISP-KO heart phenotype, including cardiac muscle tissue development, heart development, muscle cell development, muscle structure development, myofibril assembly, striated muscle cell differentiation, and sarcomere organization (Table 5). In addition, significant downregulation of other processes was identified in the GO analysis of overlapping genes (Table 6), some of which were linked to regulation of cardiac muscle contraction, heart contraction, and regulation of heart rate, again consistent with the impaired contractile ability in the KO hearts. *K*-means clustering of these genes revealed a data structure similar to the *k*-means clustering of all DEGs in Figure 7B (Figure 10C). Of these 115 genes, 93 passed a stringent DNA methylation filter designed to detect genes with promoters bearing CpG methylation that is anticorrelated with their expression (36) (Figure 10D). A comparison between the change in expression (increased vs. decreased) and methylation status revealed that the majority of suppressed genes had undergone an increase in methylation, whereas those genes showing an increased expression in the RISP-KO hearts demonstrated a decrease in methylation (Figure 10E). Why did methylation decrease for certain genes when overall CpG methylation increased? Most likely, the increased methylation caused by metabolomic changes altered the expression of genes having secondary effects on methylation, resulting in a mixture of genes with increased or decreased methylation and decreased or increased expression, respectively.

Pathway analysis revealed that these differentially expressed and methylated genes were associated with upregulated developmental processes including muscle structure development, cardiac muscle development, and cell development. The specific genes that were differentially methylated and differentially expressed (Table 7) yet have not previously been linked to cell proliferation. These findings reveal that RISP KO alters the availability of substrates affecting DNA methylation, resulting in changes in methylation of CpGs in the promoter regions of genes broadly linked to cardiovascular development. Moreover, changes in the expression of these genes corresponded to the changes in the methylation status of their promoters.

To address the possibility that Myh6-Cre activation in WT mice might affect cardiac remodeling or function, we compared HW/BW and cardiac function in Cre-negative and Cre-positive RISP^+/+^ mice at 60 days after tamoxifen (Supplemental Figure 12, A–F). No differences were observed, indicating that Cre activation by itself does not induce cardiac remodeling or changes in cardiac function.

## Discussion

Genetic deletion of *Uqcrfs1* in adult cardiomyocytes leads to a progressive loss of RISP protein in the heart. At the point when partial inhibition of mitochondrial function has developed, these hearts demonstrate an upregulation of glycolysis, as indicated by FDG-PET scanning and increased tissue lactate and pyruvate levels. As the decline in ETC function progresses, the hearts undergo profound hyperplastic remodeling and develop a progressive cardiomegaly, as evidenced by increased nuclear staining for Ki-67 and phospho–histone H3, increases in EdU staining in vivo, a doubling of the number of cardiomyocytes by direct counting of digested hearts, and a parallel increase in the total number of cardiomyocyte nuclei. The loss of RISP is associated with an upregulation of transcriptional pathways associated with heart and cardiovascular development as well as cell proliferation, but a downregulation of pathways associated with contractile function. Collectively, these findings are consistent with the conclusion that RISP deletion in the heart induces dedifferentiation toward a perinatal developmental state, with increased glycolysis and a restoration of mitotic capacity but a decrease in contractile function.

A bioenergetic crisis was not induced by RISP KO, as evidenced by (a) lack of a decrease in cellular energy charge, (b) absence of AMPK activation, (c) evidence of mTOR and Akt activation that is consistent with the proliferative response but inconsistent with a bioenergetic deficiency (29), and (d) sustained peak Ca^2+^ activation, and accelerated Ca^2+^ sequestration resembling the behavior of younger heart cells. The data also suggest that shifts in cardiac metabolism arising from RISP depletion led to increases in metabolite levels that promote DNA methylation and decreases in α-ketoglutarate needed for demethylation, resulting in increased in CpG methylation within regulatory regions of genes that promote cardiac development. Thus, by regulating cellular metabolism affecting epigenetic control, mitochondria regulate developmental state and mitotic capacity in cardiomyocytes. It is conceivable that the normal developmental progression from fetal glycolytic metabolism toward the mitochondrial OXPHOS in the postnatal heart contributes to the loss of proliferative capacity that persists through adulthood.

Mitochondrial cardiomyopathies arising from complex III dysfunction have been linked to hypertrophic remodeling, which can progress to decompensation and dilation of the LV (41). However, we detected no similarities to cardiac hypertrophy or dilated cardiomyopathy in terms of the DEGs. In that regard, a comparison between a set of 35 DEGs identified in hypertrophic hearts (42) and our top 275 DEGs identified zero overlap. We also compared our top 275 DEGs with the top 50 genes distilled from multiple RNA-Seq studies of dilated cardiomyopathy (43). This, too, revealed no overlap. Thus, the gene signatures of the RISP-KO hearts do not resemble those of hypertrophic or dilated cardiomyopathy.

The cellular morphology and cardiac structure also failed to resemble a classical hypertrophic or dilated cardiomyopathy. In hypertrophic hearts an increase in the lateral cell dimension would be expected, but RISP-KO cardiomyocyte dimensions at 60 days were indistinguishable from controls. Dilated cardiomyopathies are characterized by increased LV volume, a thinned LV wall, and likely a decrease in cell width. The RISP-deleted hearts did have increased LV volumes, but the LV wall was only slightly thickened, the widths of the cells were unaffected, and the total number of cardiomyocytes had doubled. This picture is consistent with end-to-end cell division and subsequent growth leading to a large increase in LV circumference without a change in the number of cells across the LV wall or a change in cell dimensions (shown schematically in Figure 3G). In that sense, comparisons between the RISP-KO phenotype and a classical dilated heart should be made with caution.

The increase in HW/BW was associated with a small but significant increase in posterior wall thickness (PWT) and an increase in LV end-diastolic volume (LVEDV) as assessed by echocardiography (Supplemental Figure 11, A and B). However, there was no change in LVEDV/PWT (Supplemental Figure 11C) and no increase in LVEDV/HW (Supplemental Figure 11D), as would be expected for a dilated heart. Hence, the increased size of the LV is not out of proportion to the larger heart weight — these are larger hearts by virtue of having twice the number of cardiomyocytes versus controls. The larger LV chambers are not disproportionate to the increased heart size. Collectively, these data indicate that the remodeling produces a larger heart with chamber sizes that are consistent with the larger heart mass. Hence, the RISP hearts resemble neither a dilated cardiomyopathy nor hypertrophic remodeling. The ratio of binuclear/mononuclear cells decreased during remodeling, so some of the new cardiomyocytes were the product of binuclear cells dividing into mononuclear, a process that would naturally slow as the binuclear pool of cardiomyocytes decreased. However, the total number of cardiomyocyte nuclei increased in parallel with the number of cardiomyocytes, so de novo cardiomyocyte synthesis also contributed. To our knowledge this remodeling process has not been described previously.

If a bioenergetic deficiency did not exist, why was contractile function impaired? Studies of peak Ca^2+^ activation in paced cells revealed no differences between RISP-KO and control cardiomyocytes, suggesting that the mechanism must lie downstream from calcium activation. The transcriptional GO analysis revealed a downregulation of genes linked to contractile function, including *Mylk3*, which phosphorylates myosin light and heavy chains, thereby potentiating the force and rate of cross-bridge recruitment in cardiac myocytes (44). Interestingly, this gene was also among the cohort of differentially methylated and differentially expressed genes. Western blotting revealed that its protein expression at 60 days was suppressed, consistent with the idea that these cells have “walked back” developmentally toward a perinatal stage that lacks the contractile activity normally seen in adult cardiomyocytes.

Expression of multiple growth factor genes was increased after RISP KO (Table 1), which likely contributed to the increased Akt phosphorylation and mTOR activation in 60-day hearts. The role of mTOR in the regulation of aerobic glycolysis is complex and not fully understood (45). However, it is conceivable that the increase in glycolytic flux we observed, in the absence of transcriptional activation of glycolytic genes, could have been facilitated by mTOR through its ability to upregulate glucose transporters to the plasma membrane of cardiomyocytes (46).

Previous reports have linked specific genes to the cell cycle arrest in cardiomyocytes. Meis1 was reported to control cell cycle arrest in hearts of newborn mice, and genetic deletion of Meis1 and its cofactor Hoxb13 was shown to activate mitosis in adult mouse hearts (27, 47). However, we found no changes in message or protein levels of Meis1 in the RISP-KO hearts, indicating that this transcription factor was not involved in the remodeling we observed. In another study, myocardial deletion of pyruvate dehydrogenase kinase 4 led to decreases in fatty acid oxidation and increases in oxidation of pyruvate by mitochondria (48). Our results are not inconsistent with those findings, but unlike in our study, the extent of cardiomyocyte proliferation in that model did not produce an increase in HW/BW. Finally, we found no evidence of Hippo-YAP/TAZ activation in the RISP-KO hearts.

After myocardial ischemia in RISP-KO hearts, projections of live cardiomyocytes can be seen invading the infarcted region at day 60. By contrast, control hearts show well-defined infarct borders without evidence of live cardiomyocytes within the injured area. These findings therefore reveal the potential for repair of injured hearts in the future. However, the progressive loss of RISP also causes a decline in cardiac function, which becomes severe by 75 days and eventually leads to lethal cardiac failure. Future studies are required to determine whether a restoration of *Uqcrfs1* in the injured heart could restore RISP levels and mitochondrial function, reversing the contractile deficits and allowing the newly formed cardiomyocytes in the infarct region to contribute to a renewal of contractile function.

Malonate, a competitive inhibitor of complex II, was reported to extend the proliferative window in neonatal cardiomyocytes and stimulate cardiomyocyte cell division in adult mouse hearts after the induction of myocardial infarction (49). Interesting similarities between that study and ours exist, although important differences are also evident. Both studies induced a partial inhibition of mitochondrial function; Bae et al. administered an inhibitor of complex II, and ours involved genetic disruption of complex III. Both studies observed metabolomic changes consistent with a shift toward glycolysis, as expected based on the inhibitory effects on mitochondrial function. Unlike Bae et al., we found an increase in glucose utilization and lactate accumulation, but perhaps that reflects a more severe ETC inhibition with RISP deletion. Both studies observed that newly formed cardiomyocytes are capable of repopulating myocardial areas damaged by ischemia. However, one notable difference is that malonate only increased adult cardiomyocyte proliferation in the context of infarction, whereas RISP deletion caused a profound proliferative response throughout the heart, in both ischemia-injured and in noninjured conditions. Given that malonate should affect myocyte mitochondria similarly throughout the heart, the mechanism underlying the localized proliferative response is not clear. Another difference relates to the interpretation regarding the role of ROS and oxidative DNA damage. RISP deletion attenuated oxidative stress in the hearts, and malonate would also be expected to decrease ROS generation by the ETC. We used menadione to restore oxidant stress in the hearts, which it did without blunting the hyperplastic remodeling, leading us to conclude that decreases in oxidant stress were not responsible for the cardiomyocyte proliferation caused by RISP KO. That observation, combined with the metabolomic effects that favored developmental pathways driven by DNA methylation in the RISP-KO hearts, led us to conclude that the changes in metabolites caused by mitochondrial inhibition were responsible for driving the remodeling. That conclusion is consistent with the one Bae et al. reached.

## Methods

Detailed methods can be found in Supplemental Methods.

### Sex as a biological variable.

All mouse studies included an equal number of male and female animals, and similar findings are reported for both sexes.

### Animal studies.

Mice were housed under specific pathogen–free conditions in ventilated cage racks in the Northwestern University animal facility under 12-hour light/12-hour dark cycling, and were fed with standard laboratory chow, ad libitum, except where otherwise specified.

Myh6-Cre was activated using tamoxifen (0.5 mg, i.p. for 5 sequential days), after which hearts were studied at 30, 60, and 75 days.

### Statistics.

Detailed statistical methodology can be found in Supplemental Methods. The following statistical tests were used and are denoted in the figure legends: an unpaired, 2-tailed *t* test; a 2-way ANOVA with a Holm-Šídák multiple-comparison test (2W-ANOVA-Šidák’s); a 2-tailed Welch’s 2-sample *t* test; an edgeR analysis (50); a Kolmogorov-Smirnov test; a generalized linear model and ANOVA-like test; and a β-binomial regression model with an arcsine link function fitted using the generalized least-squares method and Wald test. To control for experimental differences in the responses, experimental studies and control studies were always carried out on the same day. Statistical significance was set at *P* less than 0.05.

### Study approval.

All animal work was performed according to protocols approved by the Northwestern University Institutional Animal Care and Use Committee.

### Data availability.

Gene expression data were deposited in the NCBI’s Gene Expression Omnibus database (accession GSE264439). A Supporting Data Values file is available online as supplemental material.

## Author contributions

GBW, PTM, BDS, SJS, JAW, AVM, NSC, EBT, and PTS designed research studies. GBW, KAS, PTM, VJD, KAH, CBP, JB, SJS, GO, JAW, DD, SZ, YT, AM, EBT, and PTS conducted experiments. GBW, KAS, PTM, VJD, KAH, BDS, CBP, JB, LN, SJS, GO, JAW, AVM, GRSB, HAV, DD, SZ, EBT, and PTS acquired data. GBW, KAS, PTM, VJD, KAH, BDS, CBP, LN, SJS, GO, JAW, WAM, AVM, GRSG, HAV, DD, EB, SZ, EBT, and PTS analyzed data. KAH, BDS, CBP, JB, JAW, GRSB, HAV, YT, AM, and HA provided reagents. GBW, BDS, SJS, NSC, EBT, and PTS wrote the manuscript.

## Supplementary Material

Supplemental data

Unedited blot and gel images

Supporting data values

## Figures and Tables

**Table 7:**
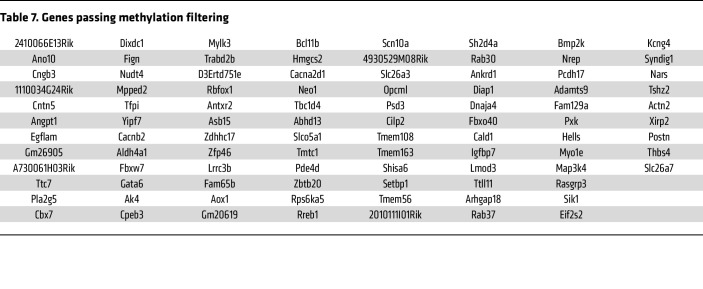
Genes passing methylation filtering

**Table 6:**
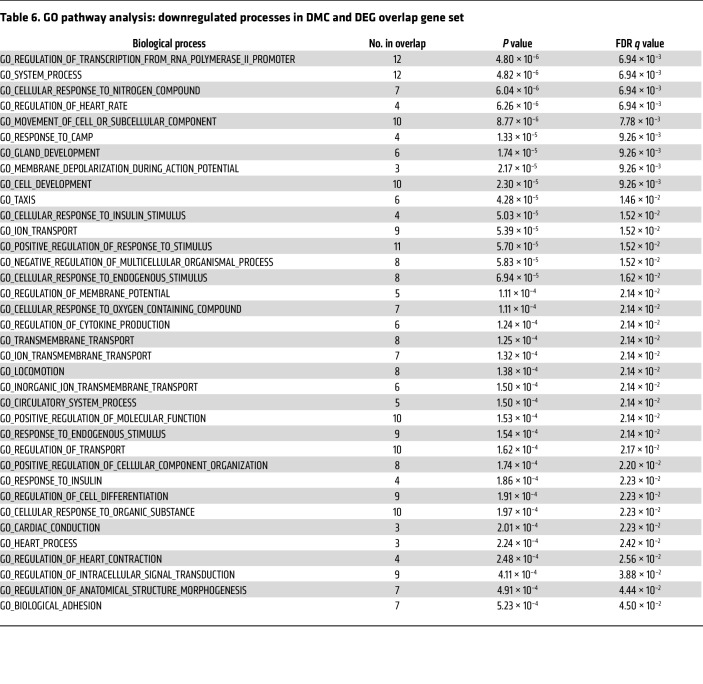
GO pathway analysis: downregulated processes in DMC and DEG overlap gene set

**Table 5:**
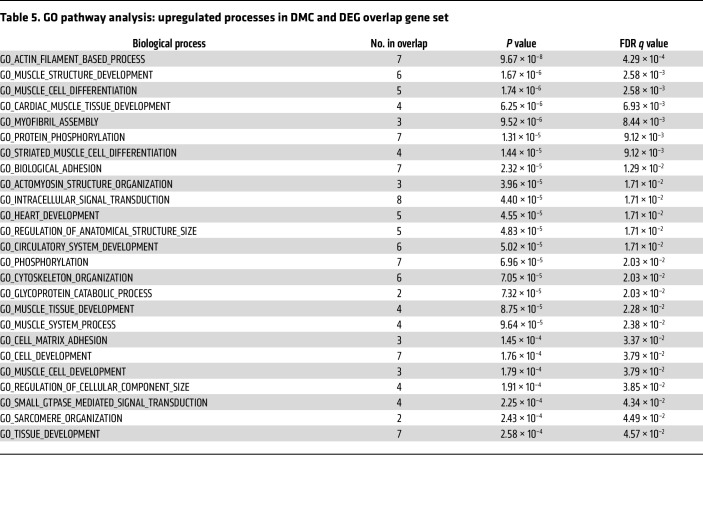
GO pathway analysis: upregulated processes in DMC and DEG overlap gene set

**Table 1:**
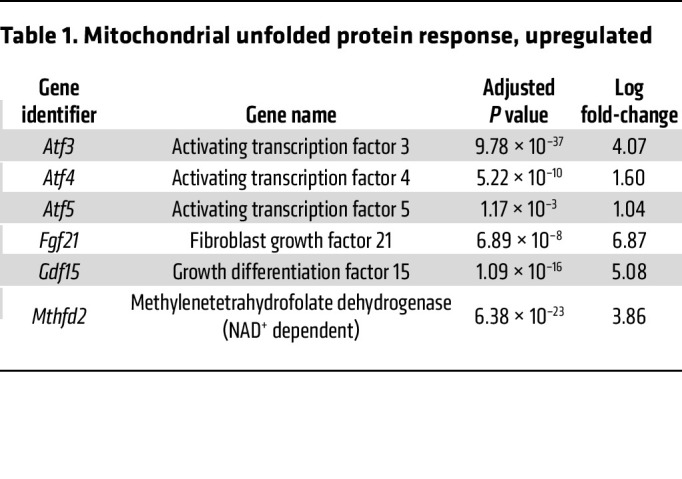
Mitochondrial unfolded protein response, upregulated

**Table 2:**
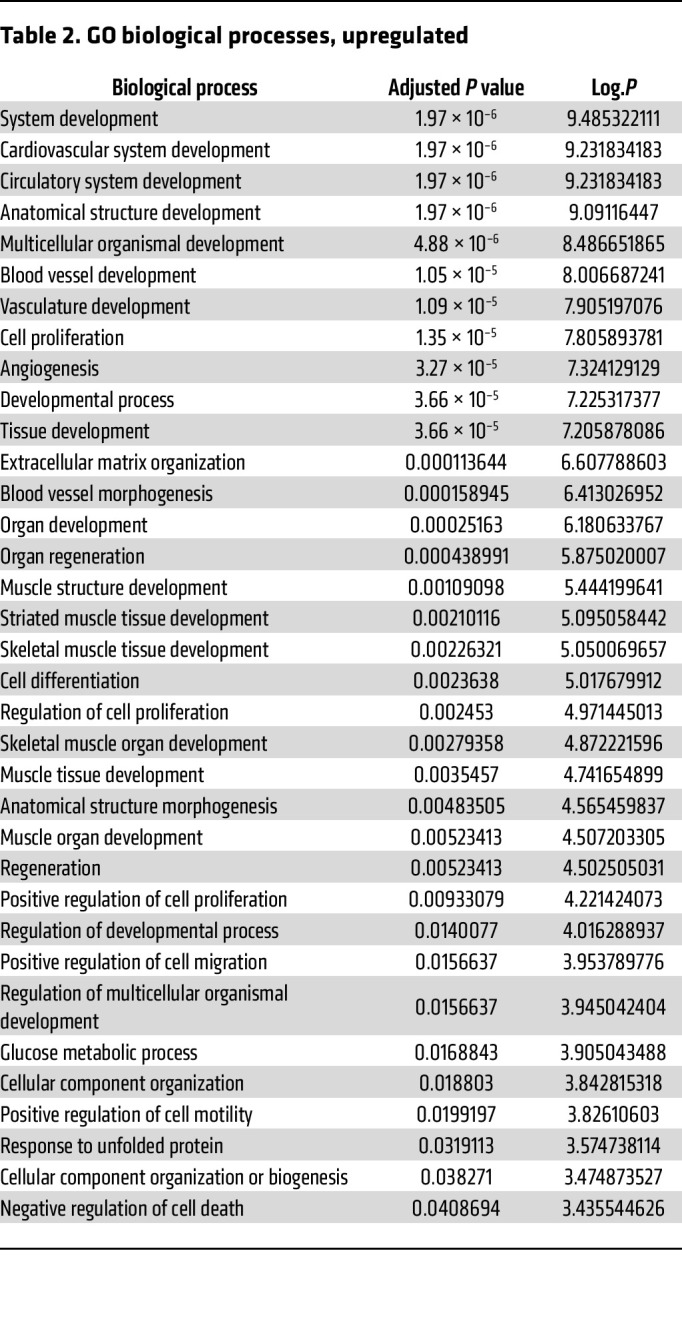
GO biological processes, upregulated

**Table 3:**
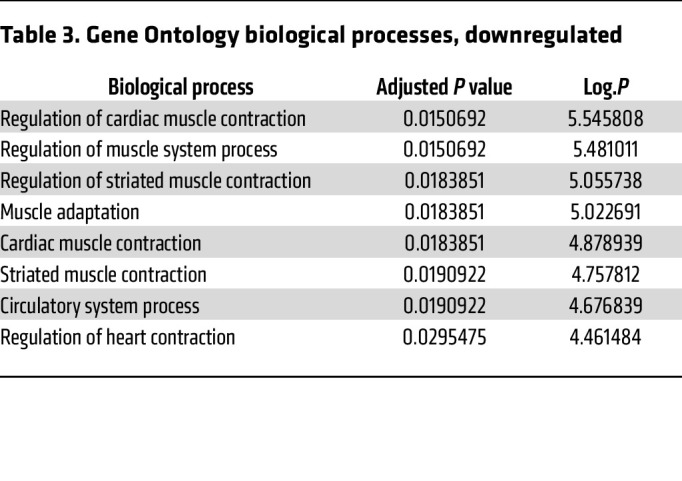
Gene Ontology biological processes, downregulated

**Table 4:**
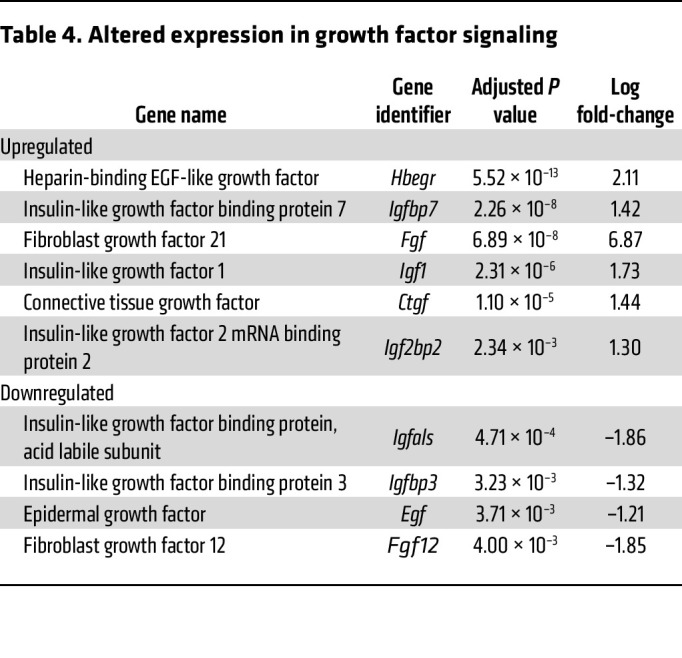
Altered expression in growth factor signaling

## Version 1 05/09/2024

In-Press Preview

## Version 2 07/01/2024

Electronic publication
