# Supplementary material for: Mitochondria regulate proliferation in adult cardiac myocytes
Source: J Clin Invest. 2024 May 9;134(13):e165482. doi: 10.1172/JCI165482 (PMC11213516; doi:10.1172/JCI165482)
Supplement: Supplemental data [file jci-134-165482-s055.pdf]

Supplementary Figures

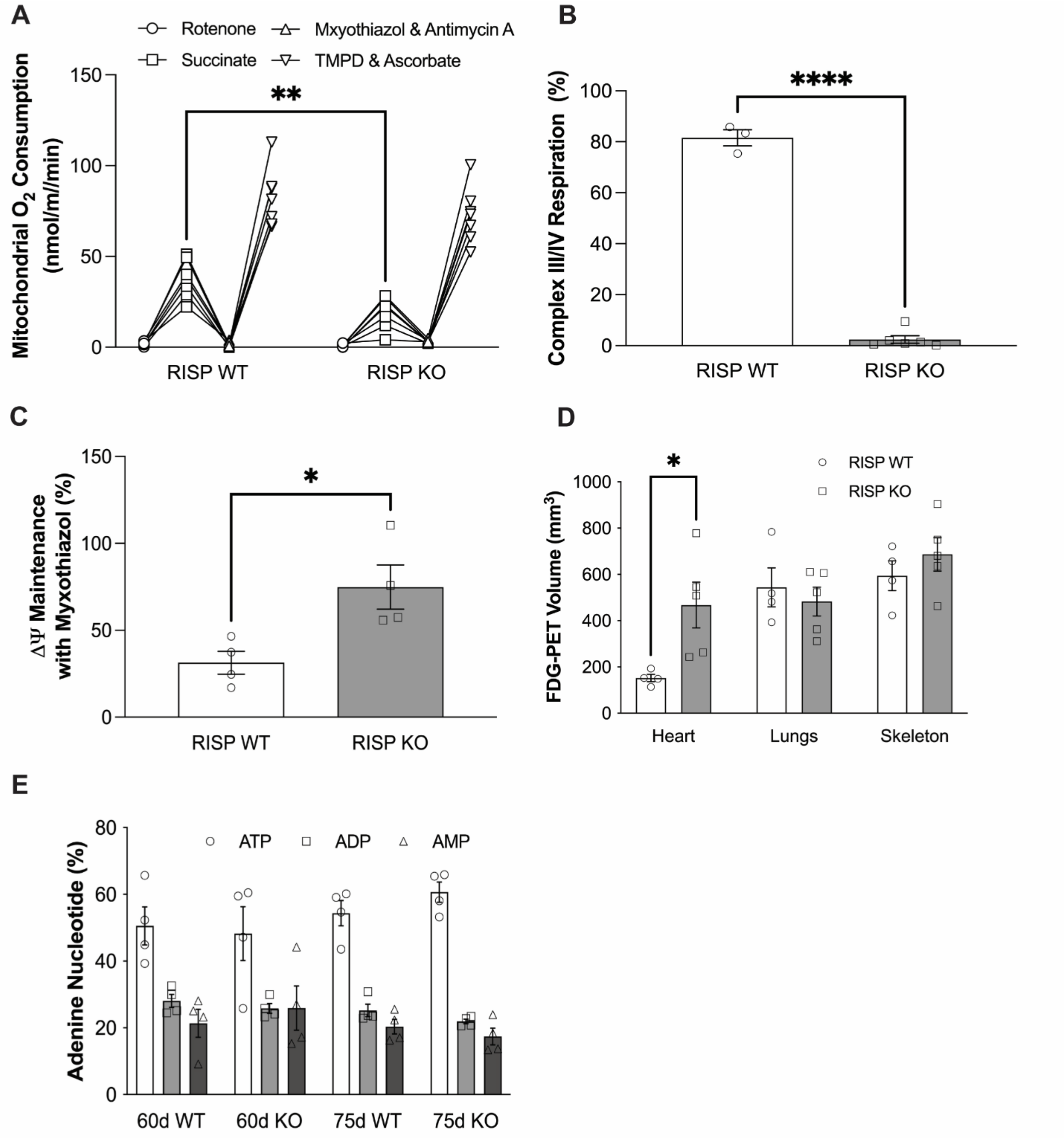

Supplemental Figure S1

Supplementary Figure S1. (**A**) Respiration by mitochondria isolated form RISP WT and RISP KO hearts at 60 days post tamoxifen. When treated with rotenone electron flux through the mitochondrial electron transport chain is blocked. When oxidized with succinate (complex III respiration, state 3), mitochondrial respiration is decreased in the RISP KO mitochondria compared to the RISP WT. Respiration is again blocked when treated with mxyothiazol and antimycin A. Mitochondrial respiration is returned to normal in both RISP WT and KO mitochondria with TMPD and ascorbate (complex IV respiration), ANOVA-Tukey's. (**B**) Respiration by mitochondria isolated from RISP WT and KO hearts at 75 days. When oxidizing succinate/pyruvate, WT mitochondria respired at ~80% of the rate seen when electrons were delivered to cytochrome c using TMPD and ascorbate. By contrast, succinate/pyruvate-driven respiration in RISP KO mitochondria was virtually absent, whereas respiration with TMPD and ascorbate was normal, n=3-6 mice per condition, mean±SEM, U-2t-T-test. (**C**) Mitochondrial membrane potential in neonatal mouse cardiomyocytes from $RISP^{fl/fl}$-creatine kinase Cre mice (RISP KO) or wild type littermate controls (RISP WT), loaded with TMRM under non-quenching conditions. Myxothiazol (2 μm) elicited a significant ($p<0.05$) decrease in membrane potential in WT cells, indicating a reliance on complex III activity, compared to RISP KO cells that rely on glycolysis, n=4-5 mice per condition, mean±SEM, U-2t-T-test. (**D**) Volumes for tissues marked as regions of interest as measured by positron emission tomography of ^{18}F-fluordeoxyglucose utilization (FDG-PET), n=4-5 mice per condition, mean±SEM, U-2t-T-test. (**E**) Adenine nucleotide levels (expressed as percent of the total adenine nucleotide pool) in snap-frozen heart tissue from RISP WT and RISP KO mice at 60 and 75 days after tamoxifen administration, n=4 mice per condition, mean±SEM, 2W-ANOVA-Sidak's. For all graphs *p<0.05, **p<0.01, ****p<0.0001.

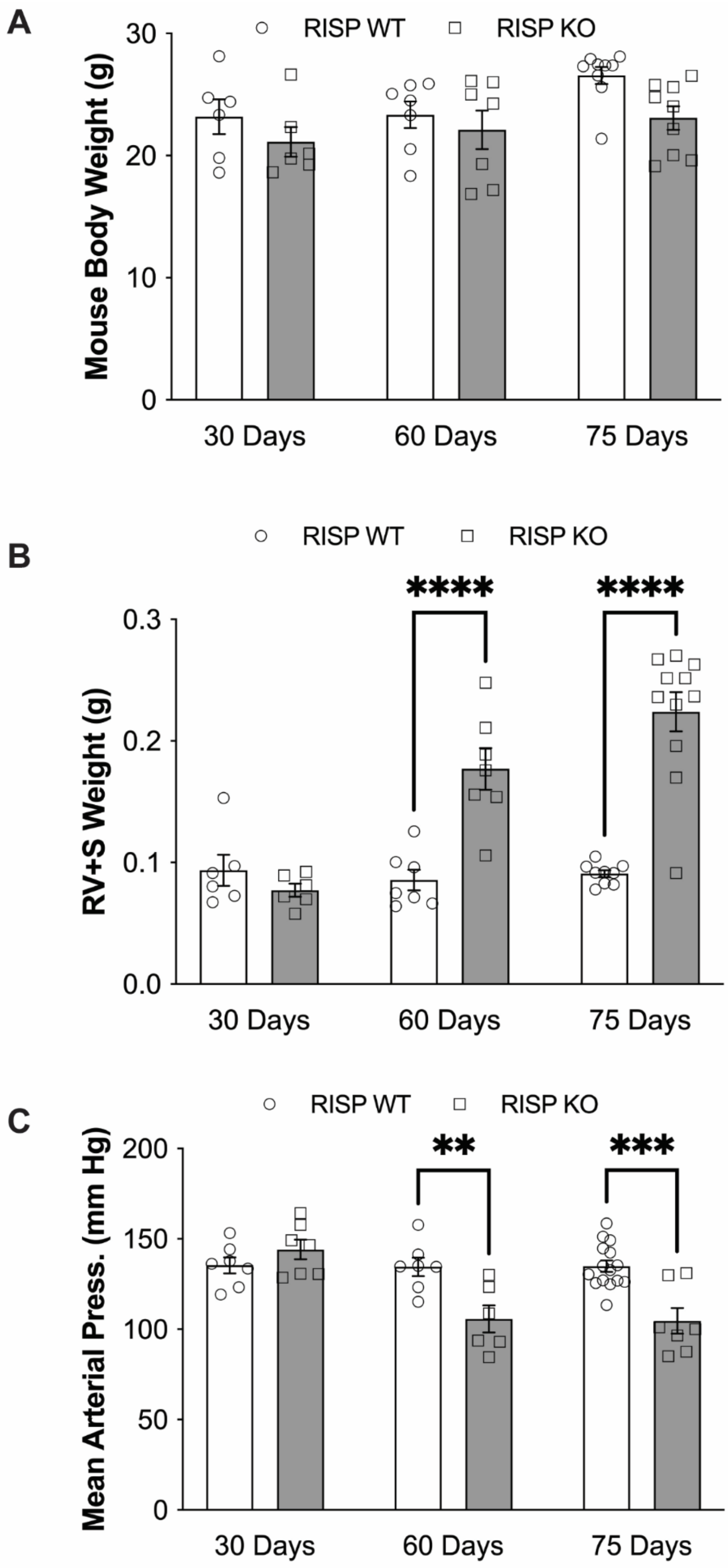

Supplemental Figure S2

Supplementary Figure S2. (**A**) There was no difference in the body weights of RISP WT versus RISP KO mice at 30, 60 and 75 days after tamoxifen administration, n=4-9 mice per condition, mean±SEM, 2W-ANOVA-Sidak's. (**B**) Right ventricle and septal weight of RISP WT and RISP KO hearts at 30, 60 and 75 days after tamoxifen administration, n=6-11 mice per condition, mean±SEM, 2W-ANOVA-Sidak's. Right ventricle and septal weights increased in the RISP KO hearts indicating total heart remodeling due to cardiac RISP depletion. (**C**) Mean systemic arterial pressure measured by tail cuff in unanesthetized RISP WT and RISP KO mice at 30, 60 and 75 days after tamoxifen administration, n=6-15 mice per condition, mean±SEM, 2W-ANOVA-Sidak's. For all graphs **p<0.01, ***p<0.001, ****p<0.0001.

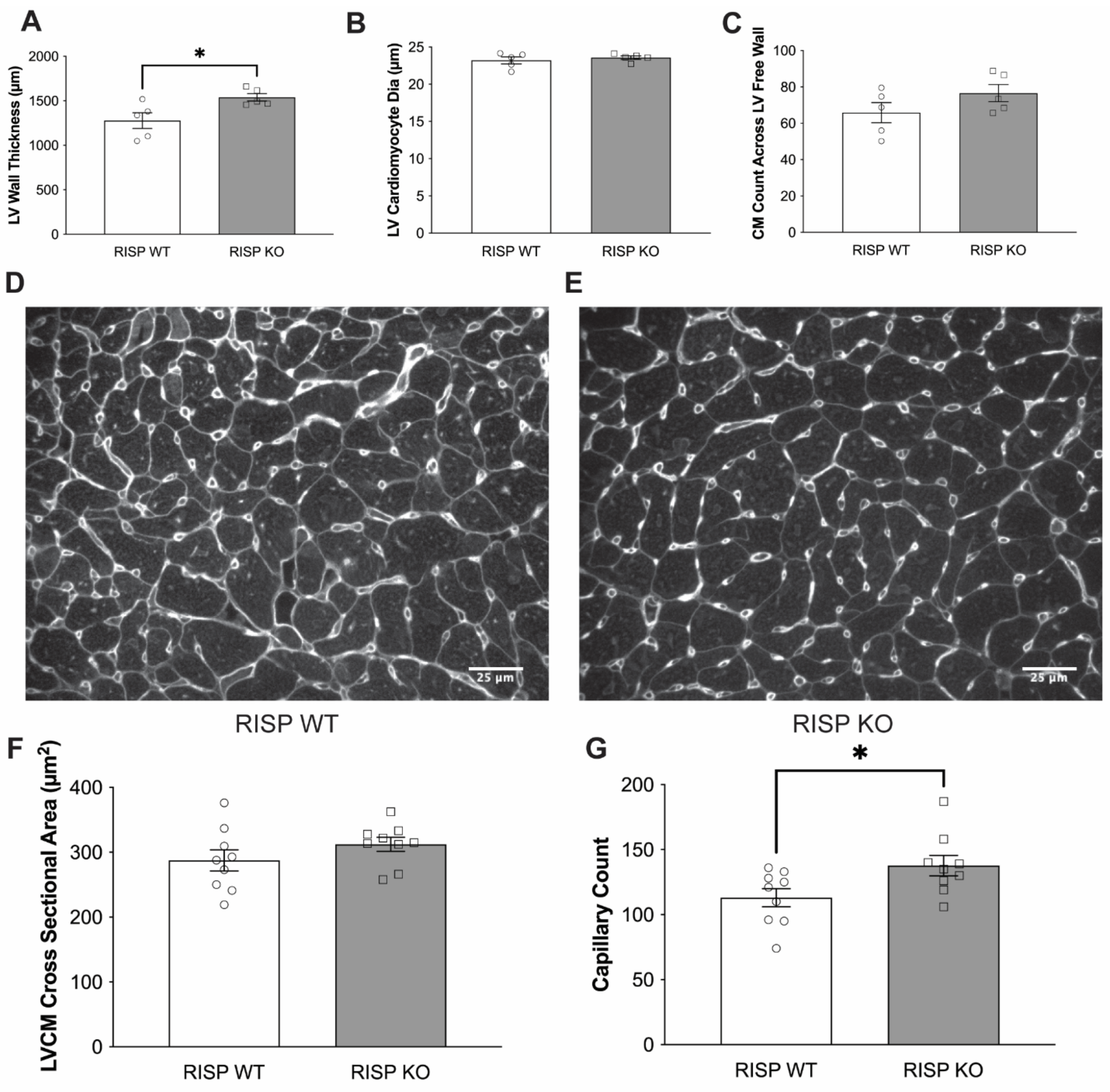

Supplemental Figure S3

Supplementary Figure S3. (**A**) LV wall thickness, assessed at random sites across the LV free wall, was increased in RISP KO hearts at 75 days post-tamoxifen, compared with WT controls, n=5 mice per condition, mean±SEM, U-2t-T-test. (**B**) Cardiomyocyte diameter at the nucleus, assessed in PAS-stained heart sections, was not different between RISP KO and WT hearts, n=5 mice per condition, mean±SEM, U-2t-T-test. (**C**) Cardiomyocyte counts across the left ventricle free wall was not different in RISP WT and RISP KO hearts, n=5 mice per condition, mean±SEM, U-2t-T-test. (**D, E**) WGA staining of LV sections from RISP WT and RISP KO hearts, respectively, at 60 days post-tamoxifen. Scale bars: 25 μm. (**F**) Left ventricle cardiomyocyte cross-sectional area assessed from WGA-stained samples was not different in RISP WT and RISP KO hearts, n=5 mice per condition, mean±SEM, U-2t-T-test. (**G**) Increased capillary density in remodeled RISP KO hearts compared with WT controls, n=9 mice, mean±SEM, U-2t-T-test. For all graphs $^*p<0.05$.

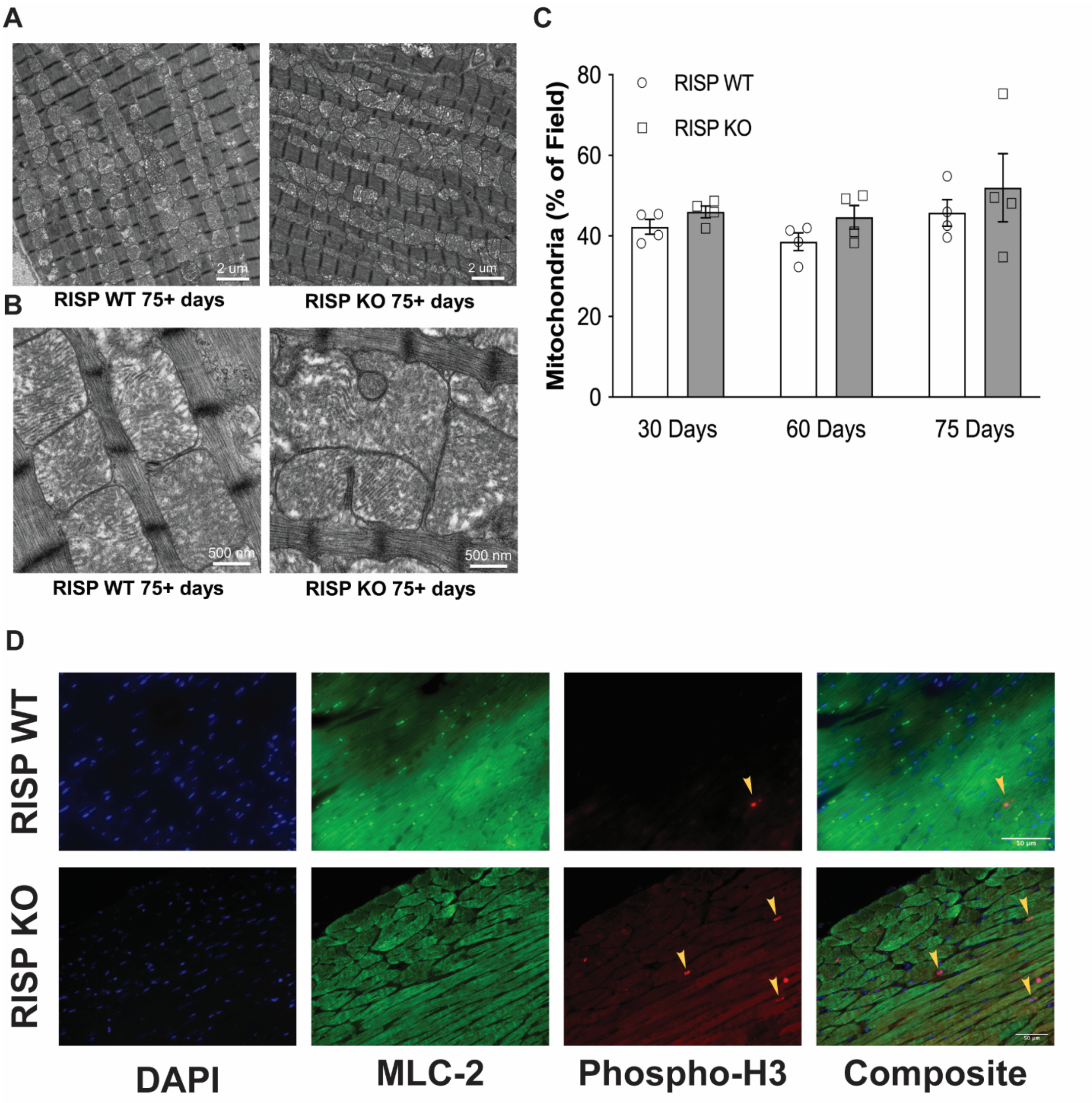

Supplemental Figure S4

Supplementary Figure S4. (**A**) Cardiac contractile fiber ultrastructure was normal and indistinguishable between RISP WT and RISP KO hearts. Scale bars: 2 μm. Magnified regions to illustrate mitochondrial ultrastructure (**B**) which was normal and indistinguishable between in RISP WT and RISP KO hearts. Scale bars: 500 nm. (**C**) Mitochondrial density assessed from TEM images of cardiac muscle from RISP WT and RISP KO mice at 30, 60 and 75 days after tamoxifen administration, n=4 mice per condition, mean±SEM, 2W-ANOVA-Sidak's. (**D**) Representative heart sections stained for DAPI, myosin light chain (MLC-2), and Phospho-H3 in RISP WT and KO hearts 60 days post tamoxifen. Yellow arrows denote Phospho-H3 positive nuclei that were counted as they co-localized with the nuclei of myosin light chain positive cardiomyocytes. Scale bars: 50 μm.

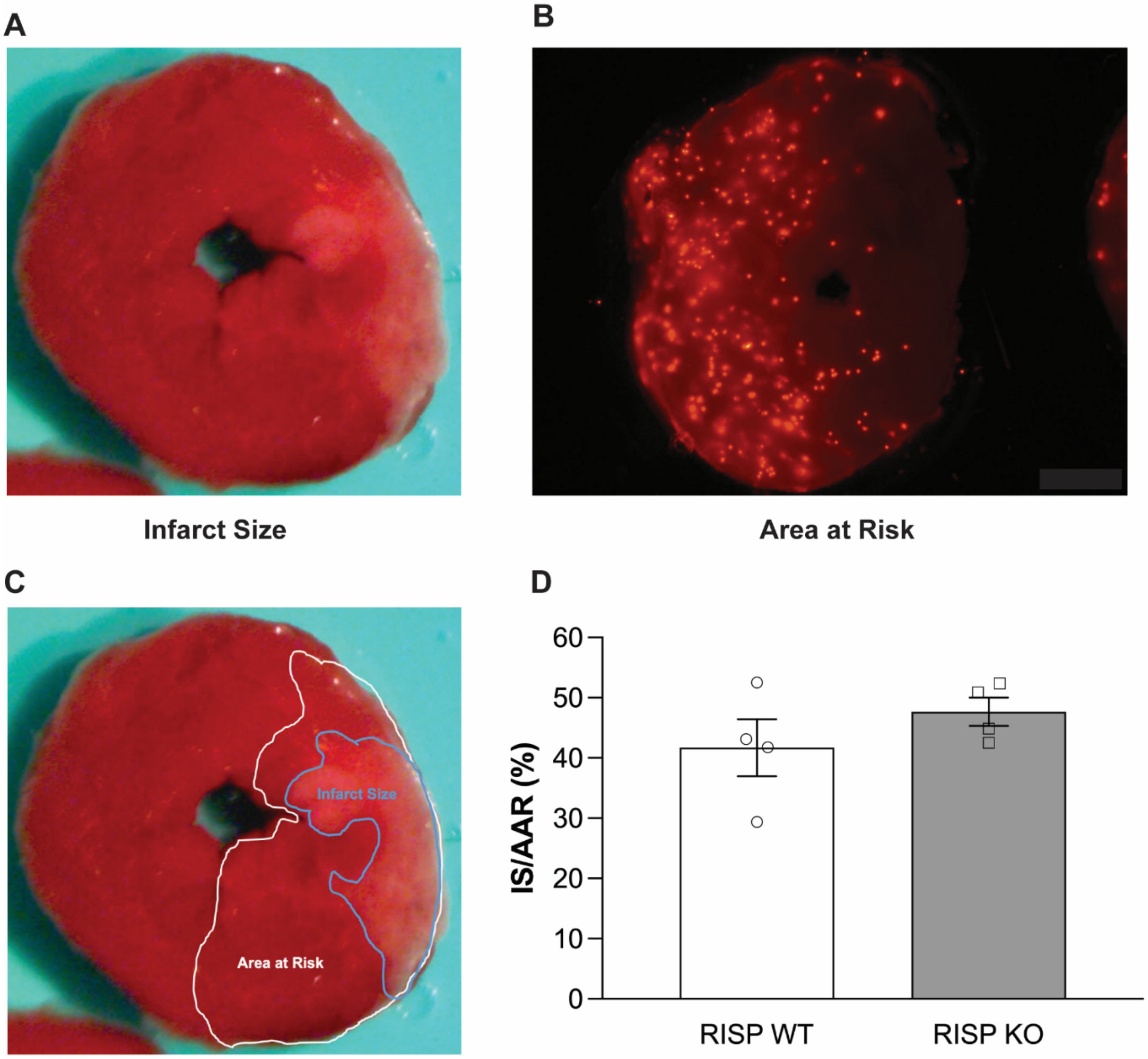

Supplemental Figure S5

Supplementary Figure S5. Fluorescent latex beads were perfused retrograde into the aorta and coronary arteries to determine the area at risk. Subsequently, hearts were removed and sliced into 1-mm transverse cross sections. The heart slices were incubated with 1% 2,3,5-triphenyl-tetrazolium chloride solution (TTC). Heart slices were then placed in 10% formalin for 30 mins for fixation. Viable area was stained red and infarcted area remained white. (**A**) Infarct size was determined by white area after TTC staining from a representative heart slice. (**B**) Area at risk (AAR) was determined by area with the fluorescently labeled microspheres from a representative heart slice. (**C**) Infarct size, AAR, and the total area of LV myocardium were traced manually in the digital images. (**D**) Infarct size, expressed as a percentage of the AAR, was calculated by dividing the sum of infarct areas from all sections by the sum of AAR from all sections, n=4 mice per condition, mean±SEM, U-2t-T-test.

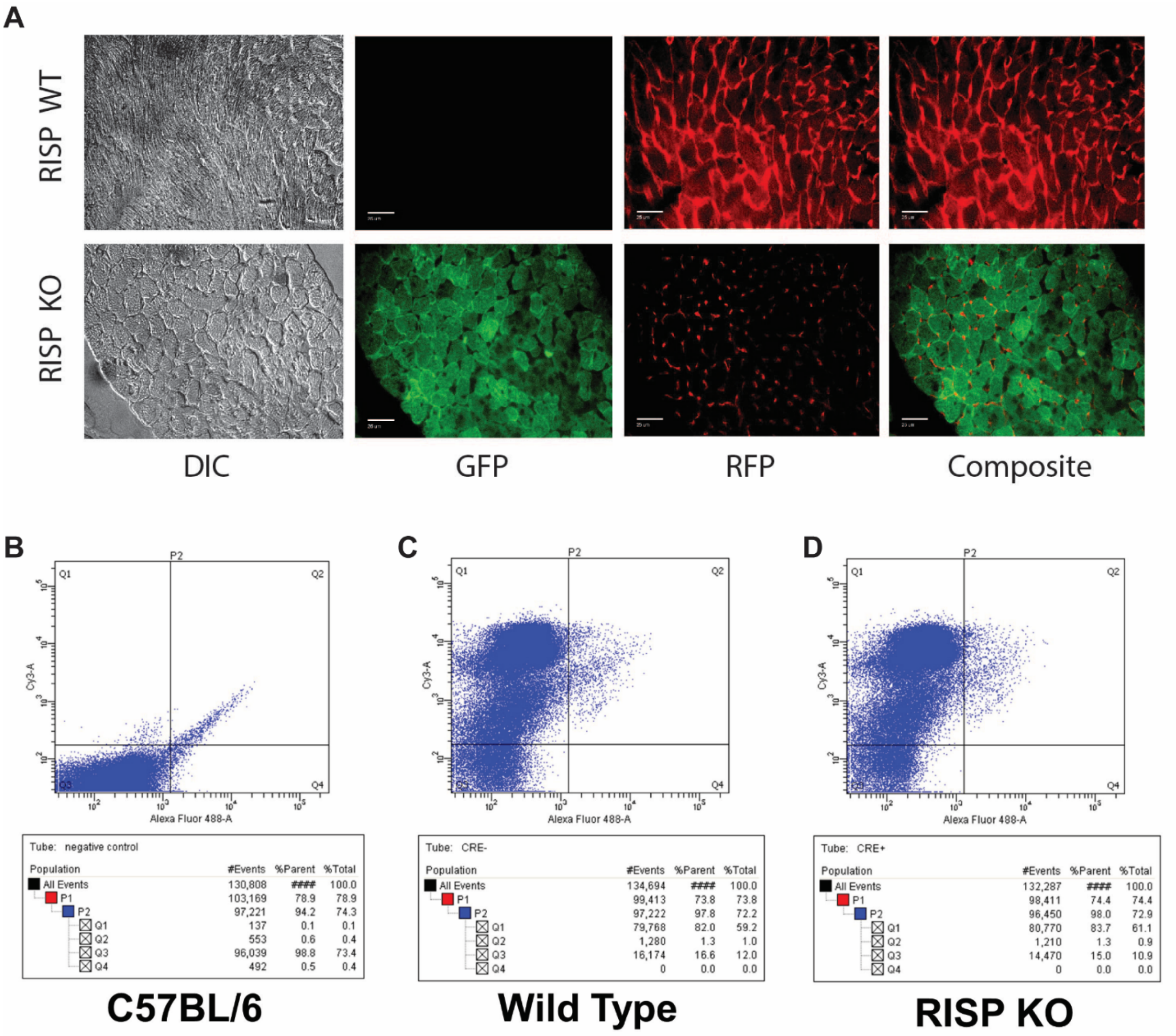

Supplemental Figure S6

Supplementary Figure S6. (**A**) Representative heart sections from RISP WT and RISP KO hearts carrying the mT/mG reporter transgene, at 60 days after tamoxifen, showing expected red fluorescence in WT and conversion to green fluorescence in KO hearts. Cardiomyocytes expressing red fluorescence were absent in hearts from RISP KO mice. Scale bars: 25 μm. Flow cytometry analysis of bone marrow cells from ordinary (**B**) C57BL/6 mice, as well as from (**C**) RISP WT and (**D**) KO mice carrying the mT/mG transgene, at 60 days after tamoxifen administration. Plots show red fluorescence (ordinate) against green fluorescence (abscissa).

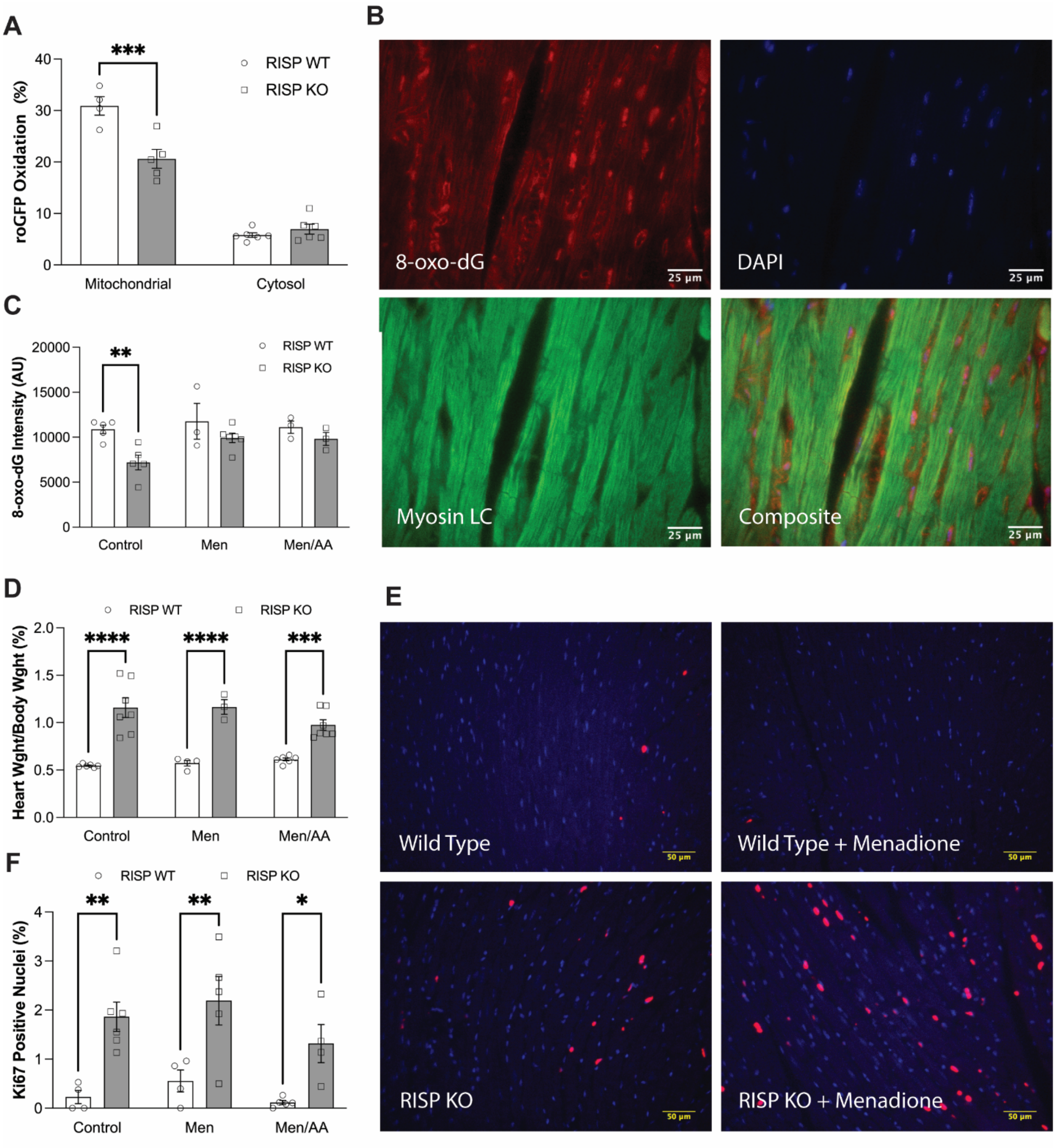

Supplement Figure S7

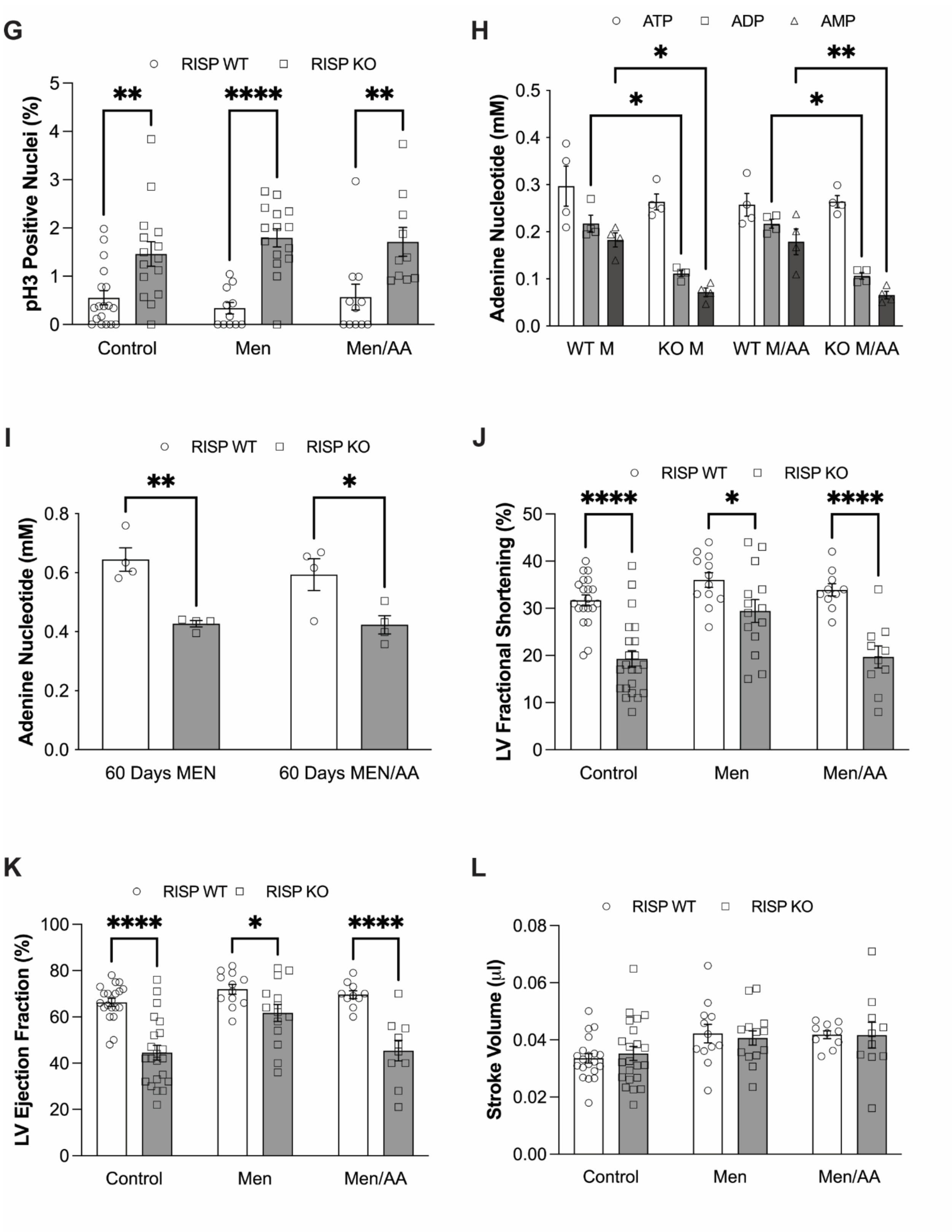

Supplemental Figure S7 Cont.

Supplementary Figure S7. (**A**) Mitochondrial matrix and cytosolic thiol oxidant stress in cultured cardiomyocytes from neonatal hearts of RISP$^{fl/fl}$ mice carrying creatine kinase promoter-driven Cre (RISP KO) or WT littermate controls assessed using the genetically encoded, ratiometric redox sensor, roGFP. Basal mitochondrial thiol redox status was less oxidized in RISP KO cells compared to WT, but cytosolic oxidant status was not different, n=4-6 hearts per condition, mean±SEM, ANOVA-Tukey's. (**B**) Representative fluorescence of 8-oxo-dG, myosin light chain and DAPI in heart sections, used to assess nuclear 8-oxo-dG fluorescence. Scale bars: 25 µm. (**C**) Nuclear fluorescence intensity in RISP WT and KO hearts from mice on control, menadione (120 mg/day) or menadione+ascorbate (120 mg/day each) soft diets, for days 30-60 post-tamoxifen, n=8-21 mice per condition, mean±SEM, ANOVA-Tukey's. (**D**) Heart weight/body weight ratio from RISP WT and KO hearts from mice on control, menadione (120 mg/day) or menadione+ascorbate (120 mg/day each) soft diets, for days 30-60 post-tamoxifen, n=4-7 mice per condition, mean±SEM, ANOVA-Tukey's. (**E**) Representative Ki-67 staining in RISP WT and KO hearts from mice on control and menadione (120 mg/day) soft diets. Scale bars: 50 µm. (**F**) Ki-67-positive nuclei in hearts from RISP WT and KO mice on control, menadione (120 mg/day) or menadione+ascorbate (120 mg/day each) soft diets, for days 30-60 post-tamoxifen, n=4-9 mice per condition, mean±SEM, ANOVA-Tukey's. (**G**) phospho-H3-positive nuclei in hearts from RISP WT and KO mice on control, menadione (120 mg/day) or menadione+ascorbate (120 mg/day each) soft diets, for days 30-60 post-tamoxifen, n=4-6 mice per condition, mean±SEM, ANOVA-Tukey's. (**H**) Adenine nucleotide levels in hearts from RISP WT and KO mice on control, menadione (120 mg/day) or menadione+ascorbate (120 mg/day each) soft diets, for days 30-60 post-tamoxifen, snap-frozen in situ, n=4 mice per condition, mean±SEM, ANOVA-Tukey's. (**I**) Total adenine nucleotide levels in RISP WT and KO hearts from mice on menadione (120 mg/day) or menadione+ascorbate (120 mg/day each) soft diets, for days 30-60 post-tamoxifen, n=4 mice per condition, mean±SEM, ANOVA-Tukey's. (**J**) LV fractional shortening, from echocardiography, of hearts from RISP WT and KO mice on control, menadione (120 mg/day) or menadione+ascorbate (120 mg/day each) soft diets, for days 30-60 post-tamoxifen, n=10-22 mice per condition, mean±SEM, ANOVA-Tukey's. (**K**) LV ejection fraction, from echocardiography, of hearts from RISP WT and KO mice on control, menadione (120 mg/day) or menadione+ascorbate (120 mg/day each) soft diets, for days 30-60 post-tamoxifen, n=10-22 mice per condition, mean±SEM, ANOVA-Tukey's. (**L**) Stroke volume from LV diameter assessed by echocardiography, of hearts from RISP WT and KO mice on control or menadione (120 mg/day) soft diets, for days 30-60 post-tamoxifen, n=10-22 mice per condition, mean±SEM, ANOVA-Tukey's. For all graphs *p<0.05, **p<0.01, ***p<0.001, ****p<0.0001.

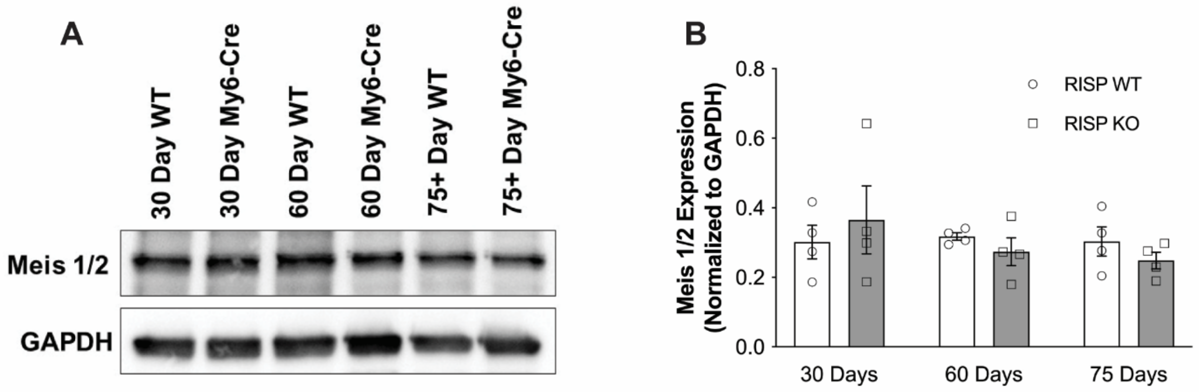

Supplemental Figure S8

Supplementary Figure S8. (**A**) Representative immunoblots of meis1/2 and GAPDH protein loading control in heart homogenates from RISP WT and RISP KO mice at 60 and 75 days after tamoxifen administration. Note: immunoblot is from the same membrane in Figure 1C, which was stripped and reblotted for meis1/2. (**B**) Band density analysis of immunoblots of meis1/2 and GAPDH loading control in heart homogenates from RISP WT and RISP KO mice at 60 and 75 days after tamoxifen administration, n=4 mice per condition, mean±SEM, 2W-ANOVA-Sidak's.

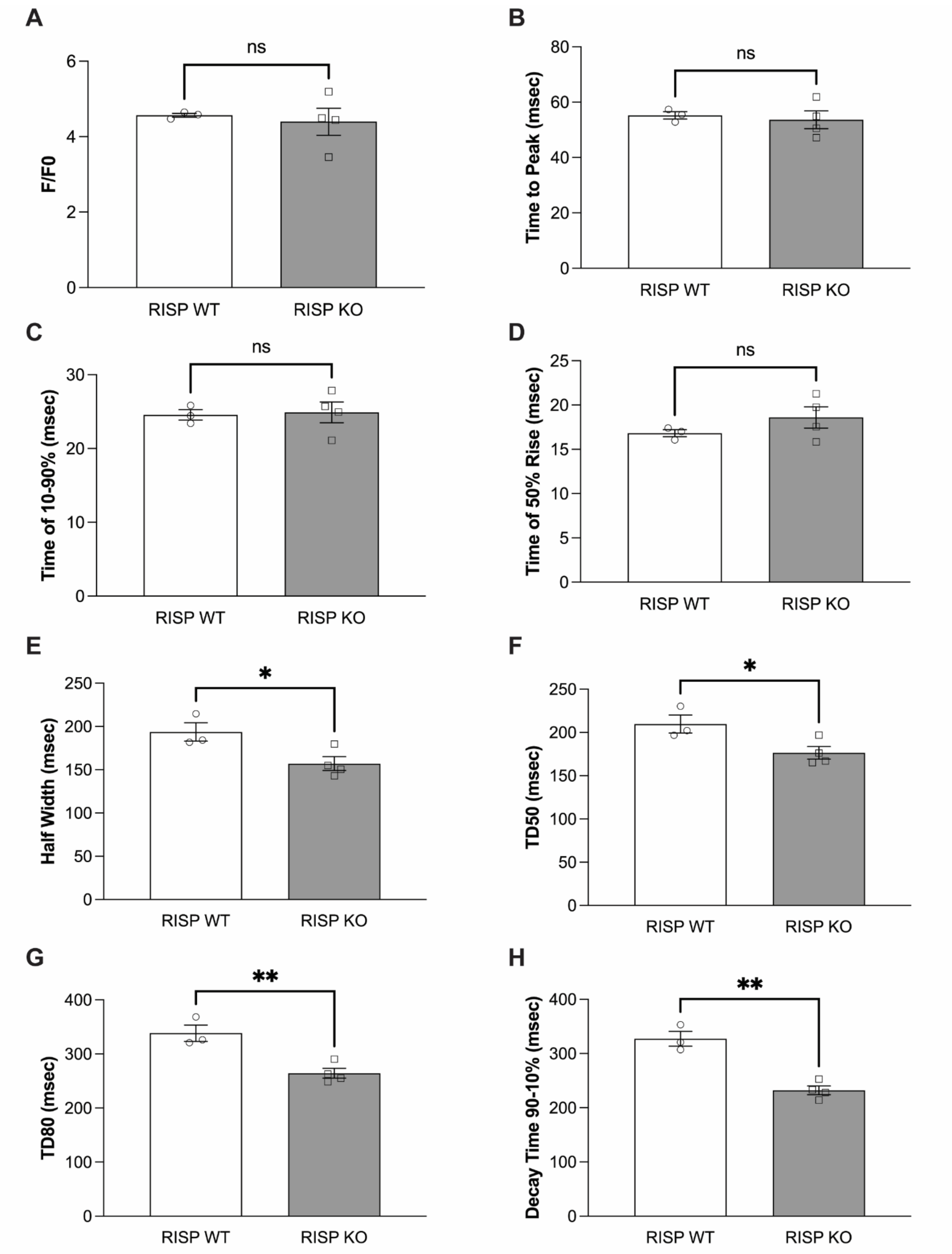

Supplemental Figure S9

Supplementary Figure S9. (**A-H**) Specific time analysis of the Ca^{2+} versus time curves assessed in individual electrically paced cardiomyocytes. Variable analysis of the increase in Ca^{2+} indicates no significant differences in the initial rise in Ca^{2+} (A-D) between cardiomyocytes isolated from RISP WT and RISP KO mice. However, variable analysis of decrease in Ca^{2+} indicated that the rate of decline in Ca^{2+} (E-H) was faster in the cardiomyocytes from the RISP KO mice compared to the RISP WT mice, n=3-4 mice per condition with 10-20 individual cardiomyocytes per mouse tested, mean±SEM, U-2t-T-test. For all graphs *$p<0.05$, **$p<0.01$.

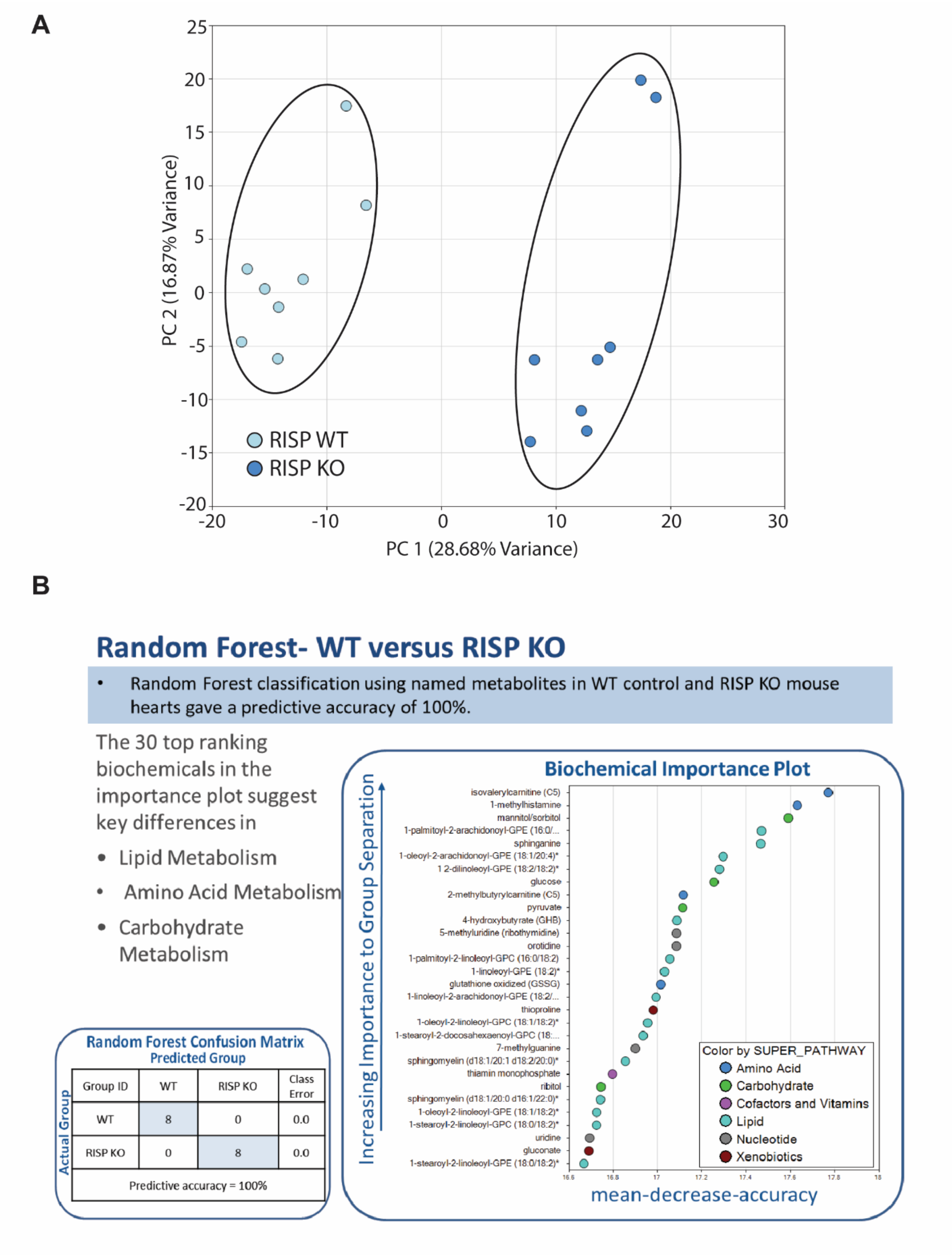

Supplemental Figure S10

Supplementary Figure S10. (**A**) Principal component analysis of metabolomic data in RISP WT (n=8) and RISP KO (n=8) hearts, snap-frozen in situ. (**B**) Random Forest analysis/biochemical importance plot of metabolite differences between RISP WT and RISP KO mice at 60 days after tamoxifen administration identifies key differences in lipid, amino acid, and carbohydrate metabolism.

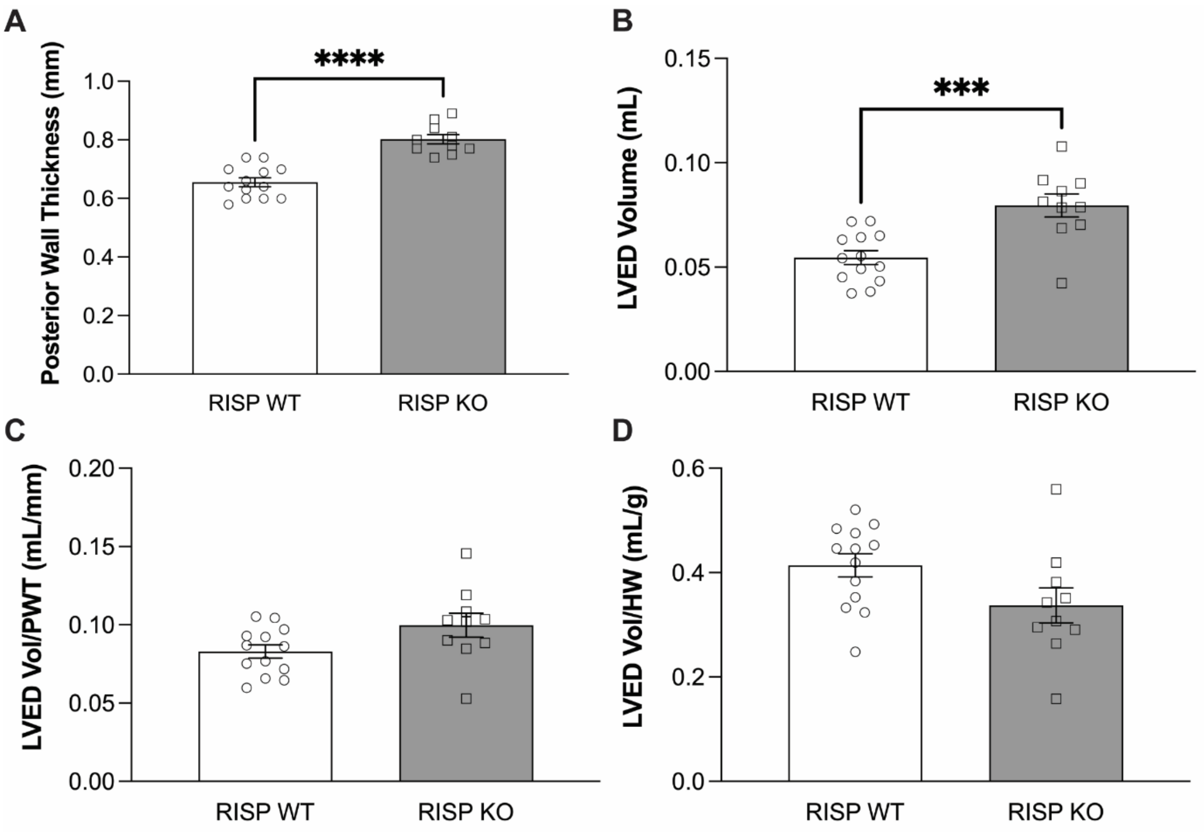

Supplemental Figure S11

Supplementary Figure S11. (**A**) Posterior Wall Thickness (PWT), assessed by echocardiography, depicted a small by significant increase at 60 days post-tamoxifen in RISP KO hearts, compared with WT, n=10-13 mice per condition, mean±SEM, U-2t-T-test. (**B**) LV end-diastolic volume (LVED Vol), assessed by echocardiography, increased at 60 days post-tamoxifen in RISP KO hearts, compared with WT, n=10-13 mice per condition, mean±SEM, U-2t-T-test. (**C**) The LVED Vol divided by the PWT was calculated for each mouse and showed no difference between RISP WT and RISP KO mice at 60 days post-tamoxifen, n=10-13 mice per condition, mean±SEM, U-2t-T-test. (**D**) The LVED Vol divided by the heart weight (HW) was calculated for each mouse and showed no difference between RISP WT and RISP KO mice at 60 days post-tamoxifen, n=10-13 mice per condition, mean±SEM, U-2t-T-test. For all graphs ***p<0.001, ****p<0.0001.

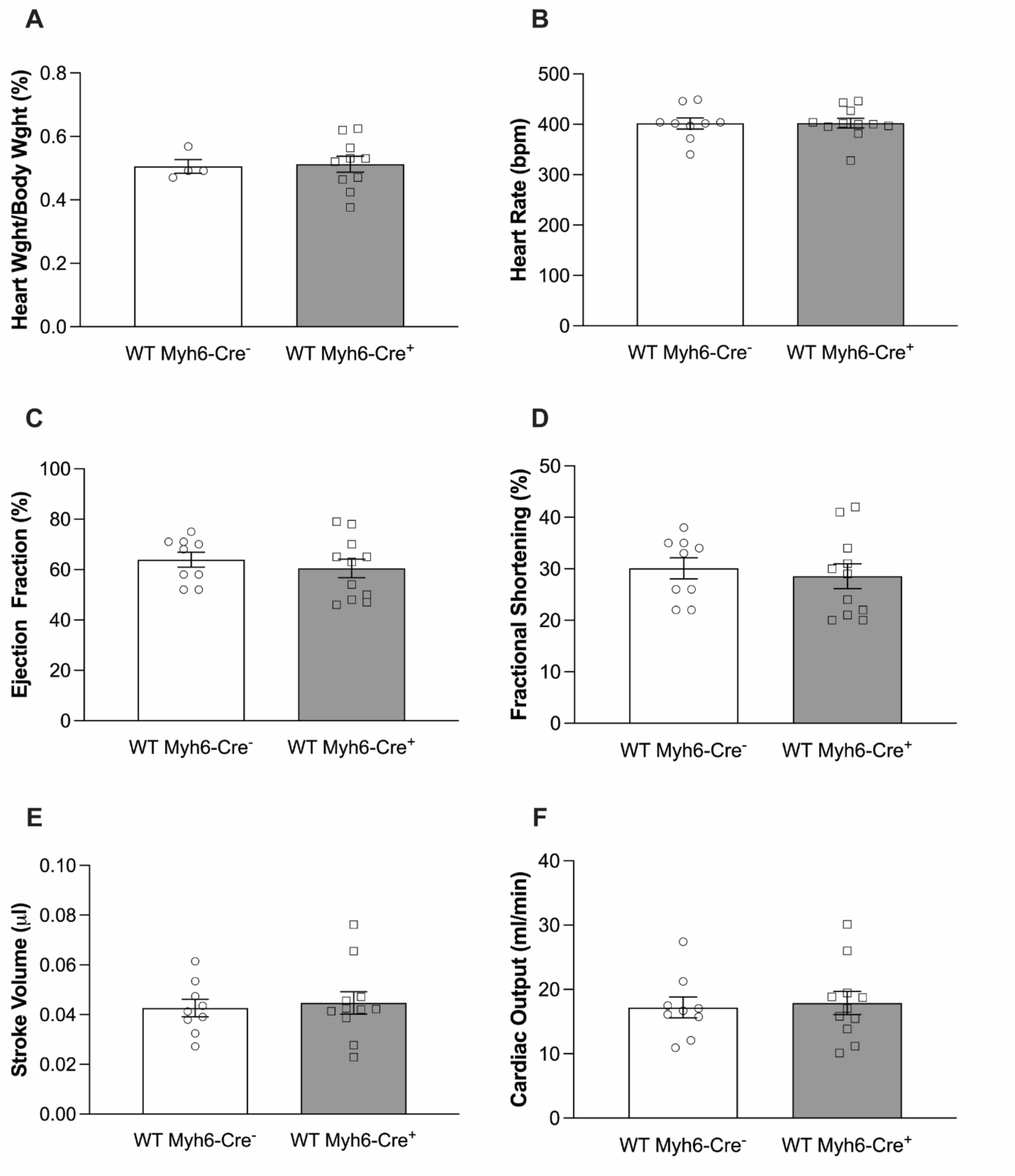

Supplemental Figure S12

Supplementary Figure S12. Comparisons of Charles River C57BL/6 mice carrying the Myh6-Cre gene (WT Mhy6-Cre+) compared to littermate control mice lacking the gene (WT Myh6-Cre-) demonstrated no phenotypical differences at 60 days post tamoxifen. (**A**) Heart weight/body weight ratios, n=4-10 mice per condition, mean±SEM, U-2t-T-test. (**B**) Heart rate in anesthetized RISP WT and RISP KO mice while undergoing echocardiography measurements, n=9-11 mice per condition, mean±SEM, U-2t-T-test. (**C**) LV ejection fraction, assessed by echocardiography, n=9-11 mice per condition, mean±SEM, U-2t-T-test. (**D**) LV fractional shortening, assessed by echocardiography, n=9-11 mice per condition, mean±SEM, U-2t-T-test. (**E**) Stroke volume in anesthetized RISP WT and RISP KO mice while undergoing echocardiography measurements, n=9-11 mice per condition, mean±SEM, U-2t-T-test. (**F**) Cardiac output, calculated as the product of heart rate and end-diastolic minus end-systolic diameter, n=9-11 mice per condition, mean±SEM, U-2t-T-test.

Detailed Methods

Mice: Conditional deletion of RISP (*Uqcrfs1*, NM_025710) was engineered by flanking exon 2 of the RISP gene with loxP sites (RISP$^{fl/fl}$) as previously described (1). To delete RISP in cardiomyocytes of adult mice, RISP$^{fl/fl}$ mice were bred with a transgenic line expressing tamoxifen-inducible Cre recombinase under the control of a cardiac-specific α-myosin heavy chain promoter (Myh6-Cre) (2). At 5-6 weeks of age, tamoxifen (0.5 mg, i.p.) dissolved in corn oil was administered to the resulting mice (Myh6-Cre/RISP$^{fl/fl}$) for five consecutive days to activate Cre-mediated excision of the RISP exon 2 in cardiomyocytes. Littermate Cre-negative control mice received the same tamoxifen dosing. Studies began a minimum of 30 days after the last dose of tamoxifen. All mice were in the C57BL/6 genetic background.

mT/mG fluorescent reporter mice: (*Gt(ROSA)26Sor*$^{tm4(ACTB\text{-}tdTomato,\text{-}EGFP)Luo}$, stock #007576, The Jackson Laboratory, Bar Harbor, ME, USA) (3) were crossed with Myh6-Cre mice. The reporter mice carry *loxP* sites on either side of an mT cassette and basally express red fluorescent protein in all cell types. Cre expression induces deletion of the mT cassette, activating expression of a membrane-targeted EGFP (mG) cassette located 3' to the deletion site. Tamoxifen (0.5 mg, i.p.) dissolved in corn oil was administered to the resulting mice at 5-6 weeks of age, for five consecutive days, to activate Cre-mediated excision of the mT cassette in the cardiomyocytes, causing the expressed fluorescent protein to shift from red to green. After allowing 2 weeks to complete this process, the hearts and livers were removed and dehydrated in 15% sucrose, followed by 30% sucrose. Hearts and livers were then embedded in OCT (Tissue Tek, Sakura Finetek, Torrance, CA, USA) and frozen in a dry ice-ethanol bath. Frozen blocks were stored at -80°C prior to cryotome sectioning (Leica CM-1850) to a thickness of 10 µm. Tissue slices were then stained with DAPI. Cells were observed under epifluorescence microscopy using a 40x, 1.3 NA Plan Fluor objective in an inverted microscope (Nikon, Melville, NY, USA), and imaged with a cooled CCD camera (Photometrics Cascade, Tucson, AZ, USA) controlled by a computer workstation running Metamorph imaging software (Universal Imaging, Bedford Hills, NY, USA).

Neonatal mouse cardiomyocytes: RISP$^{fl/fl}$ mice were bred with transgenic mice expressing constitutively active Cre recombinase driven by the muscle creatine kinase promoter (B6.FVB(129S4)-Tg(Ckmm-cre)5Khn/J, MCK-Cre, The Jackson Laboratory, stock #006475) to generate RISP$^{fl/+}$±MCK-Cre mice. Those mice were bred with RISP$^{fl/fl}$; the offspring were genotyped within 24 hr after birth to identify MCK-Cre-RISP$^{fl/fl}$ and Cre-negative RISP$^{fl/fl}$ controls. Cardiac myocytes were then isolated from individual mice according to their genotype, as previously described (4).

Cardiomyocyte isolation from mouse hearts: Cardiomyocytes (CMs) were isolated from mouse hearts as previously described (5). Briefly, mice were anesthetized and cut open below the diaphragm to expose the heart. After injection of 7 mL EDTA buffer to the right ventricle, the aorta was clamped by forceps and the heart was removed. Multiple rounds of injections to the apex of the heart were performed with the following: 10 mL EDTA buffer, 3 mL of perfusion buffer, and 100 mL of collagenase buffer. The hearts were then pulled apart using forceps and filtered through 100 µm strainers to remove large tissue debris. After 3 rounds of gravity settling, the adult CMs were resuspended in culture media and utilized immediately for experiments.

Ca^{2+} measurements: Isolated ventricular myocytes (see above) were loaded with Rhod-4AM (14mM for 30 minutes) then washed for at least 10 minutes. An aliquot of cells was then placed in an experimental chamber (Cell MicroControls, Norfolk, VA, USA) on the stage of a laser scanning confocal microscope (Zeiss LSM510 Meta, Zeiss, Silicon Valley, CA, USA)). Perfusion with modified Tyrodes solution and pacing at a basic cycle length of 1000msec were initiated. Cells were illuminated with a HeNe laser at 533 nM and light was collected at 543 nm and above. Line scan rate was 1.92 msec per line at a resolution of 512 pixels per line. Data analysis on individual Ca2+ transients was performed using Zeiss LSM510 software (Zeiss, Silicon Valley, CA, USA) and pClamp 10.4 (Molecular Devices, San Jose, CA, USA). Statistical analysis was performed using SigmaPlot 11 (Systat, Palo Alto, CA, USA).

Genotyping: All mouse genotyping was carried out by Transnetyx, Inc. (Cordova, TN, USA) using the following primer sequences: Myh6-Cre forward TTAATCCATATTGGCAGAACGAAAACG, Myh6-Cre reverse CAGGCTAAGTGCCTTCTCTACA, Myh6-Cre reporter CCTGCGGTGCTAACC; RISP^{FL} forward CCAAACTGGCCTCAAACTTGTAATC, RISP^{FL} reverse AGAGAATAGGAACTTCGGAATAGGAACT, RISP^{FL} reporter CTGTGCTTGCTGGTTAAT; RISP^{WT} forward CTGGCCTCAAACTTGTAATCTTCCT, RISP^{WT} reverse, TGATGGCACATGCCTCTCAT, RISP^{WT} reporter CCGAGACCCAGCAAGC; mT/mG forward CGTCGTCCTTGAAGAAGATGGT, mT/mG reverse CACATGAAGCAGCACGACTT, mT/mG reporter CATGCCCGAAGGCTAC; MCK-Cre forward GCTCACAGGGTCCCAAAGG, MCK-Cre reverse ATTGGACACCTTCCTCTTCTTCTTG, MCK-Cre reporter ACGCCCTCGACCATGC.

Menadione/Ascorbic acid diet: Beginning at 30 days post-tamoxifen, RISP WT and RISP KO mice were placed on a soft food diet (DietGel 76A soft diet packs, ClearH_2O,

Portland, ME, USA) into which was mixed menadione sodium bisulfite (120 mg/kg b.w./day; Sigma Life Sciences) or menadione sodium bisulfite (120 mg/kg b.w./day; Sigma Life Sciences) and ascorbic acid 120 mg/kg b.w./day; Sigma Life Sciences), until 60 days post-tamoxifen. Food packs were replaced as needed to provide *ad libitum* nutrition.

Micro osmotic pump insertion: At 30 days post-tamoxifen, mice were anesthetized with isoflurane and a small incision was made at the nape of the neck. A sterile micro-osmotic pump (Model 1004, Alzet, Cupertino, CA, USA) loaded with EdU (0.3 mg/kg b.w./day) dissolved in DMSO (50% by vol; Sigma Life Sciences) and Polyethylene Glycol 400 (50% by vol; EMD Millipore Corporation, Billerica, MA, USA) was implanted into a deep subcutaneous pocket along the back of the neck. The skin layer was closed using wound clips and the mouse was allowed to recover.

Cardiomyocyte Isolation: Cardiomyocyte dimensions and numbers were obtained using methodology like that previously reported (6). The mT/mG fluorescent reporter mice were crossed with Myh6-Cre mice or Myh6-Cre/RISP$^{fl/fl}$ mice to generate mT/mG/Myh6-Cre mice or mT/mG/Myh6-Cre/RISP$^{fl/fl}$ mice. Tamoxifen (0.5 mg, i.p.) dissolved in corn oil was administered to the resulting mice at 5-6 weeks of age, for five consecutive days to activate the Cre. Sixty days post tamoxifen, the hearts were harvested, washed in PBS, and weighed. The hearts were then stored at -80°C prior to cardiomyocyte isolation. To isolate cardiomyocytes, the hearts were quartered and fixed in 4% PFA for 2 hours at room temperature with gentle rocking. The heart samples were subsequently incubated in collagenase D (2.4 mg/ml, Roche, Basel, Switzerland) and B (1.8 mg/ml, Roche, Basel, Switzerland) for 5 days at 37°C with gentle rocking. The supernatant was collected and spun down at 1100 RPM for 10 min. The supernatant was decanted, and the cell pellet was resuspended in 1 ml of PBS and transferred to a tared 1.5 ml tube. The resuspension was spun down at 10,000 RPM for 15 min, the supernatant was decanted, and the resulting cell pellet weight was measured. The cell pellet was resuspended in 1 ml PBS. Cardiomyocyte count was determined by hemocytometer and the weight of the pellet was used to extrapolate the total number of cardiomyocytes in the original whole heart. For isolated cardiomyocyte measurements, the cardiomyocytes were stained with DAPI, and an aliquot was imaged under epifluorescence microscopy using a 40x, 1.3 NA Plan Fluor objective in an inverted microscope (Nikon, Melville, NY, USA), and imaged with a cooled CCD camera (Photometrics Cascade, Tucson, AZ, USA) controlled by a computer workstation running Metamorph imaging software (Universal Imaging, Bedford Hills, NY, USA) and analyzed using ImageJ (NIH). Only mG fluorescent cardiomyocytes were analyzed as those were the ones confirmed as positive for activated Cre. Individual isolated cardiomyocyte length and width was measured using ImageJ (NIH). The DAPI-stained nuclei count per cardiomyocyte was determined and the cardiomyocytes were designated as mononucleated, binucleated, or polynucleated (3 or more nuclei). Nuclei count was reported as a percentage of the assessed cardiomyocytes. Approximately 40-45 cardiomyocytes per mouse were measured/assessed to determine an average for that mouse, with 6-10 mice per experimental group.

Bone marrow progenitor cells: To determine if Myh6-Cre-positive bone marrow progenitor cells could contribute to the increase in RISP KO heart sizes, bone marrow was isolated from C57BL/6, RISP WT and RISP KO mice carrying the mT/mG transgene at 60 days post-tamoxifen. The mice were anesthetized with isoflurane and then subjected to cervical dislocation. The femurs were resected, and the bone marrow was flushed with 500 µL of ice-cold PBS and then analyzed by flow cytometry using a BD FacsAria SORP 5-Laser cell sorter.

LAD Occlusion: The Left Anterior Descending coronary artery (LAD) ligation was performed in RISP WT and RISP KO mice at 45d post tamoxifen as previously described (7). A 7-0 silk suture tie-off of the LAD was performed 3 mm below the left atria. Once the mice had recovered from the surgery, the experiment was continued for the next 15 days until 60d post tamoxifen during which time hyperplastic remodeling of the RISP KO hearts occurred.

Determining Infarct Size (IS) versus Area At Risk (AAR): The infarct size was measured as previously described (8). To be brief, the mice were anesthetized and intubated. The lower rib cage was removed to expose the heart. Fluorescent latex beads were perfused retrograde into the aorta and coronary arteries to determine the area at risk. Subsequently, hearts were removed and sliced into 1-mm transverse cross sections. The heart slices were incubated with 1% 2,3,5-triphenyl-tetrazolium chloride solution (Sigma T8877) for 15 min at 37°C. Heart slices were then placed in 10% formalin for 30 mins for fixation. Viable area was stained red and infarcted area remained white. Heart sections were weighed before digital photomicrographs were taken. Images of infarcts were blinded for analysis. Area at risk (AAR) was determined by area with the fluorescently labeled microspheres. Infarct area (white area after TTC staining), AAR, and the total area of LV myocardium were traced manually in the digital images. Infarct size, expressed as a percentage of the AAR, was calculated by dividing the sum of infarct areas from all sections by the sum of AAR from all sections.

Determining Margin Smoothness: Quantification of permeation of the MI region in the mouse hearts by proliferating cardiomyocytes was performed using ImageJ (NIH). First the margin between necrotic tissue and non-necrotic tissue was traced and the parameter of the resulting tracing was measured. Then a best fit ellipse with a cross-sectional area equal to the traced region was generated by ImageJ and the parameter of the resulting ellipse was measured. Dividing the parameter of the best fit ellipse by the parameter of the tracing was done to calculate the Margin Smoothness ($Dia_{ellipse}/Dia_{tracing}$). The Margin Smoothness is defined as a number that approaches one (1) if there is little difference between $Dia_{ellipse}$ and $Dia_{tracing}$. This correlates with the concept that there is no invasion of non-necrotic cardiomyocytes into the MI region. Conversely the Margin Smoothness approaches zero (0) as the MI region is increasingly permeated with dividing, non-necrotic cardiomyocytes. 6-8 Serial transverse heart slices were measured and averaged per mouse.

Heart harvest procedures:

- *Isoflurane*: RISP WT and RISP KO mice 30-, 60-, and 75-days post-tamoxifen were anesthetized with isoflurane and then subjected to cervical dislocation. The mice were then quickly weighed, the thoracic cavity was opened, and the heart was resected and dropped into ice cold PBS. Hearts were blotted semi dry on gauze and weighed. The heart was then transversely bisected after which the base was placed in 4% buffered formalin to fix for histology and the apex was frozen in liquid nitrogen and stored at -80ºC. The frozen heart tissue was used for protein analysis, RNAseq, or epigenetics.
- *Snap frozen*: Mice were anesthetized with avertin (240 mg/kg, i.p.) and the trachea was cannulated with a 20 ga blunt needle. The mouse was then mechanically ventilated using a small animal respirator (Harvard) at 150 breaths/min and a tidal volume 200 µl. The thoracic cavity was opened by median sternotomy and the still-beating heart was snap-frozen using a large pair of surgical forceps that had been submerged in liquid nitrogen. The snap-frozen heart was then quickly resected and submerged in liquid nitrogen to sustain the freezing process; the heart was subsequently placed in a cryotube and returned to the liquid nitrogen. The frozen hearts were later stored at -80ºC. These hearts were used for protein analysis, adenine nucleotide measurements, and metabolomics.

FDG-PET and microCT: Mice were anesthetized in an induction chamber with 3% isoflurane in oxygen, weighed, and then transferred to a dedicated imaging bed with isoflurane delivered via nosecone at 1-2%. Mice were placed in the prone position on a plastic bed and immobilized to minimize changes in position between scans. Respiratory signals were monitored using a digital monitoring system developed by Mediso (Mediso-USA, Boston, MA). Mice were imaged with a preclinical microPET/CT imaging system (nanoScan PET/CT, Mediso-USA, Boston, MA). CT data was acquired with a 2.2x magnification, <60 µm focal spot, 2 × 2 binning, with 480 projection views over a full circle, using 50 kVp/520 µA, with a 300 ms exposure time. The projection data was reconstructed with a voxel size of 250 µm and using filtered (Butterworth filter) back projection software from Mediso. For PET imaging, a target of 7-8 MBq of ^{18}F-fluordeoxyglucose (FDG) was injected intravenously. Mice are typically fasted for four hours with FDG-PET imaging. However, these mice would likely not survive fasting. PET acquisition parameters were as follows: 1:1 coincidence detection and 30-minute acquisition time. MLEM reconstruction was used with CT for attenuation correction and scattering. Pixel size was set to 0.3 × 0.3 mm. Analysis: The reconstructed data was visualized and analyzed in Amira 202.2 (FEI, Houston, TX). A region of interest (ROI) was drawn using the x-ray microCT data, with FDG-PET overlay. Lungs and skeleton ROI were generated for each mouse. The % injected dose (%ID) of FDG in the heart tissue was calculated by dividing the total PET signal found in the ROI with the injected dose for each mouse.

Isolated mitochondria studies: Hearts from RISP WT and KO 60 day mice were harvested and immediately chilled in ice-cold buffer, Mitochondrial studies were performed using a modification of previously described methods (9). Mitochondrial function was assessed in a respirometer at 34° C (Hansatech Oxygraph) in the

presence of ADP (2 mM), glutamate (10 mM), malate (5 mM) and rotenone (0.5 μM). Respiration at complex II was then stimulated with succinate (10 mM) followed by antimycin A (2 μM) plus myxothiazol (2 μM) to block electron transport out of and into complex III, respectively. Finally, respiration at complex IV was assessed by delivering electrons directly to cytochrome c (downstream from the complex III site of inhibtion) using N,N,N',N'-Tetramethyl-p-phenylenediamine dihydrochloride, (TMPD, 0.5 mM) and ascorbate (2 mM). Absolute rates of respiration were reported.

In other studies, hearts from RISP WT and RISP KO mice at 75 days post-tamoxifen were anesthetized with avertin (240 mg/kg, i.p.) and the thoracic cavity was opened by median sternotomy. The heart was resected and immediately plunged into ice-cold PBS. Hearts were then minced under ice-cold conditions; tissue fragments were washed with mitochondrial isolation buffer (Mannitol (225 mM), sucrose (75 mM), HEPES potassium salt (10 mM), bovine serum albumin (fatty acid free, 0.1%), EDTA (1 mM), pH 7.4) and subsequently disrupted using a Dounce homogenizer, while on ice, using the same mitochondrial buffer solution to suspend the organelles. Mitochondria were then isolated by differential centrifugation. Mitochondrial respiration was assessed in aliquots transferred to a respirometer (Hansatech) containing isolation buffer at 37° C, under baseline conditions, after addition of ADP (15 mM), pyruvate (1 mM), succinate (10 mM), then after addition of antimycin A (2.5 μm), and then after addition of TMPD, (0.5 mM) and ascorbic acid (5 mM). The ratio of mitochondrial respiration on succinate plus pyruvate to the rate on TMPD plus ascorbate was then calculated. Replicate assays (n=3-4) were carried out for each heart, and replicate studies were carried out using different mice (n=3-6).

Redox measurements of oxidant stress: Cardiomyocytes isolated from 1-3 day old $RISP^{fl/fl}$±MCK-Cre mice were maintained in culture (DMEM plus10% FBS plus penicillin) on laminin-coated 25mm #2 glass cover slips for 14 days. Cells were then transduced with an adenovirus to express the ratiometric thiol redox sensor, roGFP, targeted to the cytosol or to the mitochondrial matrix, as previously described(1, 10, 11). After allowing a minimum of 36 hours for expression, cover slips carrying the cells were transferred to a flow-through chamber (Penn-Century, Philadelphia, PA) mounted on a heated (37° C) stage insert on an inverted fluorescence microscope (Nikon). The chamber was perfused (1 ml/min) with buffered salt solution (BSS, NaCl (117 mM), KCl (4 mM), $NaHCO_3$ (18 mM, $MgSO_4$ (0.76 mM) NaH_2PO_4 (1 mM), $CaCl_2$ (1.2. mM), and glucose (5.6 mM)) equilibrated with 21% O_2/5% CO_2, balance N_2). Paired images were acquired at an emission wavelength of 535 nm, during excitation at 402 and 484 nm, using a Nikon Plan Fluor 40X 1.4 NA oil immersion objective. Individual cells were identified as regions of interest, and images were collected every 60 sec for 5-10 min, to obtain a stable baseline. The perfusate was then switched to BSS containing dithiothreitol (DTT, 2 mM) to fully reduce the roGFP sensor, while image acquisition continued. The DTT solution was then washed out, and the perfusate was switched to BSS containing t-butyl hydroperoxide (tBH, 2 mM) to fully oxidize the sensor, while data acquisition continued. Percent oxidation of the sensor during baseline was then calculated using the Rmax and Rmin values obtained with DTT and tBH, as previously described (1). Individual

cell values were averaged for the experiment, and replicate experiments were carried out for each genotype (n=6-8).

Echocardiography: Cardiac systolic and diastolic function, along with wall thicknesses, were assessed in isoflurane-anesthetized mice by echocardiography using a GE Vivid 7 System (GE Healthcare, Milwaukee, WI) with an i13L transducer (equipped with gray scale, color, and pulsed wave Doppler capabilities). The left ventricle (LV) ejection fraction (EF), fractional shortening (FS), LV dimensions (LV end-diastolic and end-systolic diameter), posterior wall thickness (PWT), LV end-diastolic volume (LVED Vol), heart rate (HR) and cardiac output (CO) were measured to assess cardiac function.

Systemic arterial blood pressure: Mean arterial blood pressure was measured noninvasively using a tail cuff system (CODA 8 System, Kent Scientific, Torrington, CT, USA). Following 3 cycles of acclimation, systolic, diastolic, and mean pressure measurements were taken from awake mice. 10-20 tail occlusion cycles were averaged for each mouse.

Immunoblotting: Approximately 50 mg of frozen cardiac tissue was pulverized at -78ºC and lysed with buffer (Tris (50 mmol/L), NaCl (150 mmol/L), SDS (0.1%), EDTA (5 mmol/L), β-glycerophosphate (18.5 mmol/L)) containing freshly added protease inhibitor cocktail (Roche, Indianapolis, IN, USA), PMSF (1 mmol/L), sodium fluoride (10 mmol/L), and sodium orthovanadate (250 μmol/L). Tissue samples in lysis buffer were sonicated for 5 seconds with a microtip sonicator 2x, incubated on ice for 30 min, centrifuged at 16,000xg for 25 min, and the supernatant was stored at -80º C. Protein extracts were subjected to SDS-PAGE, and transferred to nitrocellulose membranes. Membranes were blocked with TBS+0.1% Tween-20 (TBS-T) + 5% nonfat milk. Rabbit primary antibodies against UQCRFS1 (RISP, #18443-1-AP, Proteintech Group, Inc., Chicago, IL, USA), rabbit primary antibodies against Meis1/2 (H-80, #sc-25412, Santa Cruz Biotechnology, Inc., Dallas, TX, USA), rabbit primary antibodies against phospho-AMPK-α (Thr172) (#2535, Cell Signaling Technology, Danvers, MA, USA), rabbit primary antibodies against AMPK-α (#2532, Cell Signaling Technology, Danvers, MA, USA), rabbit primary antibodies against myosin light chain kinase 3 (MyLK3; #MYLK3-301AP, FabGennix International Incorporated, Frisco, TX, USA), or rabbit primary antibodies against GAPDH (#2118, Cell Signaling Technology, Danvers, MA, USA) were added to TBS-T+5% milk or BSA and incubated with the membrane overnight at 4º C. Membranes were washed with TBS-T, and species-specific secondary antibodies conjugated with HRP were added for 2 hr. Membranes were washed with TBS-T, stained with ECL reagent (Amersham, Pittsburgh, PA, USA), and analyzed using a Bio-Rad ChemiDoc XRS+ Imaging System with Image Lab software (Bio-Rad, Hercules, CA, USA).

Tissue Histology: Histological staining was performed using the Biocare Intellipath Staining Platform.

- *Hematoxylin/Eosin (H&E), Masson's Trichrome and Periodic Acid Schiff (PAS)*: Staining was performed by the Northwestern University Mouse Histology and Phenotyping Laboratory using a Leica Autostainer XL.

- *Wheat Germ Agglutinin*: Wheat Germ Agglutinin (WGA, #W11261, Life Technologies Corporation, Eugene, OR, USA), used at 1:100, followed by Hoechst staining. Antigen retrieval (paraffin sections): sodium citrate buffer pH6, 110° C for 10 min in BioCare Decloaker. Frozen sections: acetone fixation for 10 min.
- *Ki67/DAPI*: Primary antibody: Abcam #ab16667, used at 1:500, incubated overnight at 4° C. Secondary antibody: Cy3 Jackson Immuno #711-165-152, used at 1:2000, followed by Hoechst staining. Antigen retrieval (paraffin sections): sodium citrate buffer pH6, 110° C for 20 min in BioCare Decloaker.
- *pH3/DAPI*: Primary antibody: Abcam #ab5176, used at 1:1500, incubated overnight at 4° C. Secondary antibody: Cy3 Jackson Immuno #711-165-152, used at 1:2000, followed by Hoechst staining. Antigen retrieval (paraffin sections): sodium citrate buffer pH6, 110° C for 20 min in BioCare Decloaker.
- *Cardiac Myosin Light Chain*: Anti-Myosin Light Chain 2 (#ab79935, Abcam Inc., Cambridge, MA, USA), used at 1:150, incubated overnight at 4° C.
- *8-oxo-dG/DAPI*: Anti-8 Hydroxyguanosine (#ab10802, Abcam Inc., Cambridge, MA, USA), used at 1:1000, incubated overnight at 4° C. Secondary antibody: Cy3 Jackson Immuno #705-165-147, used at 1:2000, alexa488 Jackson Immuno#711545-152, followed by Hoechst staining. Antigen retrieval (paraffin sections): sodium citrate buffer pH*6, 110° C for 20 min in BioCare Decloaker.*
- *EdU/BrdU/DAPI*: Primary antibody: Abcam #Ab1893, used at 1:1000, incubated overnight at 4° C. Secondary antibody: Cy3 Jackson Immuno #713-165-147, used at 1:2000, followed by Hoechst staining. Antigen retrieval: sodium citrate buffer pH6, 110° C for 20 min in BioCare Decloaker.

Histological sample imaging: Tissue sections were imaged using epifluorescence microscopy using a 40x, 1.3 NA Plan Fluor or a 20x, 0.45 NA Plan Fluor objective in an inverted microscope (Nikon, Melville, NY, USA), and imaged with a cooled CCD camera (Photometrics Cascade, Tucson, AZ, USA) controlled by a computer workstation running Metamorph imaging software (Universal Imaging, Bedford Hills, NY, USA). Additional tissue sections were imaged using a Keyence BZ-X800 all-in-one fluorescence microscope (Keyence Corporation of America, Itasca, IL, USA).

Quantitative Analysis of Left Ventricle Histology:

- *Left ventricle wall thickness, cardiomyocyte diameter, and cardiomyocyte cell count*: ImageJ (NIH) was used to quantify the left ventricle wall thickness, cardiomyocyte diameter, and cardiomyocyte count in PAS-stained left ventricular images of RISP WT and RISP KO mice 75 days post-tamoxifen. At multiple random sites, straight-line segments perpendicular to the epicardial surface were drawn from the epicardium to the endocardium for measurement of the ventricle wall thickness. These lines were used to generate image intensity histograms of the PAS staining across the ventricle based on staining intensity as a function of distance across the ventricle. The resulting histogram was used to calculate the diameters of cardiomyocytes intersecting the line, along with the number of cardiomyocytes. Calculated diameters of less than 15 μm were omitted from the measurement, as they were considered to be artifacts of the generated image intensity histogram.

- *Cardiomyocyte cross-sectional area*: The left ventricular cardiomyocyte cross-sectional area of RISP WT and RISP KO mice was quantified 60 days post-tamoxifen using ImageJ (NIH) to analyze images obtained from WGA-stained heart sections. The threshold of the WGA-stained heart slice images was adjusted to demarcate the cytoplasm from the cell membrane of the individual cardiomyocytes. ImageJ particle analysis was then employed to measure the average cardiomyocyte cross-sectional area for the random WGA image section. Only cross-sectional areas of 100-500 μm^2 and a circularity of 0.5-1.0 were included in the calculation; others were considered artifacts from obliquely cut cardiomyocytes.
- *Proliferation assays*: Left ventricular cardiomyocyte proliferation was quantified in the hearts of RISP WT and RISP KO mice 30-, 60-, and 75-days post-tamoxifen using ImageJ (NIH). Random, multiple images were taken of left ventricular heart slices that had been stained with Ki67 and counterstained for DAPI and myosin light chain (MLC-2), stained for pH3 and counterstained for DAPI and myosin light chain (MLC-2), or stained for EdU and counterstained for DAPI and myosin light chain (MLC-2). The threshold Ki67-, pH3-, EdU-, or matching DAPI-stained left ventricular heart slice images was adjusted to demarcate the positively stained nuclei from the rest of the tissue. ImageJ particle analysis was then used to count the number of positively stained nuclei. Particles of less than a certain size (for this study the threshold was set at <25 pixels) were omitted from the quantification as they represented artifacts. Only cardiomyocytes that co-localized Ki67, pH3, or EdU with myosin light chain (MLC-2) were counted.
- *8-oxo-dG assay*: The oxidation of DNA was assessed in 8-oxo-deoxyguanine (8-oxo-dG) immunostained cardiomyocytes that had been counterstained with DAPI. Multiple, random sites were assessed in the left ventricles of RISP WT and RISP KO mice at 60 days post-tamoxifen. ImageJ (NIH) was used to create a mask that limited analysis to the nuclei using the DAPI fluorescence; that was then overlaid on the matching 8-oxo-dG images to restrict the measurement of the 8-oxo-dG intensity to the nuclei of the cardiomyocytes in the left ventricle.

Electron microscopy: Approximately 2 mm^3 sections of the left ventricle were resected from the hearts of 30-, 60-, and 75-day post-tamoxifen RISP WT and RISP KO mice isolated by the isoflurane method described above. Sections were fixed in 0.1 M sodium cacodylate buffer (pH 7.3) containing 2% paraformaldehyde and 2.5% glutaraldehyde, and post-fixed with 2% osmium tetroxide in unbuffered aqueous solution. Next, the sections were rinsed with distilled water and then dehydrated in ascending grades of ethanol. They were transitioned with propylene oxide and then embedded in a resin mixture of Embed 812 kit, which was cured in an oven at 60º C. The resulting left ventricle samples were sectioned on a Leica Ultracut UC6 ultramicrotome. 1 µm thick sections were collected and stained with Toluidine Blue O and used to visually assess the sample quality. Subsequently, 70 nm sections were collected on 200 mesh copper grids and stained with uranyl acetate and Reynolds lead citrate. The resulting thin sections were imaged using a FEI Tecnai Spirit G2 120kV transmission electron microscope (TEM).

Mitochondrial area: Mitochondrial density was assessed in 2900x TEM images from the left ventricles of RISP WT and RISP KO mice at 30-, 60-, and 75-days post-tamoxifen. ImageJ (NIH) was used to trace the circumference of mitochondria in the TEM images and used to calculate the area comprised of mitochondria. This calculation was then used to determine mitochondrial area as a percentage of the total area of the TEM images.

Adenine nucleotide concentrations: Heart concentrations of ATP, ADP, and AMP were determined using high-performance liquid chromatography (HPLC). Snap frozen hearts were pulverized at -78º C and 150 mg of heart tissue was suspended in 300 µL of media (PBS) containing 6.25% 1M $HClO_4$. The $HClO_4$ was removed by mixed phase extraction employing 11.75:13.25 (vol/vol) of tri-*n*-octylamine and Freon 11. ATP, ADP and AMP were measured by HPLC with Shimadzu LC-20A pump and UV-VIS detector with a Supelco LC-18-T column (15 cm x 4.6 cm). The HPLC was run at a flow rate of 1 ml/min with 100% buffer A from 0-5 min, a linear gradient to 95% buffer A/5% buffer B (100% methanol) from 5-6 min, 95% buffer A/5% buffer B from 6-11 min, a linear gradient to 85% buffer A/15% buffer B from 11-13 min, 85% buffer A/15% buffer B from 13-23 min, and a linear gradient to 100% buffer A from 23-30 min. ATP, ADP and AMP eluted as sharp peaks between ~2-6 minutes. Metabolite retention times were verified using purified standards (Sigma Aldrich). Peak areas were normalized to the weight of frozen heart tissue.

RNA-seq: Total RNA was extracted from snap frozen mouse hearts using a QIAshredder kit (QIAGEN, catalog no. 79656) to disrupt the tissue and a RNeasy Mini Kit (QIAGEN, catalog no. 74104) to isolate RNA. RNA concentration and quality were tested using TapeStation 4200 (Agilent), all samples had RIN over 8.0. RNA-seq libraries were prepared using NEB Next RNA Ultra library preparation kit with poly(A) enrichment module from 500 ng of total RNA. RNA-seq-ready libraries were pooled and sequenced on a NextSeq 500 instrument (Illumina) using High Output 75 cycles V2 kit to the average depth of 10M reads per sample. Sequencing data were processed using the modular pipeline Ceto using "Genomics Nodes" and "Analytics Nodes" on Quest, Northwestern University's High-Performance Computing Cluster. Briefly, the data was demultiplexed using bcl2fastq (Illumina), after FastQC assessment, trimming with trimmomatic, the reads were mapped using TopHat2 to the mm10 reference genome and reads were quantified using htseq2. Differential gene expression analysis was performed using the EdgeR statistical package (12). Gene Ontology (GO) analysis was performed using Gorilla (cbl-gorilla.cs.technion.ac.il). Venn diagrams were constructed using the Bioinformatics and Evolutionary Genomics tool: (bioinformatics.psb.ugent.be/webtools/Venn).

Metabolomics: Whole hearts from 60-day post-tamoxifen RISP WT and RISP KO mice were snap frozen as described above and shipped to Metabolon, Inc. (Durham, NC, USA) on dry ice to assess the metabolomic consequences of decreased RISP expression in cardiomyocytes. Samples were stored at -80º C until processed. Samples were prepared using an automated MicroLab STAR® system (Hamilton Company). Recovery standards were added prior to the first step in the extraction process for QC

purposes. Proteins were precipitated with methanol under vigorous shaking for 2 min (Glen Mills GenoGrinder 2000) followed by centrifugation. The resulting extract was divided into five fractions: two for analysis by two separate reverse phase (RP)/UPLC-MS/MS methods with positive ion mode electrospray ionization (ESI), one for analysis by RP/UPLC-MS/MS with negative ion mode ESI, one for analysis by HILIC/UPLC-MS/MS with negative ion mode ESI, and one sample was reserved for backup. Samples were placed briefly on a TurboVap® (Zymark) to remove the organic solvent. The sample extracts were stored overnight under nitrogen before preparation for analysis.

Controls were analyzed in concert with the experimental samples: a pooled matrix sample generated by taking a small volume of each experimental sample (or alternatively, use of a pool of well-characterized human plasma) served as a technical replicate throughout the data set; extracted water samples served as process blanks; and a cocktail of QC standards that were chosen not to interfere with the measurement of endogenous compounds were spiked into every analyzed sample, allowed instrument performance monitoring and aided chromatographic alignment. Instrument variability was determined by calculating the median relative standard deviation (RSD) for the standards that were added to each sample prior to injection into the mass spectrometers. Overall process variability was determined by calculating the median RSD for all endogenous metabolites (i.e., non-instrument standards) present in 100% of the pooled matrix samples. Experimental samples were randomized across the platform run with QC samples spaced evenly among the injections.

All methods utilized a Waters ACQUITY ultra-performance liquid chromatography (UPLC) and a Thermo Scientific Q-Exactive high resolution/accurate mass spectrometer interfaced with a heated electrospray ionization (HESI-II) source and Orbitrap mass analyzer operated at 35,000 mass resolution. The sample extract was dried then reconstituted in solvents compatible to each of the four methods. Each reconstitution solvent contained a series of standards at fixed concentrations to ensure injection and chromatographic consistency. One aliquot was analyzed using acidic positive ion conditions, chromatographically optimized for more hydrophilic compounds. In this method, the extract was gradient eluted from a C18 column (Waters UPLC BEH C18-2.1x100 mm, 1.7 µm) using water and methanol, containing 0.05% perfluoropentanoic acid (PFPA) and 0.1% formic acid (FA). Another aliquot was also analyzed using acidic positive ion conditions; however, it was chromatographically optimized for more hydrophobic compounds. In this method, the extract was gradient eluted from the same afore mentioned C18 column using methanol, acetonitrile, water, 0.05% PFPA and 0.01% FA and was operated at an overall higher organic content. Another aliquot was analyzed using basic negative ion optimized conditions using a separate dedicated C18 column. The basic extracts were gradient eluted from the column using methanol and water, however with 6.5mM Ammonium Bicarbonate at pH 8. The fourth aliquot was analyzed via negative ionization following elution from a HILIC column (Waters UPLC BEH Amide 2.1x150 mm, 1.7 µm) using a gradient consisting of water and acetonitrile with 10mM Ammonium Formate, pH 10.8. The MS analysis alternated between MS and data-dependent MS^n scans using dynamic exclusion. The scan range varied slighted between methods but covered 70-1000 m/z. Raw data files are archived and extracted as described below.

The informatics system consisted of four major components, the Laboratory Information Management System (LIMS), the data extraction and peak-identification software, data processing tools for QC and compound identification, and a collection of information interpretation and visualization tools for use by data analysts. The hardware and software foundations for these informatics components were the LAN backbone, and a database server running Oracle 10.2.0.1 Enterprise Edition.

Raw data was extracted, peak-identified and QC processed. Compounds were identified by comparison to library entries of purified standards or recurrent unknown entities. Biochemical identifications were based on three criteria: retention index within a narrow RI window of the proposed identification, accurate mass match to the library +/- 10 ppm, and the MS/MS forward and reverse scores between the experimental data and authentic standards. The MS/MS scores are based on a comparison of the ions present in the experimental spectrum to the ions present in the library spectrum.

Peaks were quantified using area-under-the-curve. For studies spanning multiple days, a data normalization step was performed to correct variation resulting from instrument inter-day tuning differences. Essentially, each compound was corrected in run-day blocks by registering the medians to equal one (1.00) and normalizing each data point proportionately (termed “block correction”). Welch’s two-sample *t*-test was used to test whether two unknown means were different from two independent populations.

Random forest analysis: This is a supervised classification technique based on an ensemble of decision trees (13). For a given decision tree, a random subset of the data with identifying true class information was selected to build the tree (“bootstrap sample”), and then the remaining data, the “out-of-bag” (OOB) variables, were passed down the tree to obtain a class prediction for each sample. This process was repeated thousands of times to produce the forest. The final classification of each sample is determined by computing the class prediction frequency (“votes”) for the OOB variables over the whole forest. This method is unbiased since the prediction for each sample is based on trees built from a subset of samples that do not include that sample. Random forest makes no parametric assumptions, variable selection is not needed, it does not overfit, and it is invariant to transformation. To determine which biochemicals make the largest contribution to the classification, a “variable importance” measure is computed. The “Mean Decrease Accuracy” (MDA) was used as this metric. The MDA is determined by randomly permuting a variable, running the observed values through the trees, and then reassessing the prediction accuracy. If a variable is not important, then this procedure will have little change in the accuracy of the class prediction (permuting random noise will give random noise). By contrast, if a variable is important to the classification, the prediction accuracy will drop after such a permutation, which is recorded as the MDA. Thus, the random forest analysis provides an “importance” rank ordering of biochemicals.

Modified reduced representation bisulfite sequencing: Measurement and analysis of DNA methylation was performed as previously described using a modified reduced representation bisulfite sequencing (mRRBS) procedure (14-18). Briefly, genomic DNA was isolated from myocardial tissue using a QIAshredder kit (QIAGEN, catalog no.

79656) to disrupt the tissue and a RNeasy Mini Kit (QIAGEN, catalog no. 74104) to isolate RNA. Endonuclease digestion, size selection, bisulfite conversion, and library preparation were performed as previously described on ~ 250 ng of input genomic DNA. Bisulfite conversion efficiency averaged 99.3% (SD 0.04%) as estimated by the measured percentage of unmethylated CpGs in λ-bacteriophage DNA (New England BioLabs N3013S) added at a 1:200 mass ratio to each sample. Six libraries per run were multiplexed for single-end sequencing using a NextSeq 500/550 V2 High Output reagent kit (1 x 75 cycles) on an Illumina NextSeq 500 sequencer. Demultiplexing, trimming, alignment to the GRCm38/mm10 reference genome, and methylation calling were performed as previously described in the references above. Quantification, statistical hypothesis testing for differentially methylated cytosines, application of a methylation difference-filtering algorithm, and functional enrichment analysis were performed using the DSS v2.30.1 R/Bioconductor package,(19) SeqMonk v1.45.0 platform,(20) and Molecular Signatures Database (MSigDB) (The Broad Institute) (21) as previously described in the references above. Computational analysis was performed using “Genomics Nodes” and “Analytics Nodes” on Quest, Northwestern University’s High-Performance Computing Cluster.

RNA-Seq analysis. Heart tissue was shredded to disrupt the cells (QIAshredder catalog no. 79656, QIAGEN) allowing total RNA to be isolated (RNeasy mini kit, catalog no. 74104, QIAGEN). RNA quality and concentration were assessed (TapeStation 4200, Agilent). All samples yielded RIN > 8.0. RNA-seq libraries were generated (NEB Next RNA Ultra library preparation kit with poly(A) enrichment module) from 500 ng of total RNA. RNA-seq- libraries were pooled and sequenced on a NextSeq 500 instrument (Illumina) using the High Output 75 cycles V2 kit to an average depth of 10M reads per sample. Sequencing data were processed using the modular pipeline Ceto using “Genomics Nodes” and “Analytics Nodes” on Quest, Northwestern University’s High-Performance Computing Cluster. The data were demultiplexed using bcl2fastq (Illumina). Following FastQC assessment and trimming with trimmomatic, the reads were mapped using TopHat2 to the mm10 reference genome and reads were quantified using htseq2. Differential gene expression analysis was performed using the EdgeR statistical package (12).

Statistics: Statistics were performed using GraphPad Prism (GraphPad Software, Boston, MA, USA). Studies assessing differences between RISP WT and RISP KO hearts were analyzed using an unpaired, 2-tailed T-test (U-2t-T-test). Studies assessing differences between RISP WT and RISP KO hearts as a function of time were analyzed using a Two-way ANOVA with a Sidak’s multiple comparison test to evaluate significant differences between groups (2W-ANOVA-Sidak’s). Studies assessing differences in experimental intervention, such has menadione/ascorbic acid were analyzed using an ANOVA with a Tukey’s multiple comparison test to evaluate differences between groups (ANOVA-Tukey’s). Studies assessing differences in transcriptomic responses between RISP WT and RISP KO hearts were analyzed using the EdgeR statistical package (12). Metabolomic studies performed by Metabolon, Inc. (Durham, NC, USA) assessing metabolite differences between RISP WT and RISP KO hearts were analyzed using a 2-tailed, Welch’s Two-sample T-test (2t-W-ts-T-test); Array Studio statistical software

(omicsoftdocs.github.io). Studies assessing epigenetic analysis of RISP WT and RISP KO hearts employed a Kolmogorov-Smirnov test, a generalized linear model and ANOVA-like test, or a beta-binomial regression model with an arcsine link function fitted using the generalized least square method and Wald-test to determine differences in DNA CpG methylation and differentially expressed genes. To control for experimental differences in the responses, experimental studies and control studies were always carried out on the same day. Statistical significance was set at $p<0.05$.

References

1. Waypa GB, Marks JD, Guzy RD, Mungai PT, Schriewer JM, Dokic D, et al. Superoxide generated at mitochondrial complex III triggers acute responses to hypoxia in the pulmonary circulation. *Am J Respir Crit Care Med.* 2013;187(4):424-32.
2. Sohal DS, Nghiem M, Crackower MA, Witt SA, Kimball TR, Tymitz KM, et al. Temporally regulated and tissue-specific gene manipulations in the adult and embryonic heart using a tamoxifen-inducible Cre protein. *Circ Res.* 2001;89(1):20-5.
3. Muzumdar MD, Tasic B, Miyamichi K, Li L, and Luo L. A global double-fluorescent Cre reporter mouse. *Genesis.* 2007;45(9):593-605.
4. Li J, Fettiplace M, Chen SJ, Steinhorn B, Shao Z, Zhu X, et al. Lipid emulsion rapidly restores contractility in stunned mouse cardiomyocytes: a comparison with therapeutic hypothermia. *Crit Care Med.* 2014;42(12):e734-40.
5. Ackers-Johnson M, Li PY, Holmes AP, O'Brien SM, Pavlovic D, and Foo RS. A Simplified, Langendorff-Free Method for Concomitant Isolation of Viable Cardiac Myocytes and Nonmyocytes From the Adult Mouse Heart. *Circ Res.* 2016;119(8):909-20.
6. Nakada Y, Canseco DC, Thet S, Abdisalaam S, Asaithamby A, Santos CX, et al. Hypoxia induces heart regeneration in adult mice. *Nature.* 2017;541(7636):222-7.
7. Lugrin J, Parapanov R, Krueger T, and Liaudet L. Murine Myocardial Infarction Model using Permanent Ligation of Left Anterior Descending Coronary Artery. *J Vis Exp.* 2019(150).
8. Zhang S, Yeap XY, DeBerge M, Naresh NK, Wang K, Jiang Z, et al. Acute CD47 Blockade During Ischemic Myocardial Reperfusion Enhances Phagocytosis-Associated Cardiac Repair. *JACC Basic Transl Sci.* 2017;2(4):386-97.
9. Kuznetsov AV, Veksler V, Gellerich FN, Saks V, Margreiter R, and Kunz WS. Analysis of mitochondrial function in situ in permeabilized muscle fibers, tissues and cells. *Nat Protoc.* 2008;3(6):965-76.
10. Dooley CT, Dore TM, Hanson GT, Jackson WC, Remington SJ, and Tsien RY. Imaging dynamic redox changes in mammalian cells with green fluorescent protein indicators. *J Biol Chem.* 2004;279(21):22284-93.
11. Hanson GT, Aggeler R, Oglesbee D, Cannon M, Capaldi RA, Tsien RY, et al. Investigating mitochondrial redox potential with redox-sensitive green fluorescent protein indicators. *J Biol Chem.* 2004;279(13):13044-53.

12. Robinson MD, McCarthy DJ, and Smyth GK. edgeR: a Bioconductor package for differential expression analysis of digital gene expression data. *Bioinformatics.* 2010;26(1):139-40.
13. Breiman L. Random forests. *Mach Learn.* 2001;45(1):5-32.
14. McGrath-Morrow SA, Ndeh R, Helmin KA, Chen SY, Anekalla KR, Abdala-Valencia H, et al. DNA methylation regulates the neonatal CD4(+) T-cell response to pneumonia in mice. *J Biol Chem.* 2018;293(30):11772-83.
15. Walter JM, Helmin KA, Abdala-Valencia H, Wunderink RG, and Singer BD. Multidimensional assessment of alveolar T cells in critically ill patients. *JCI Insight.* 2018;3(17):e123287.
16. Helmin KA, Morales-Nebreda L, Torres Acosta MA, Anekalla KR, Chen SY, Abdala-Valencia H, et al. Maintenance DNA methylation is essential for regulatory T cell development and stability of suppressive function. *J Clin Invest.* 2020;130(12):6571-87.
17. Morales-Nebreda L, Helmin KA, Torres Acosta MA, Markov NS, Hu JY, Joudi AM, et al. Aging imparts cell-autonomous dysfunction to regulatory T cells during recovery from influenza pneumonia. *JCI Insight.* 2021;6(6).
18. Singer BD. A Practical Guide to the Measurement and Analysis of DNA Methylation. *Am J Respir Cell Mol Biol.* 2019;61(4):417-28.
19. Feng H, Conneely KN, and Wu H. A Bayesian hierarchical model to detect differentially methylated loci from single nucleotide resolution sequencing data. *Nucleic Acids Res.* 2014;42(8):e69.
20. Andrews S. SeqMonk. https://www.bioinformatics.babraham.ac.uk/projects/seqmonk/. Accessed 19 Apr 2018.
21. Subramanian A, Tamayo P, Mootha VK, Mukherjee S, Ebert BL, Gillette MA, et al. Gene set enrichment analysis: a knowledge-based approach for interpreting genome-wide expression profiles. *Proc Natl Acad Sci U S A.* 2005;102(43):15545-50.
