# Supplementary material for: Mitochondria regulate proliferation in adult cardiac myocytes
Source: J Clin Invest. 2024 May 9;134(13):e165482. doi: 10.1172/JCI165482 (PMC11213516; doi:10.1172/JCI165482)
Supplement: Unedited blot and gel images [file jci-134-165482-s056.pdf]

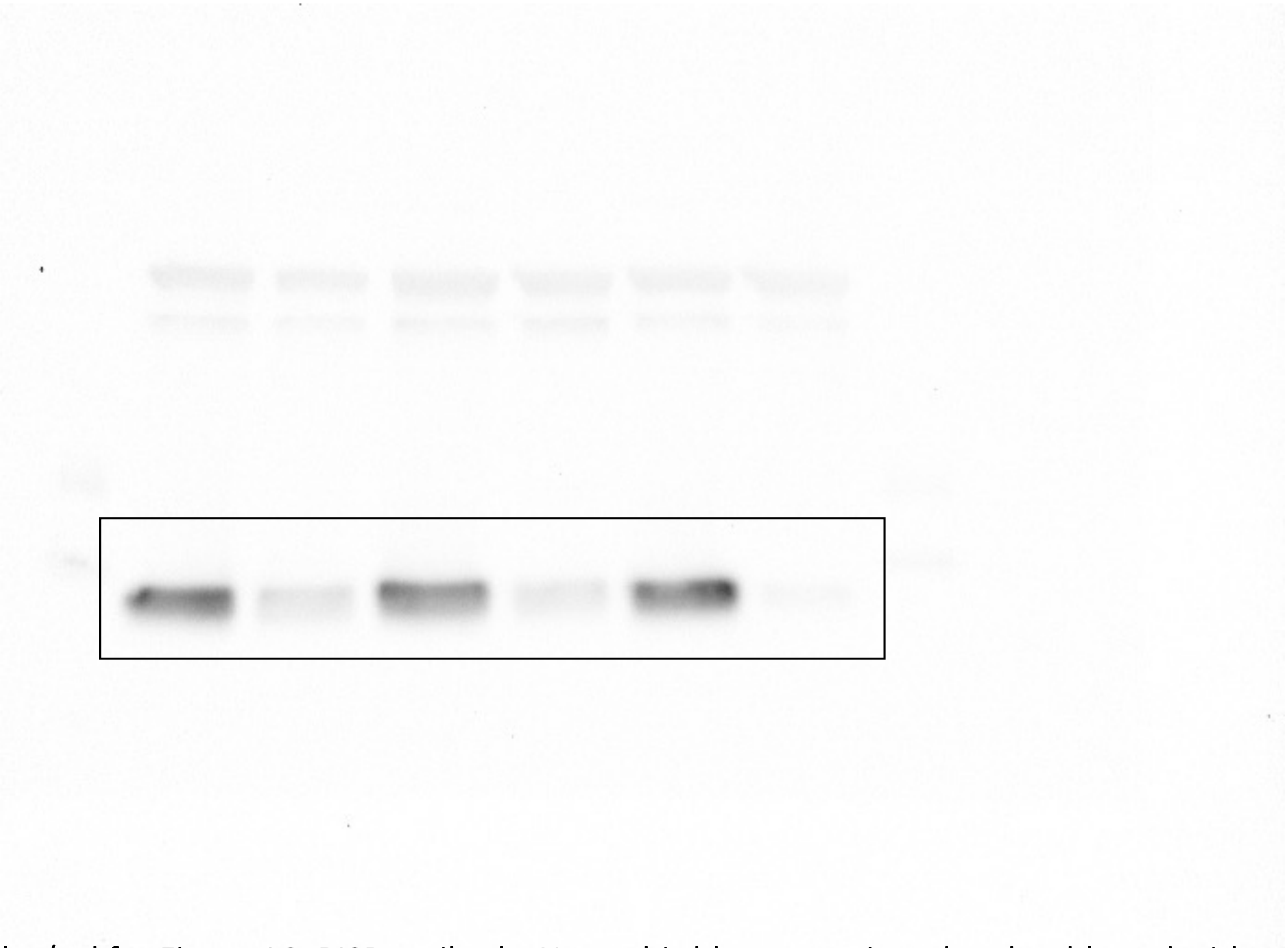

Full unedited blot/gel for Figure 1C: RISP antibody. Note: this blot was stripped and re-blotted with a Meis 1/2 antibody for Supplement Figure S8A.

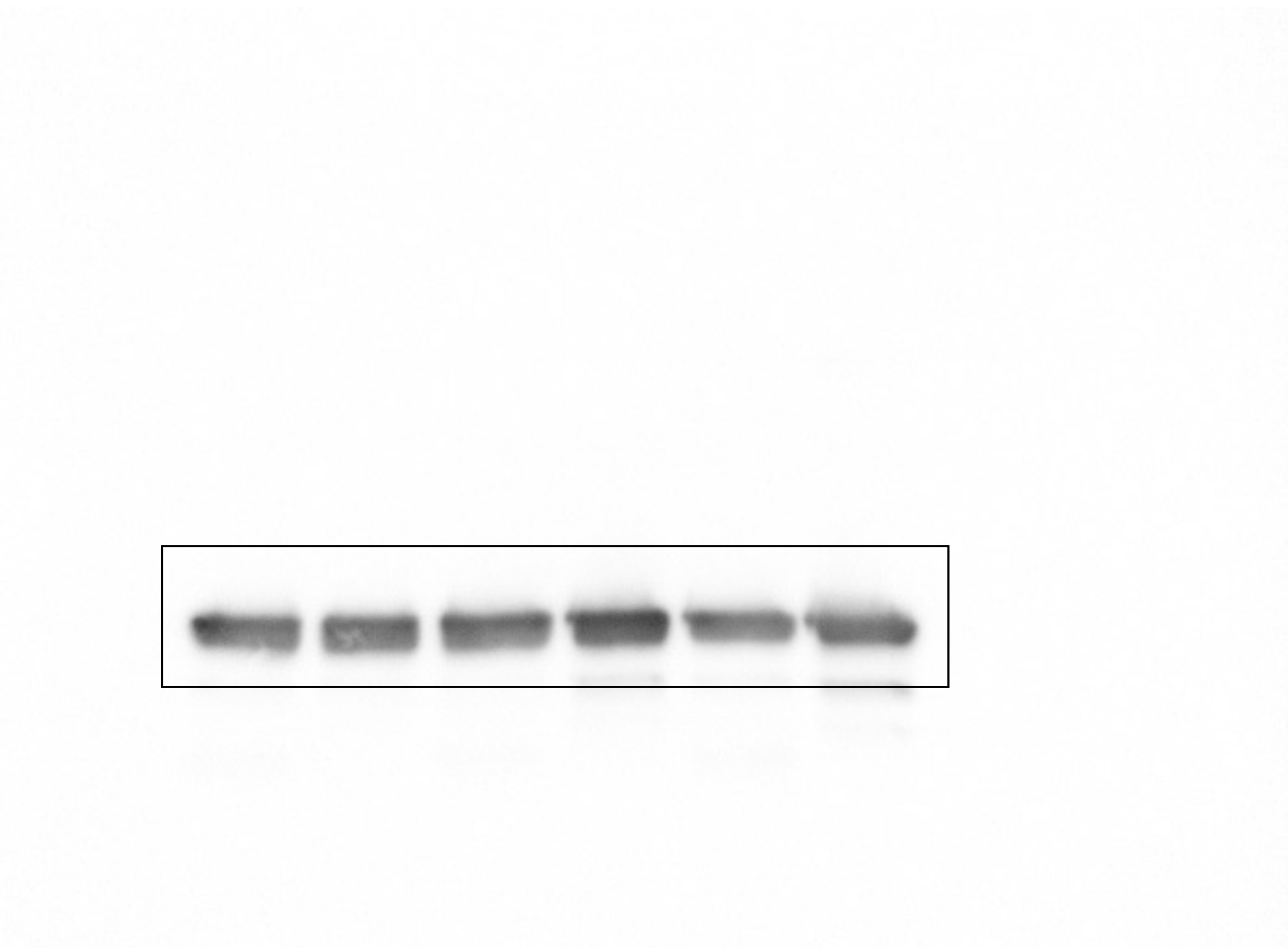

Full unedited blot/gel for Figure 1C: GAPDH antibody. Note: this is the same GAPDH blot in Supplement Figure S8A.

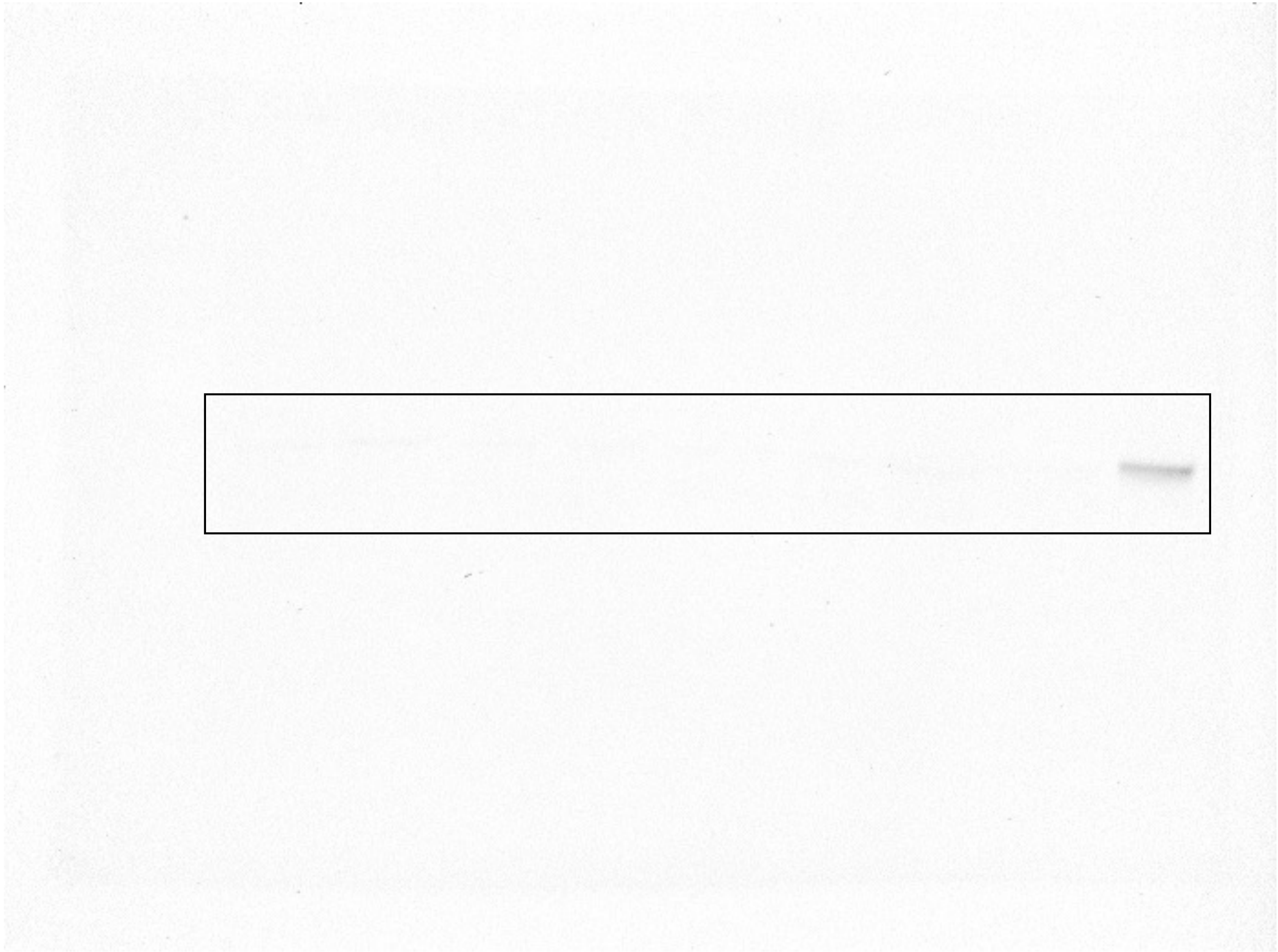

Full unedited blot/gel for Figure 1J: P-AMPK antibody.

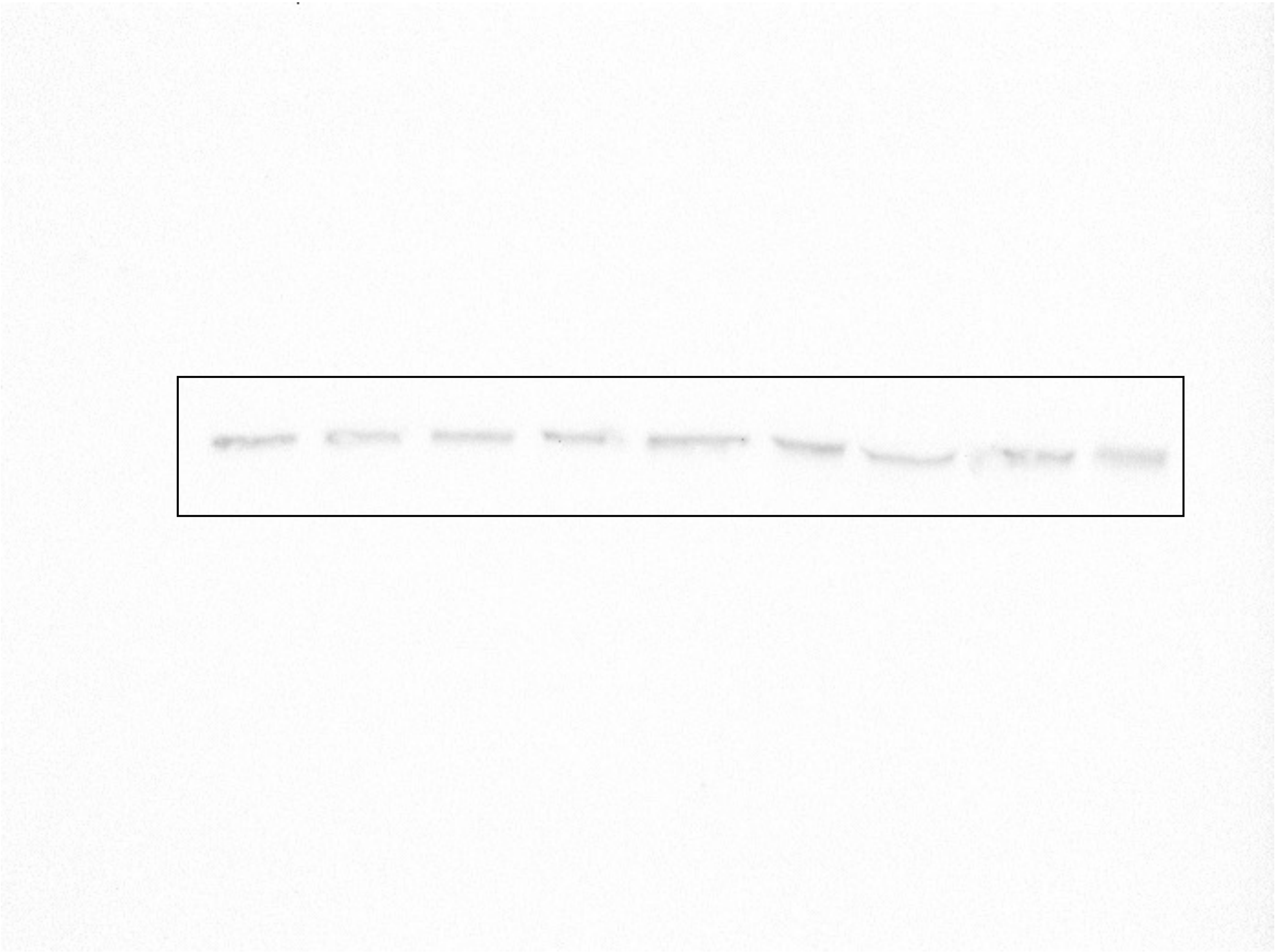

Full unedited blot/gel for Figure 1J: AMPK antibody.

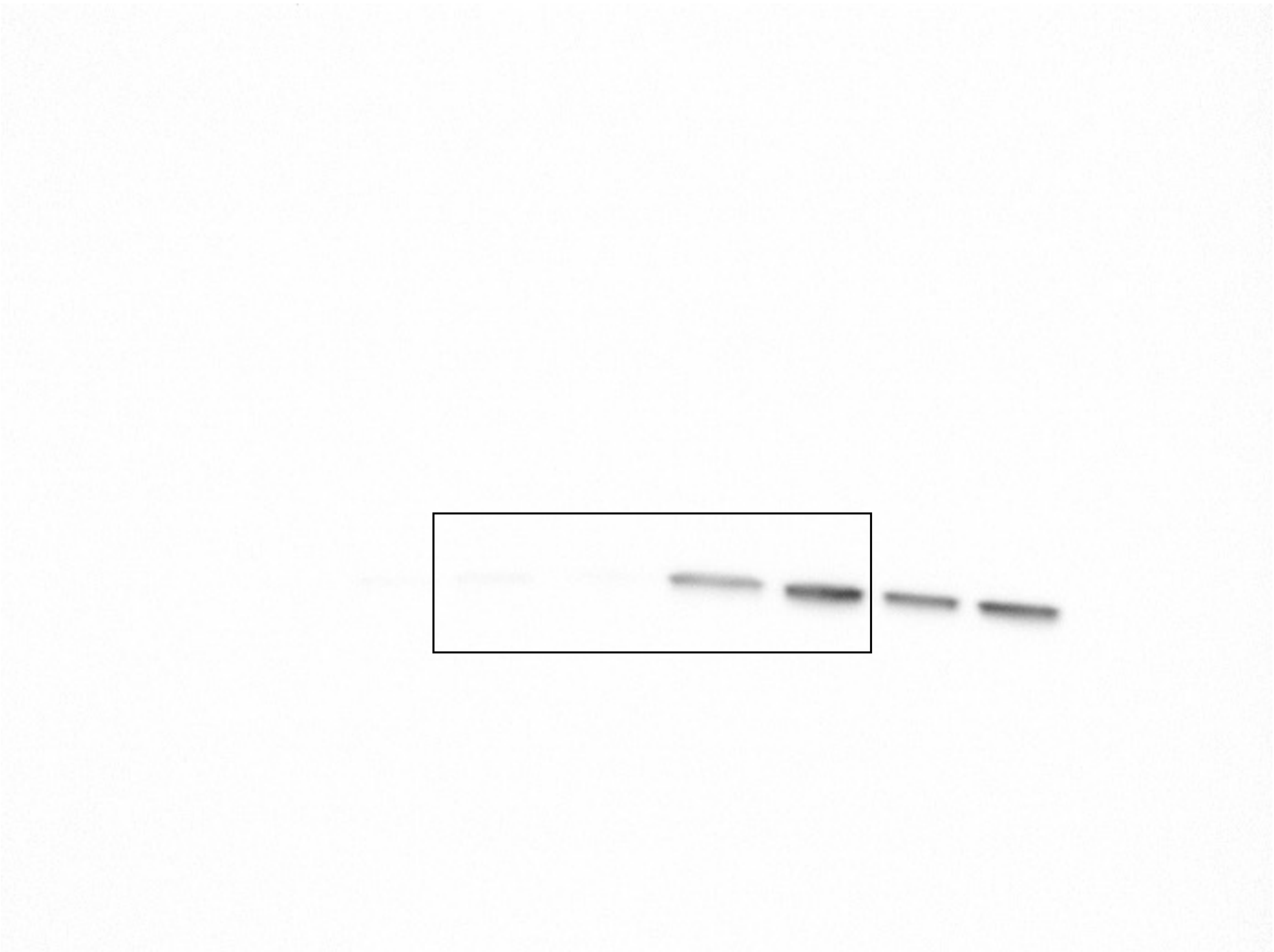

Full unedited blot/gel for Figure 6A: P-S6 antibody.

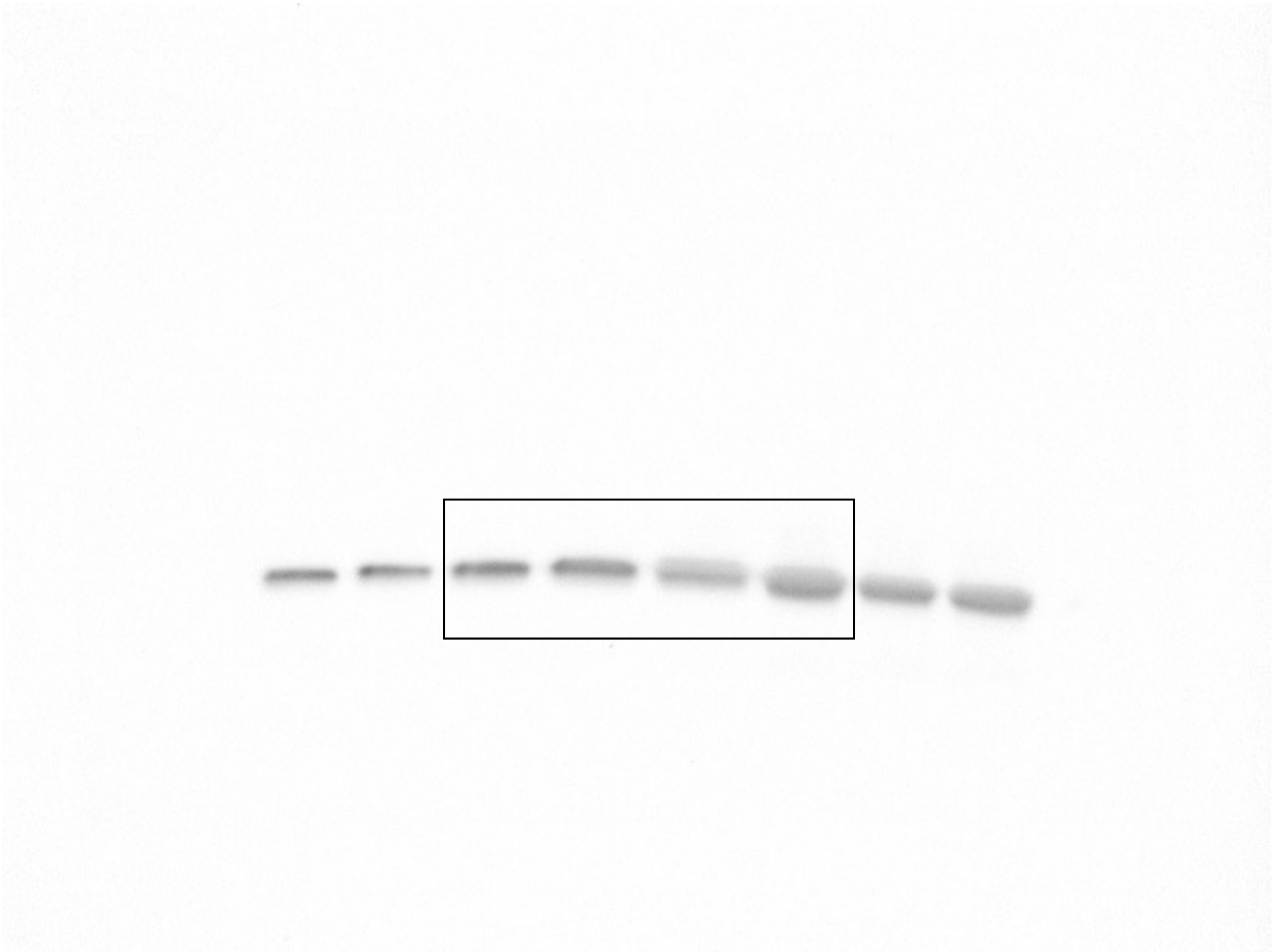

Full unedited blot/gel for Figure 6A: 6A antibody.

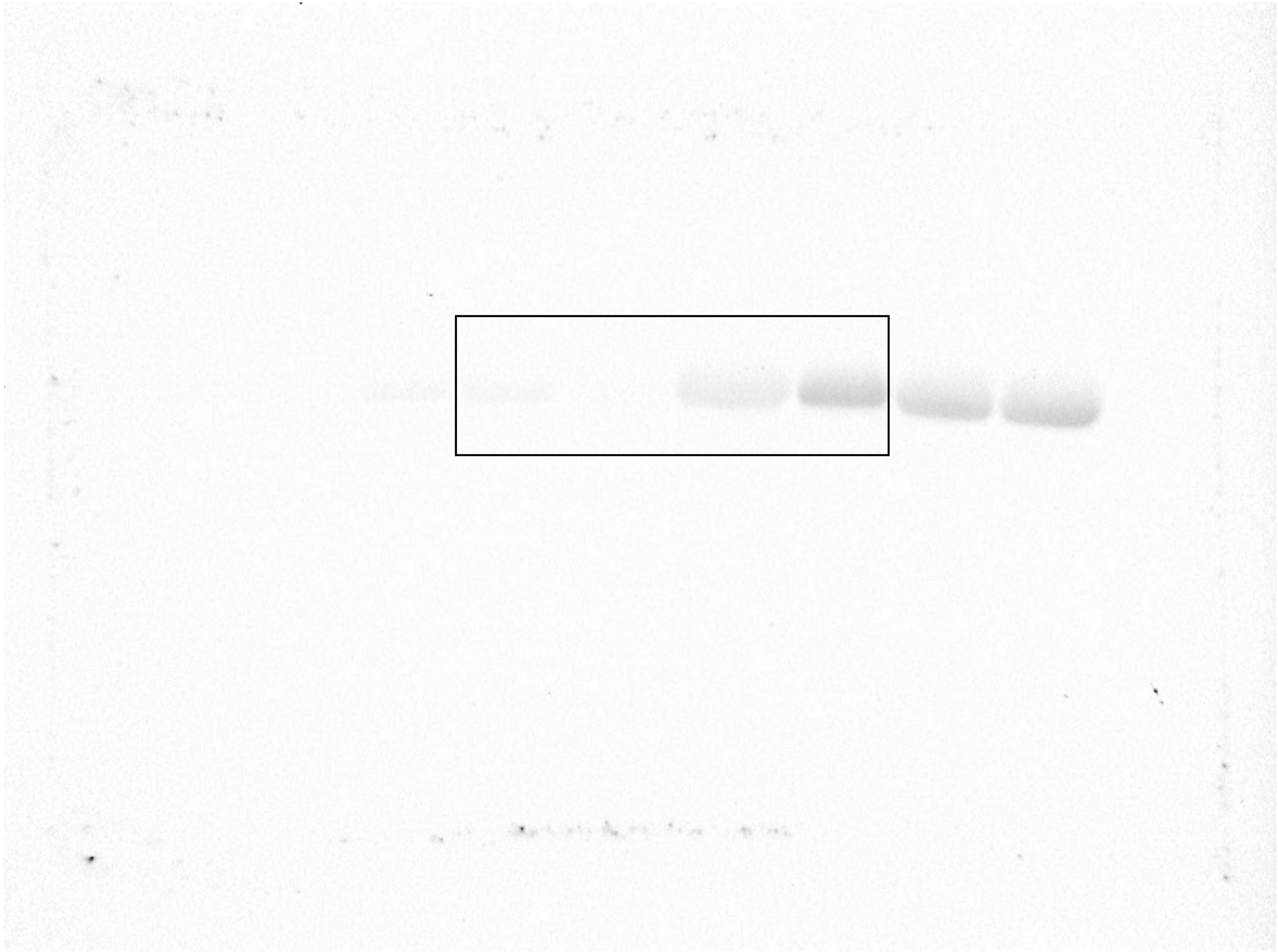

Full unedited blot/gel for Figure 6C: P-S6 Kinase antibody.

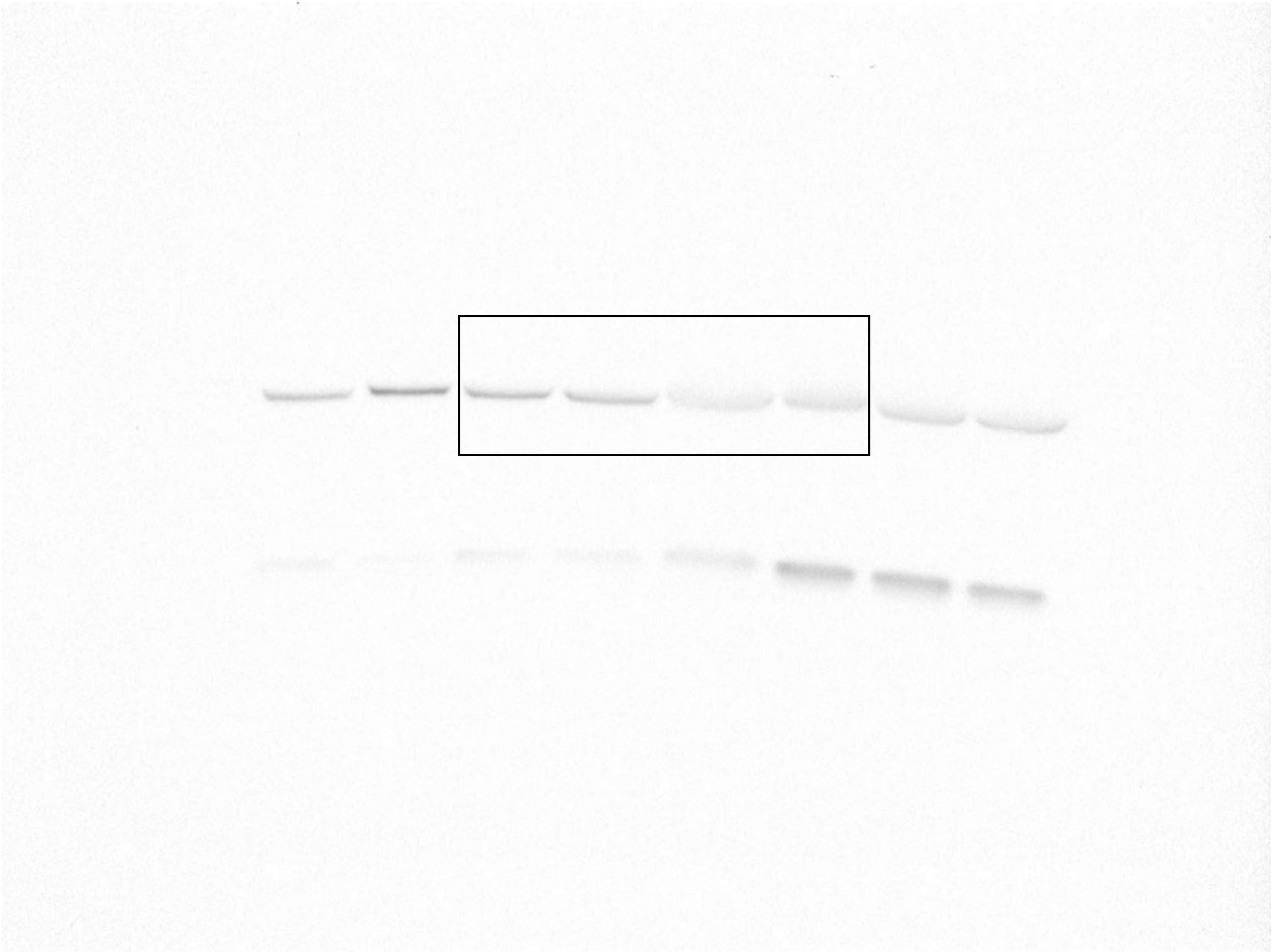

Full unedited blot/gel for Figure 6C: S6 Kinase antibody.

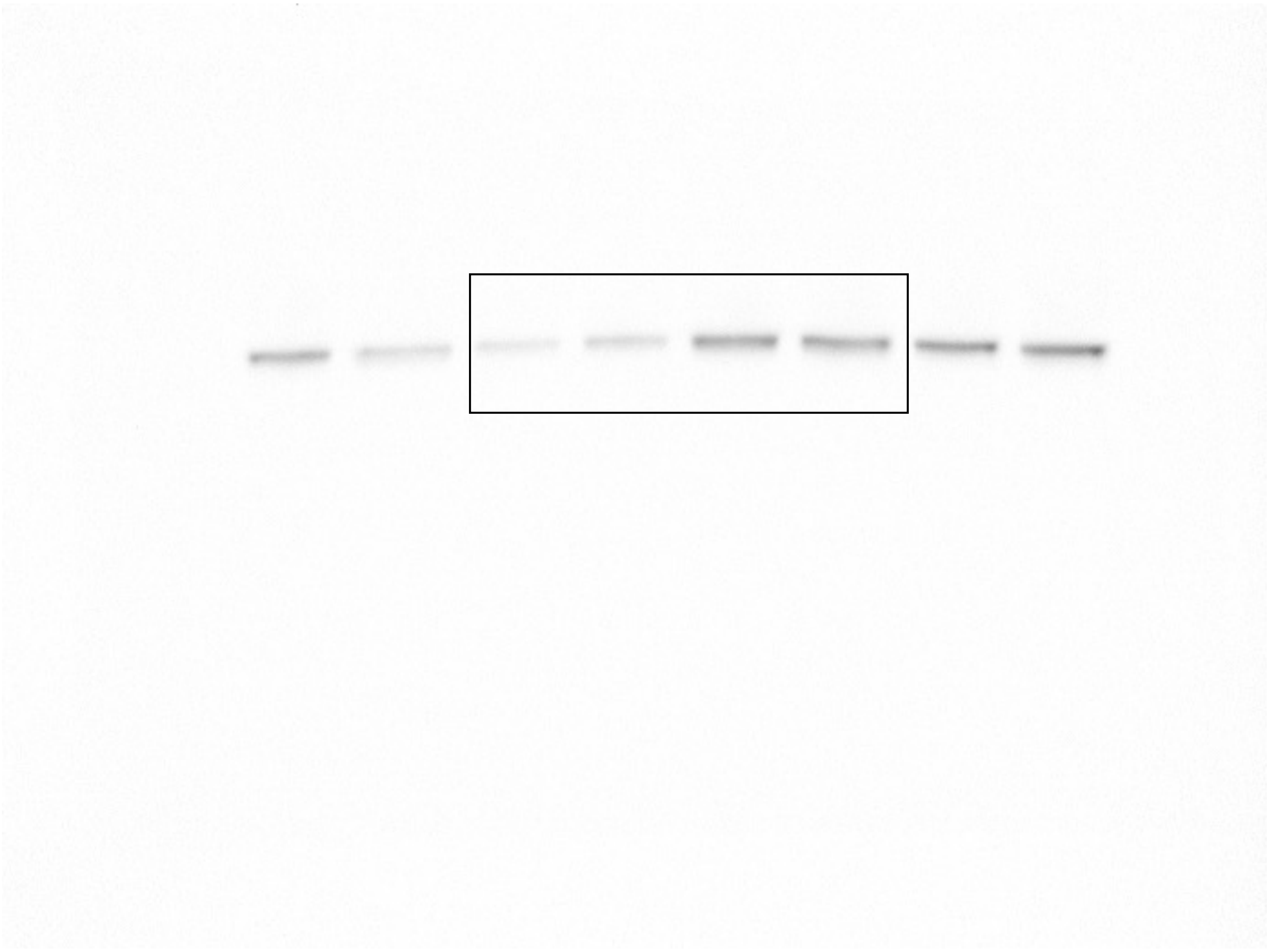

Full unedited blot/gel for Figure 6E: P-AKT antibody.

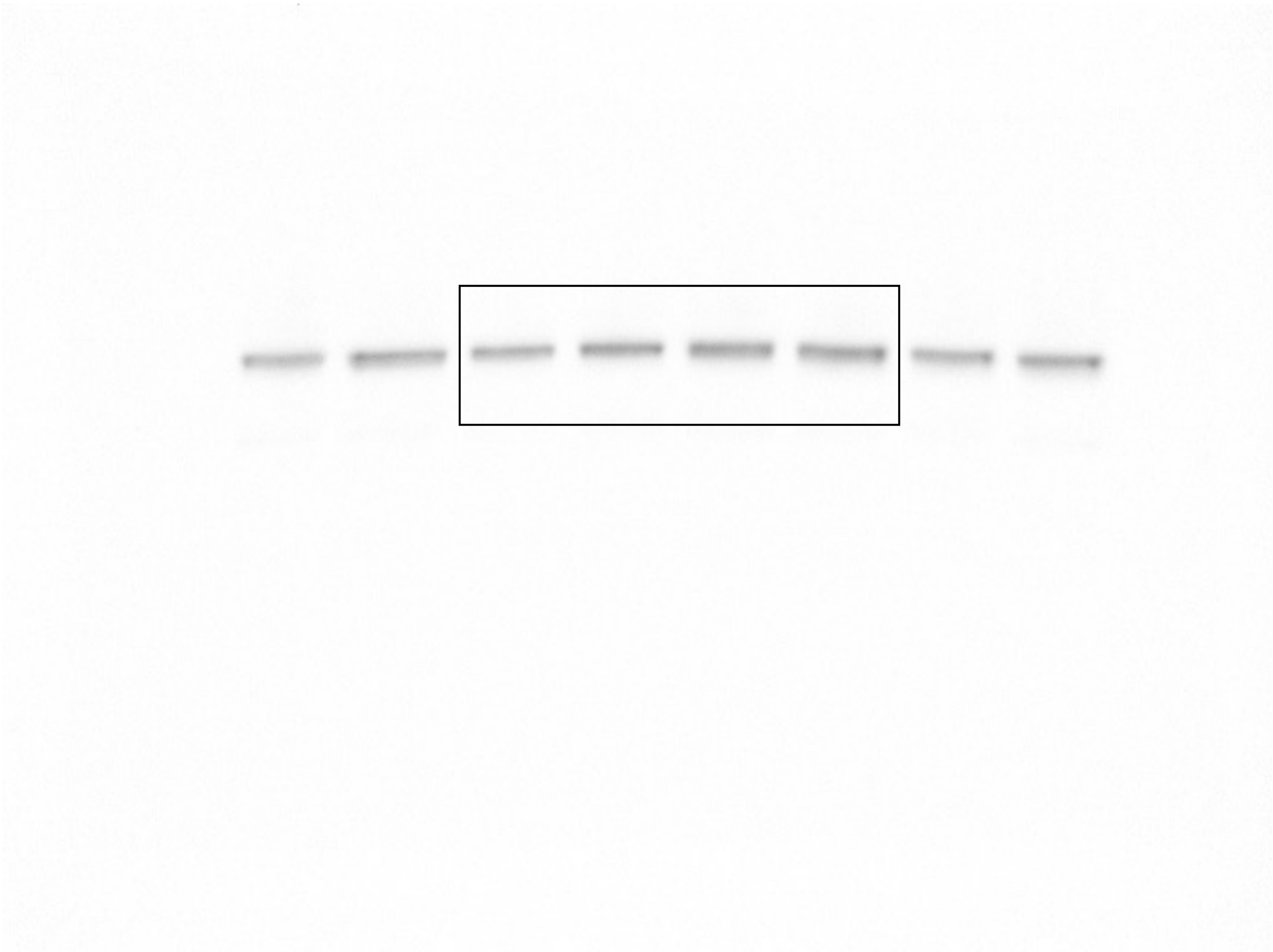

Full unedited blot/gel for Figure 6E: AKT antibody.

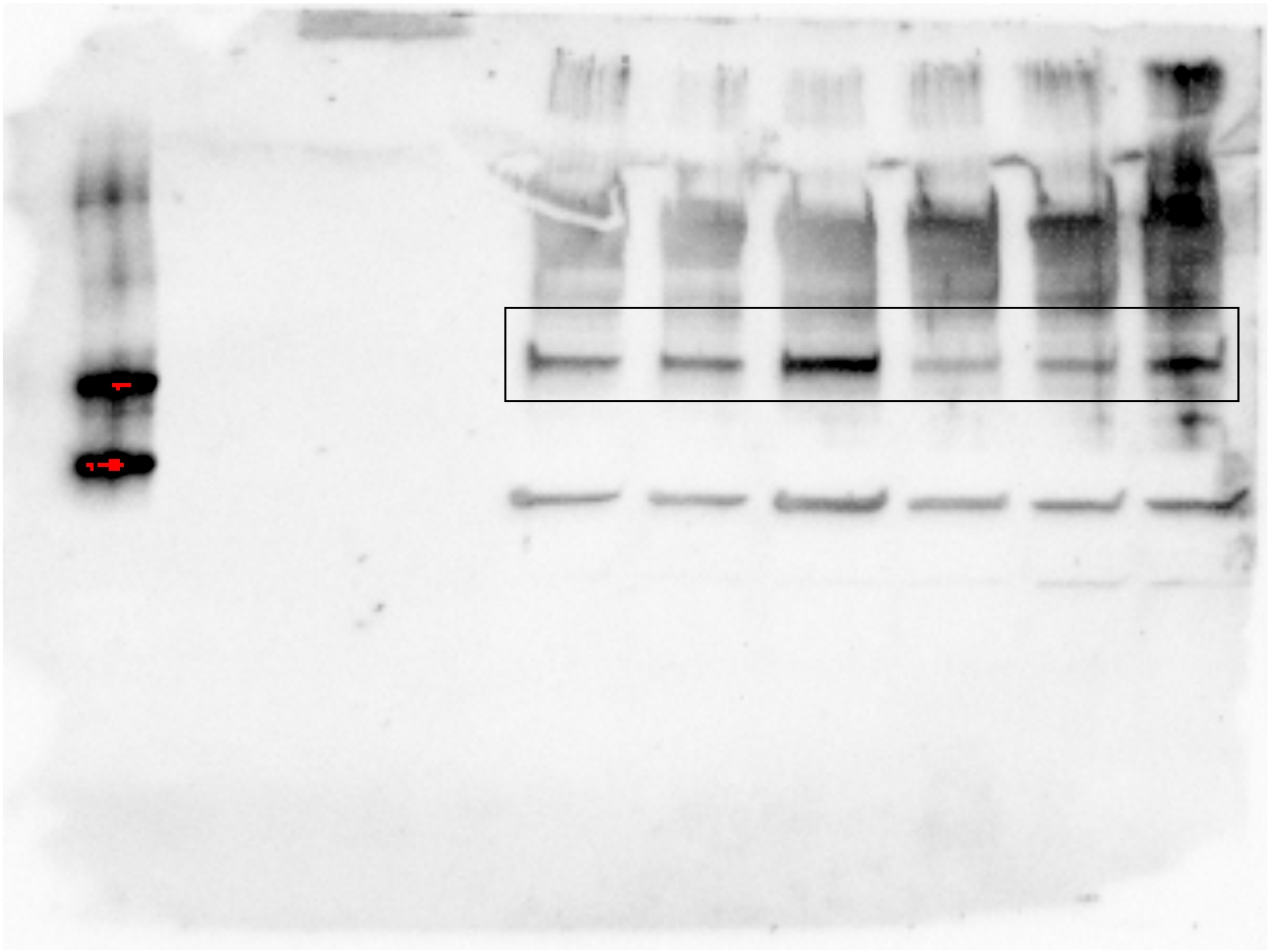

Full unedited blot/gel for Figure 8B: MyLK3 antibody.

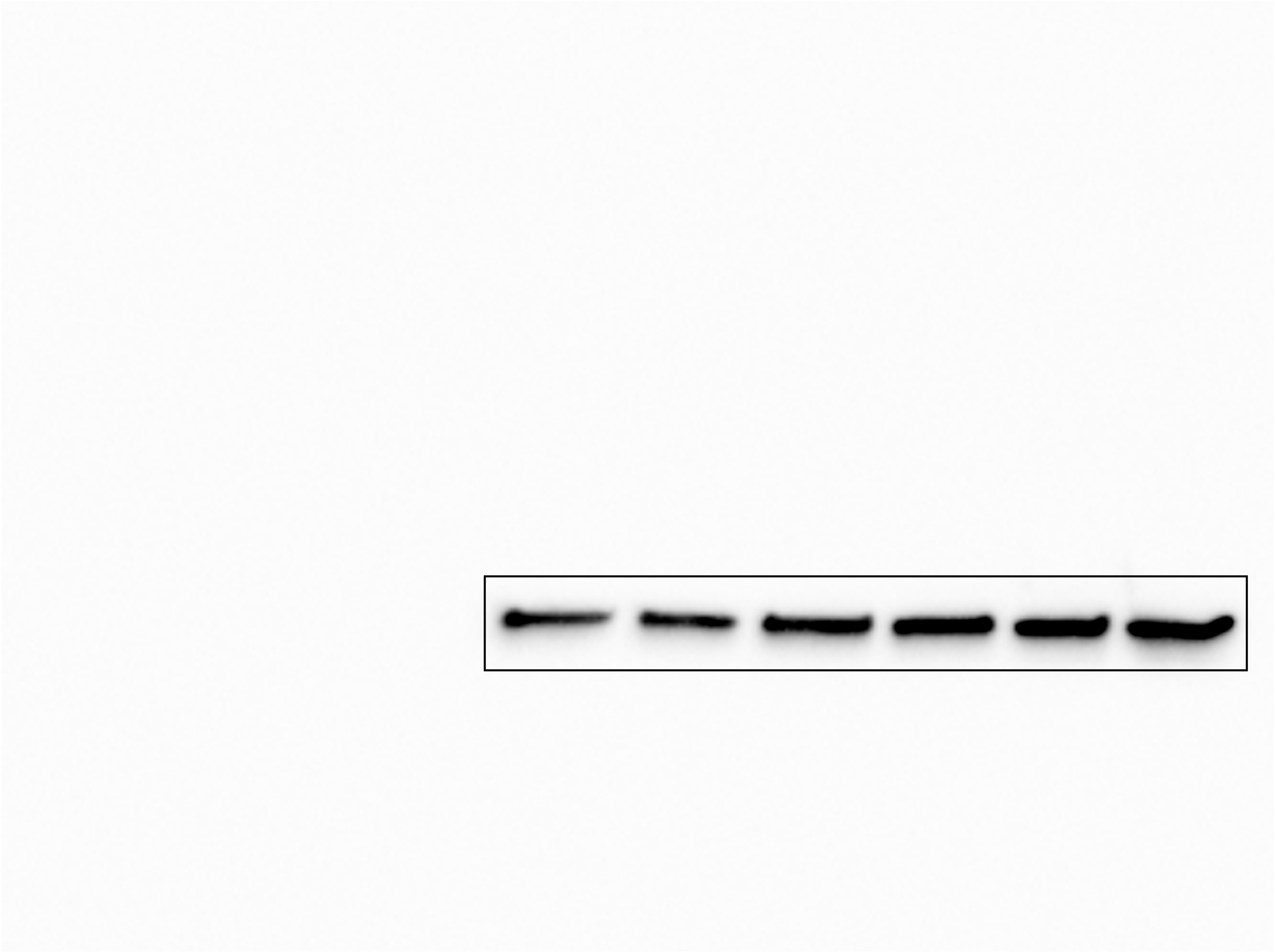

Full unedited blot/gel for Figure 8B: GAPDH antibody.

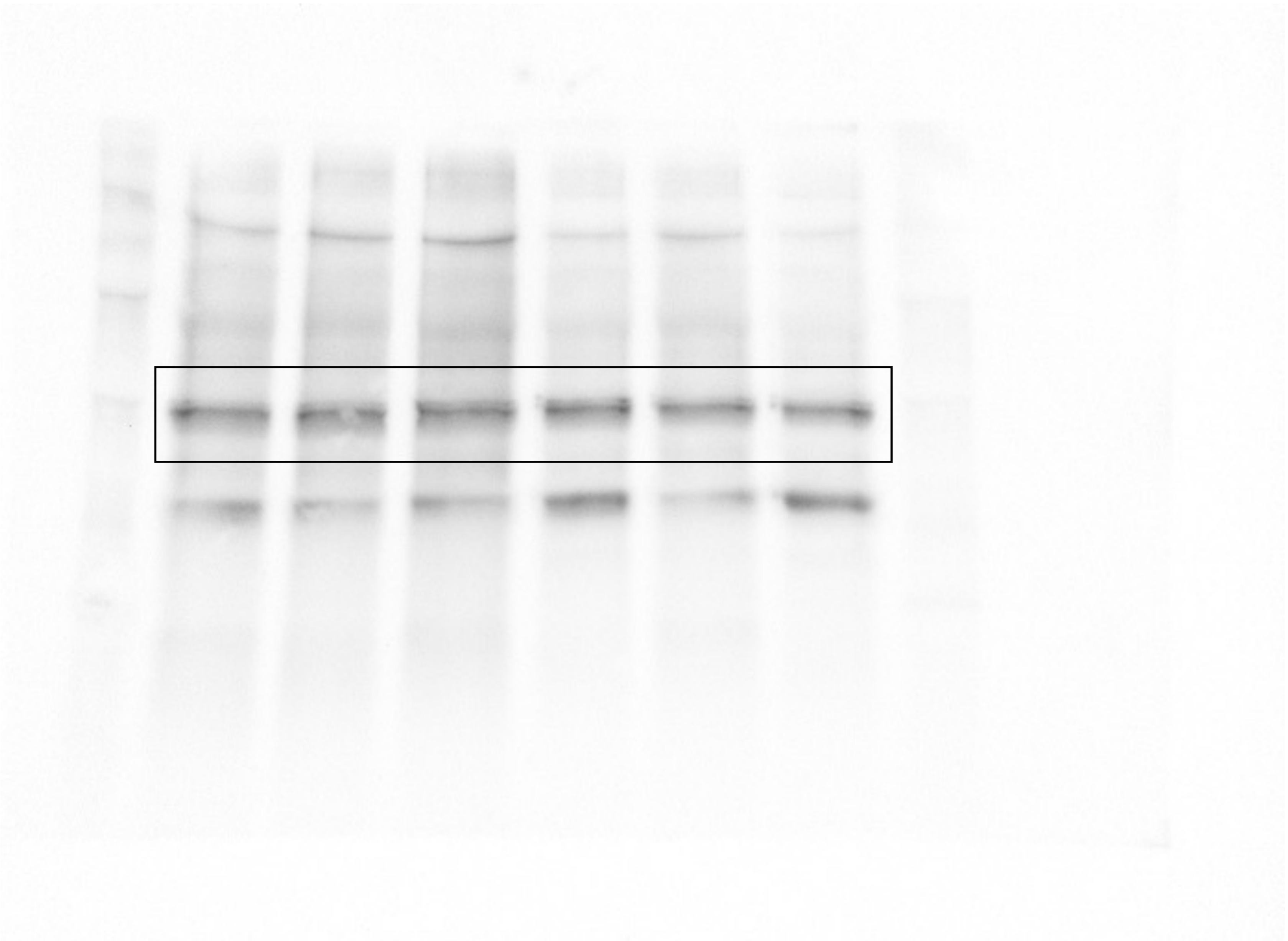

Full unedited blot/gel for Supplement Figure S8A: Meis 1/2 antibody. Note: this is the same blot as Figure 1C that was stripped and re-blotted with a Meis 1/2 antibody.

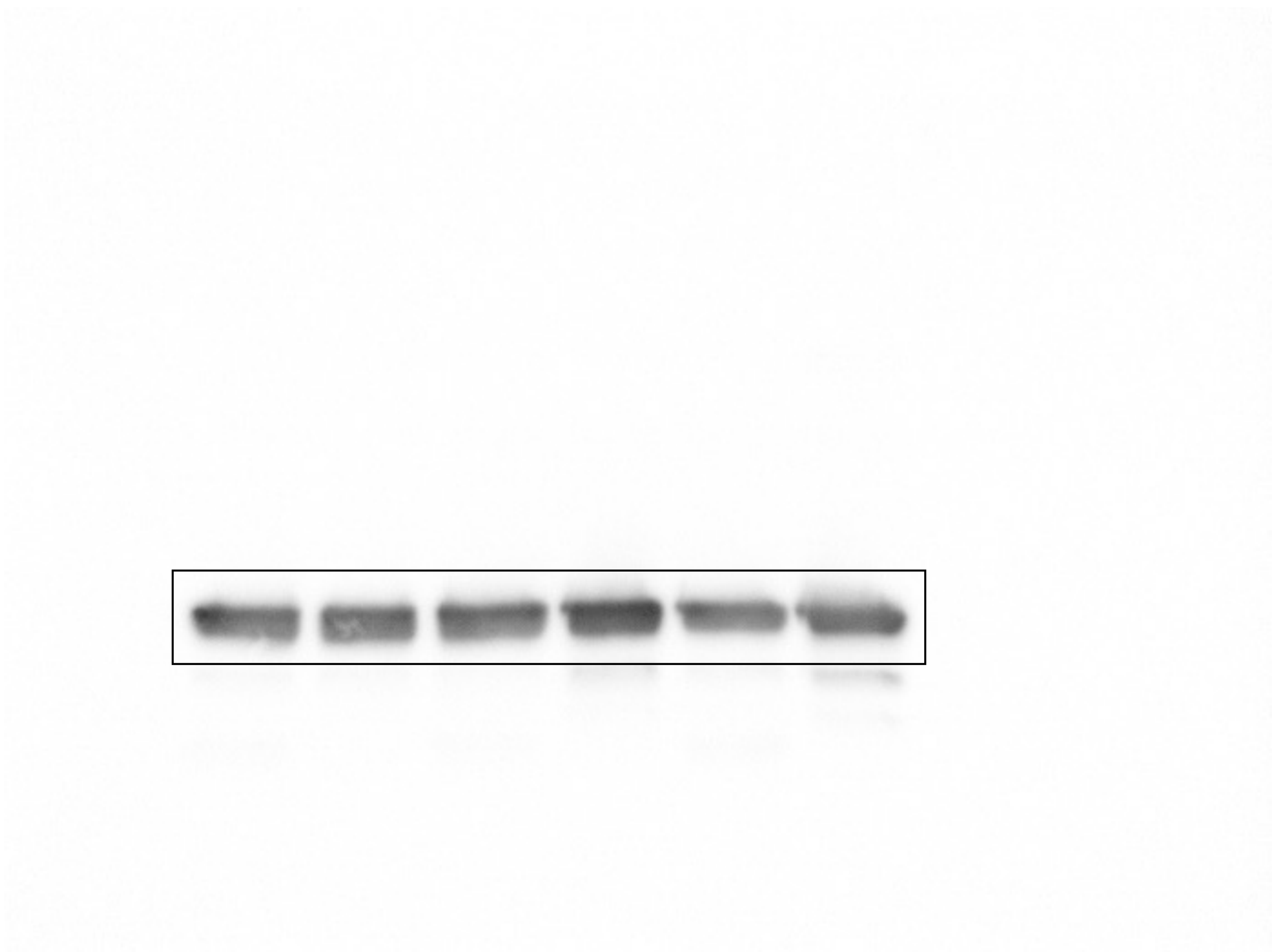

Full unedited blot/gel for Supplement Figure S8A: GAPDH antibody. Note: this is the same GAPDH blot in Figure 1C.
